# Exotoxin-Targeted Drug Modalities as Antibiotic Alternatives

**DOI:** 10.1021/acsinfecdis.1c00296

**Published:** 2022-01-31

**Authors:** Moona Sakari, Arttu Laisi, Arto T. Pulliainen

**Affiliations:** † Institute of Biomedicine, Research Unit for Infection and Immunity, University of Turku, Kiinamyllynkatu 10, FI-20520 Turku, Finland

**Keywords:** exotoxin, bacteria, antivirulence therapy, antibiotics, antibiotic resistance

## Abstract

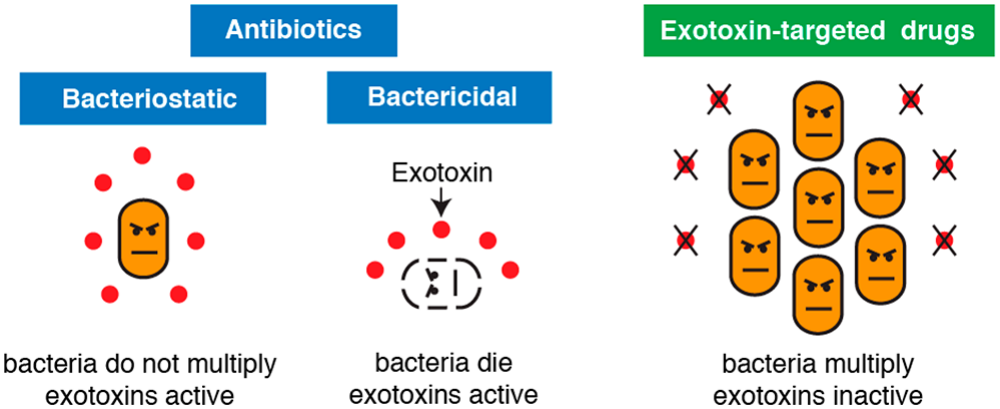

The paradigm of antivirulence therapy dictates that bacterial pathogens are specifically disarmed but not killed by neutralizing their virulence factors. Clearance of the invading pathogen by the immune system is promoted. As compared to antibiotics, the pathogen-selective antivirulence drugs hold promise to minimize collateral damage to the beneficial microbiome. Also, selective pressure for resistance is expected to be lower because bacterial viability is not directly affected. Antivirulence drugs are being developed for stand-alone prophylactic and therapeutic treatments but also for combinatorial use with antibiotics. This Review focuses on drug modalities that target bacterial exotoxins after the secretion or release-upon-lysis. Exotoxins have a significant and sometimes the primary role as the disease-causing virulence factor, and thereby they are attractive targets for drug development. We describe the key pre-clinical and clinical trial data that have led to the approval of currently used exotoxin-targeted drugs, namely the monoclonal antibodies bezlotoxumab (toxin B/TcdB, *Clostridioides difficile*), raxibacumab (anthrax toxin, *Bacillus anthracis*), and obiltoxaximab (anthrax toxin, *Bacillus anthracis*), but also to challenges with some of the promising leads. We also highlight the recent developments in pre-clinical research sector to develop exotoxin-targeted drug modalities, i.e., monoclonal antibodies, antibody fragments, antibody mimetics, receptor analogs, neutralizing scaffolds, dominant-negative mutants, and small molecules. We describe how these exotoxin-targeted drug modalities work with high-resolution structural knowledge and highlight their advantages and disadvantages as antibiotic alternatives.

The paradigm of antivirulence therapy dictates that bacterial pathogens are specifically disarmed but not killed by neutralizing their virulence factors.^1^ Historically, antivirulence therapy precedes the use of antibiotics. The first Nobel Prize in Medicine in 1901 was awarded to Emil von Behring for his work on serum therapy, especially on its application against diphtheria with diphtheria toxin-neutralizing horse antiserum. To some extent, these virulence factor-neutralizing polyvalent antiserum-based drugs are still being used today, e.g.,, diphtheria antitoxin (DAT),^2^ botulism antitoxin heptavalent [A,B,C,D,E,F,G]-[EQUINE] (BAT),^3^ and botulism immune globulin intravenous (BIG-IV/BabyBIG).^4^ In addition, intravenous immunoglobulin (IVIG) preparations that are composed of polyvalent immunoglobulins from pooled plasma samples of thousands of individuals are being developed and used to treat severe diseases, such as necrotizing soft tissue infections, e.g., ref (5) (NCT01790698 and NCT02111161). Decades of basic research using various *in vitro* assays, cell and tissue culture models, and animal experimentation have created an in-depth view on bacterial virulence factors.^6^ It is this molecular and physiological knowledge that is driving the development of next-generation targeted antivirulence therapies involving different modalities.

Exotoxins, a ubiquitous group of secreted or release-upon-lysis bacterial proteins (Figures 1 and 2), have a significant and sometimes the primary role as the disease-causing virulence factor, e.g., in whooping cough, cholera, diphtheria, tetanus, botulism, anthrax, and toxic shock syndrome. Antivirulence drugs are being developed to prevent all the main steps in the functional pathway of exotoxins—expression, secretion, cell surface binding, intracellular maturation, and cytosolic effector functions. One attractive strategy has been to develop small molecules that prevent binding of transcription factors to the promoters of exotoxin-encoding genes and thereby block transcription, as exemplified by the work on staphylococcal transcription factor AgrA.^7^ Inhibitors targeting the Sec-pathway that is responsible for the secretion of the majority of bacterial proteins are alternative antivirulence drug leads, e.g., ref (8). One additional line of research is focused on targeting host cell components, in particular host cell proteins, that are important in the functional pathway of exotoxins. For instance, small molecules have been identified which affect the endosomal maturation,^9^ retrograde trafficking,^10^ intracellular activatory proteolytic processing,^11^ and intracellular chaperone-assisted activatory folding of exotoxins.^12^

This Review is focused on drug modalities, i.e., monoclonal antibodies, antibody fragments, antibody mimetics, receptor analogs, neutralizing scaffolds, dominant-negative mutants, and small molecules, that target bacterial exotoxins after secretion or release-upon-lysis. We describe how these modalities work and highlight their advantages and disadvantages as antibiotic alternatives. Each modality is described with schematic examples where the mode of action is known at atomic resolution (Figures 3–8).

## Bacterial Exotoxins

**Figure 1:**
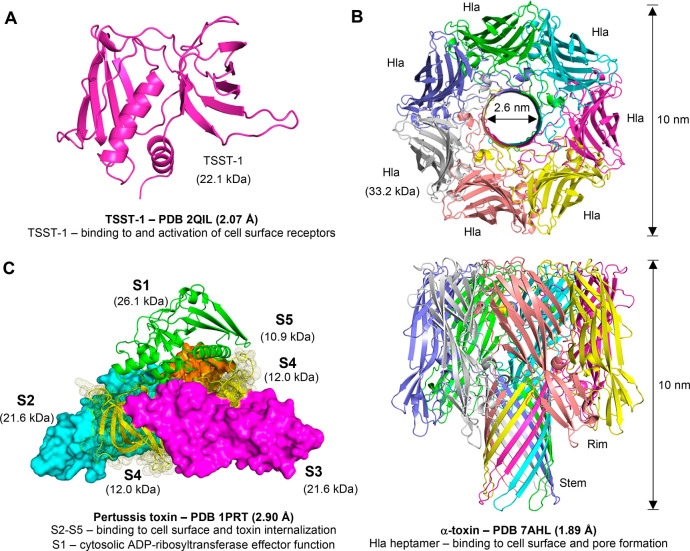
Molecular diversity of bacterial exotoxins. Bacterial exotoxins are a diverse group of monomeric or homo-/heteropolymeric proteins. Three examples are shown. (A) Toxic shock syndrome toxin-1 (TSST-1). The X-ray structure of TSST-1.^151^ The TSST-1 of *S. aureus* (Uniprot P06886) monomer binds simultaneously to MHCII and TCR on the surface of host antigen-presenting cells and T-lymphocytes, respectively. Docking of TSST-1 to MHCII/TCR hyperactivates T-cells, leading to release of high concentrations of cytokines and development of potentially fatal toxic shock syndrome. (B) Pore-forming α-toxin. The X-ray structure of pore-forming α-toxin.^115^ The α-toxin Hla monomers of *S. aureus* (Uniprot P09616) bind to the host cell surface, followed by assembly of homoheptameric structures that protrude across the host cell membrane. Formation of hydrophilic transmembrane channels leads to cell death via osmotic lysis. (C) Pertussis toxin. The X-ray structure of pertussis toxin (S1, green; S2, cyan; S3, magenta; two copies of S4, yellow; S5, orange).^15^ The pertussis toxin of *B. pertussis* (Uniprot P04977–P04981) binds to the host cell surface, gets internalized, and executes its ADP-ribosyltransferase effector function in the cytosol. The S1 subunit of pertussis toxin ADP-ribosylates the inhibitory α subunits of heterotrimeric G proteins, thereby preventing formation of the signal-propagating Gαi-GPCR complex.

Bacterial exotoxins can be classified into three types based on their mode of action: Type I, superantigens; Type II, membrane-disrupting toxins; and Type III, intracellular-targeting toxins. Superantigens, such as toxic shock syndrome toxin-1 (TSST-1) of *Staphylococcus aureus* (Figure 1A),^13^ bind simultaneously to major histocompatibility complex (MHC) class II and T-cell receptor (TCR) molecules on host antigen-presenting cells and T-lymphocytes, respectively. Docking of TSST-1 to MHCII and TCR hyperactivates T-cells, leading to systemic release of inflammatory cytokines and development of potentially fatal toxic shock syndrome.^13^

Membrane-disrupting toxins come in three different flavors. The pore-forming toxins, such as the α-toxin (also known as hemolysin-α or Hla) of *S. aureus* (Figure 1B),^13^ comprise by far the largest group. When the α-toxin of *S. aureus* binds on the host cell surface, it oligomerizes and attacks the cell membrane by extrusion of a β-barrel through the lipid bilayer to form a hydrophilic transmembrane channel and causes cell death via osmotic lysis.^13^ Membrane-disrupting toxins can also act by directly modifying the membrane lipids or by displaying detergent-like functions. The β-toxin (also known as β-hemolysin) of *S. aureus*,^13^ for instance, cleaves sphingomyelin, the abundant eukaryotic membrane sphingolipid. The amphipathic peptides known as phenol-soluble modulins, such as the δ-toxin of *S. aureus*,^13^ integrate into the host cell plasma membrane to cause membrane instability.

**Figure 2:**
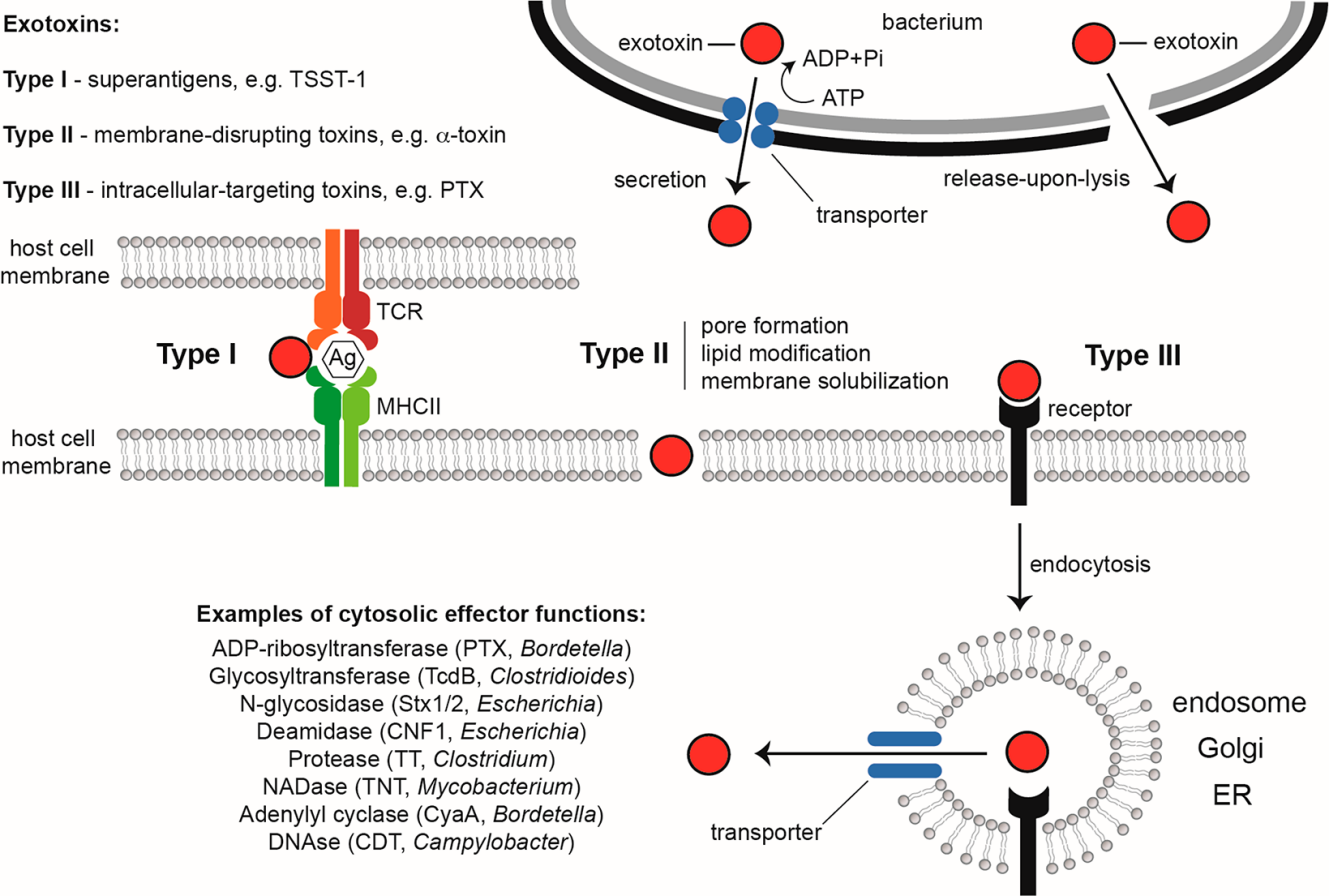
Effector mechanism-based classification of bacterial exotoxins. Exotoxins are bacterial proteins that either are actively secreted from the bacterium in an energy-dependent process or become soluble upon bacterial lysis. Exotoxins recognize the host cell surface via specific receptor structures composed of proteins, lipids, or carbohydrates. Exotoxins have potent host cell modulating activities either at the host cell surface or inside the host cell. Intracellular-targeting toxins undergo a complex maturation process, often involving a retrograde trafficking process from the endosome to the Golgi and ER, followed by effector subunit release into the cytosol. Exotoxins are typically classified in three different types based on their effector mechanisms: Type I, superantigens; Type II, membrane-disrupting toxins (pore-forming toxins, lipid-modifying enzymes, and detergent-like peptides); and Type III, intracellular-targeting toxins. Some overlap exists between these three types, e.g., listeriolysin of *Listeria monocytogenes* or anthrax toxin of *B. anthracis* (see Figure 7B), forming pores in the endosomal membranes. Abbreviations: TCR, T cell receptor; MHCII, major histocompatibility complex class II; Ag, antigen; TSST-1, toxic shock syndrome toxin; PTX, pertussis toxin; TcdB, toxin B; Stx1,2, Shiga toxins 1 and 2; CNF1, cytotoxic necrotizing factor 1; TT, tetanus toxin; TNT, tuberculosis necrotizing toxin; CyaA, bifunctional hemolysin/adenylyl cyclase; CDT, cytolethal distending toxin.

Intracellular-targeting toxins are a diverse group of virulence factors formed of either covalently or non-covalently bound A and B subunits. The A subunit possesses the enzymatic activity, and the B subunits mediate the cell entry. Pertussis toxin (PTX), as an example, is the major virulence factor of *Bordetella pertussis* (Figure 1C),^14^ composed of five non-covalently bound subunits (PtxS1–S5), which are arranged in an AB5 topology.^15,16^ The B5-oligomer is formed by the PtxS2–S5 (PtxS2, PtxS3, PtxS5, and two copies of PtxS4)^15,16^ and mediates binding of the AB5 holotoxin on the host cell surface in a carbohydrate-dependent manner.^16^ Endocytosis-mediated cell entry is followed by retrograde trafficking into the endoplasmic reticulum (ER),^17^ dissociation of the B5-assembly from the PtxS1-subunit,^18^ and ER-associated degradation (ERAD) pathway-dependent transport of PtxS1 into the cytosol.^19^ In the cytosol, PtxS1 ADP-ribosylates a single C-terminal cysteine residue in inhibitory α-subunits of most heterotrimeric (αβγ) G protein superfamily members, such as Gαi, Gαo, and Gαt.^20^ The resulting bulky ADP-ribose modification disrupts inhibitory α-subunit interaction with G protein-coupled receptors (GPCRs), preventing formation of the Gαβγ-GPCR complex and thereby perturbing GPCR agonist-induced signaling.^21^ Other intracellular-targeting toxins follow more or less the same principles as PTX in how they interact with the host cell, i.e., docking into the cell surface receptor, endocytosis, intracellular maturation, and execution of the cytosolic effector function, mostly involving modification of a specific host protein. However, topologies of the AB-assembly vary, e.g., AB (diphtheria toxin), AB5 (pertussis toxin), and A2B5 (typhoid toxin), some toxins such as diphtheria toxin gain access into the cytosol from the endosome, and an array of cytosolic effector functions in addition to protein ADP-ribosylation are executed in the cytosol (Figure 2).

## Interfering with Cell Surface Binding

Binding to the host cell surface, involving recognition of specific receptors, is a necessary functional step for exotoxins (Figure 2). Many exotoxins, such as superantigens and membrane-disrupting toxins, also execute their effector functions at that particular cellular localization. A multitude of different exotoxin-targeted drug modalities, including all of the U.S. Food and Drug Administration (FDA)-approved drugs and most of the clinical trial drug candidates (Table 1), target this step of the functional pathway of exotoxins.

**Table 1:** Exotoxin-Targeted Drugs That Either Are FDA-Approved or Have Entered Clinical Trials^a^

mAb	format	pathogen	target	current state	trial ID
raxibacumab (Abthrax)	h(human)/IgG1	*B. anthracis*	anthrax toxin	FDA 2012 Phase IV	NCT00639678^41^
					CT02016963
					NCT02339155^49^
					NCT02177721
obiltoxaximab (Anthim)	c(chimeric)/ IgG1	*B. anthracis*	anthrax toxin	FDA 2016 Phase IV	NCT00138411
					NCT00829582
					NCT01932242^156^
					NCT01929226^156^
					NCT01453907^156^
					NCT01932437
					NCT01952444^156^
					NCT03088111
bezlotoxumab (Zinplava)	h/IgG1	*C. difficile*	Toxin B (TcdB)	FDA 2016 Phase IV	NCT01241552^24^
					NCT01513239^24^
					NCT04626947
					NCT03880539
					NCT03937999
					NCT03756454
					NCT04415918
					NCT03182907
					NCT03829475
					NCT04317963
					NCT04075422
					NCT04725123
ASN100	2 × h/IgG1	*S. aureus*	α-toxin, five leukocidins	Phase II (terminated)	NCT02940626
					NCT01357213^157^
MEDI4893 (Suvratoxumab)	h/IgG1	*S. aureus*	α-toxin	Phase II	NCT02296320^58^
					NCT01769417
AR-301 (Tosatoxumab)	h/IgG1	*S. aureus*	α-toxin	Phase III	NCT01589185^59^
					NCT03816956
Shigamabs	2 × c/IgG1	*E. coli*	Shiga toxins 1 and 2 (Stx1,2)	Phase II	NCT01252199
TMA-15 (Urtoxazumab)	hIgG1	*E. coli*	Stx2	Phase I	not availabe^71^
XOMA 3Ab	c/IgG1	*C. botulinum*	botulinum neurotoxin A (BoNT/A)	Phase I	NCT01357213^157^
	2 × h/IgG1				
NTM-1632	3 × c/IgG1	*C. botulinum*	BoNT/B	Phase I	NCT02779140
NTM-1634	4 × h/IgG1	*C. botulinum*	BoNT/C-D	Phase I	NCT03046550^158^
NTM-1633	3 × c/IgG1	*C. botulinum*	BoNT/E	Phase I	NCT03603665
S315	h/IgG1	*C. diphteriae*	diphtheria toxin	Phase I	NCT04075175


receptor analog	format	pathogen	target	current state	trial ID
SYNSORB-Pk	polyvalent carbohydrate conjugate	*E. coli*	Stx1,2	Phase III (failed)	NCT00004465^100^


neuralizing scaffold	format	pathogen	target	current state	trial ID
tolevamer	styrene sulfonate polymer	*C. difficile*	TcdA-B	Phase III (failed)	NCT00106509^105^
					NCT00196794^105^
					NCT00382304
					NCT00466635
					NCT00034294
CAL02	liposome	*S. pneumoniae*	pneumolysin	Phase I	NCT02583373^114^


small molecule	format	pathogen	target	current state	trial ID
Ebselen	organoselenium compound	*C. difficile*	TcdA-B	pre-clinical (Phase III)	NCT01452607
					NCT00762671

a Clinical trial data based on ClinicalTrials.gov database, as of March 18, 2021 (https://www.clinicaltrials.gov). Ebselen trials have been conducted in diseases other than *C. difficile* infections, e.g., diabetes Phase III trial NCT00762671.

### Monoclonal Antibodies—Cell Surface Binding

**Figure 3:**
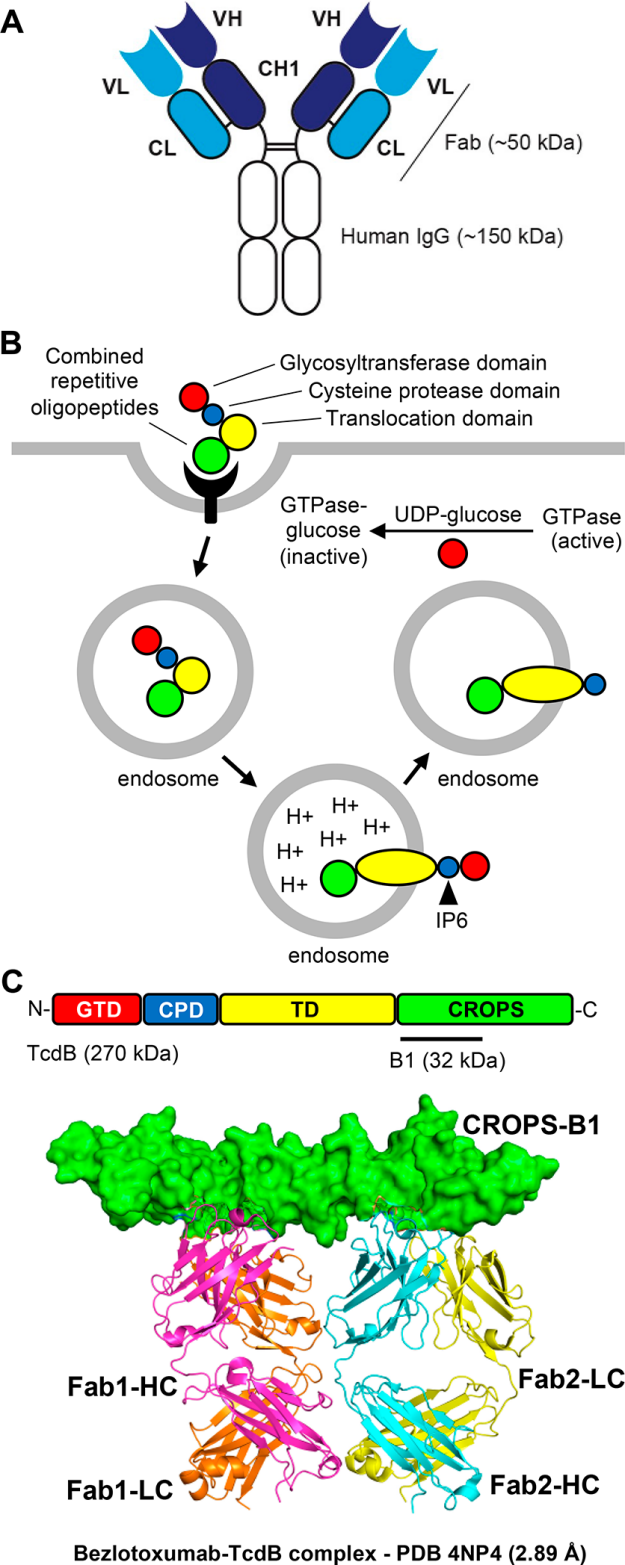
Monoclonal antibodies as exotoxin-targeted drugs: schematic example *Clostridioides difficile* TcdB. (A) Schematic representation of a monoclonal antibody (mAb). All FDA-approved exotoxin-neutralizing drugs are mAbs (Table 1). Key advantages and disadvantages of mAbs as exotoxin-targeted drugs are described in Table 2. Abbreviations: VL, variable light chain; VH, variable heavy chain; CL, constant light chain; CH, constant heavy chain; CH1, constant heavy chain region 1. (B) Host cell intoxication by TcdB. TcdB binds to the host cell surface and gets endocytosed. The CROPS domain of TcdB is involved in recognition of the host cell surface receptor. Acidification of the endosome leads to translocation of the glycosyltransferase domain (GTD) and the cysteine protease domain (CPD) across the endosomal membrane. Cytosolic hexakisphosphate (IP6) binds to and activates the CPD, leading to auto-processing of TcdB. The released GTD catalyzes the transfer of a single glucose moiety to small Rho/Ras GTPases, leading to pathological perturbation of downstream cell signaling responses.^23^ (C) Targeting of *C. difficile* TcdB. Domain structure of *C. difficile* TcdB (Uniprot P18177). The X-ray structure of the N-terminal half of the TcdB CROPS domain bound to the Fab fragments of TcdB-neutralizing bezlotoxumab.^26^ Bezlotoxumab prevents TcdB from binding to its host cell surface receptor. Abbreviations: Fab, fragment antigen binding; LC, light chain; HC, heavy chain.

The monoclonal antibodies (mAbs) have several advantages in exotoxin targeting, such as high specificity, long half-life in circulation, and good tolerability (Table 2). In addition, mAbs do not merely act as passive exotoxin-neutralizing binders, but they also may execute beneficial fragment crystallizable (Fc)-mediated functions, such as complementary interactions and phagocytosis of exotoxin-mAb complexes. Antibody engineering technologies help in the design of enhanced versions, e.g., in affinity and immunogenicity, also involving the possibility to combine two targeting specificities into a single product, i.e., the so-called bispecific antibodies. Low tissue and cell penetration is a drawback of these relatively large molecules (human IgG, ∼150 kDa).^22^ The schematic modality example of mAbs is bezlotoxumab that neutralizes the toxin B (TcdB) of *Clostridioides difficile* (Figure 3C).

**Table 2:** Summary of the Key Advantages and Disadvantages Associated with the Different Exotoxin-Targeted Drug Modalities^a^

modality	advantages	disadvantages
monoclonal antibodies	high target scope	low tissue penetration
	high diversity	low cell permeability
	high specificity	demanding production
	high affinity	high end product price
	high stability	limited routes for administration
	good tolerability	
	long half-life	
	targets immune system to exotoxin	

antibody fragments	high target scope	short half-life
	high diversity	low cell permeability
	high specificity	limited routes for administration
	high affinity	
	high stability	
	good tolerability	
	high tissue penetration	
	ease of production	

antibody mimetics	high target scope	short half-life
	high diversity	low cell permeability
	high specificity	limited routes for administration
	high affinity	
	high stability	
	good tolerability	
	high tissue penetration	
	ease of production	

receptor analogs and neutralizing scaffolds	high target scope	low specificity (off-target effects)
	high diversity	low cell permeability
	high affinity	
	good tolerability	
	ease of production	
	multiple routes for administration	

dominant-negative mutants	high specificity	low target scope
	high affinity	low diversity
		short half-life
		low cell permeability
		limited routes for administration

small molecules	high target scope	short half-life
	high diversity	low specificity (off-target effects)
	high tissue penetration	
	high cell permeability	
	ease of production	
	multiple routes for administration	

a Note that especially the modality “receptor analogs and neutralizing scaffolds” is a highly heterogeneous group, and thus the advantages and disadvantages may vary greatly and need to be assessed case by case. Some canonical features can also be engineered, e.g., to increase the half-life of antibody fragments. To date, most of the pre-clinical research has focused on monoclonal antibodies, antibody fragments, receptor analogs, and neutralizing scaffolds. All the currently FDA-approved exotoxin-targeted drugs are monoclonal antibodies. Only a few small molecules that specifically target exotoxins have been reported. This is in striking contrast with the dominance of small molecules in the development pipelines of pharmaceuticals in other pathologies.

#### Bezlotoxumab (Zinplava)

*C. difficile* infection (CDI) is the most common cause of infectious diarrhea among hospitalized patients. It is caused by an anaerobic, Gram-positive, spore-forming bacterium, and the disease usually follows antibiotic treatment due to dysbiosis of gut microbiota.^23^*C. difficile*-induced colitis is commonly treated with enteral vancomycin, fidaxomicin, and metronidazole, but after the primary treatment approximately 30% of patients have recurrent disease episodes.^24^ The major disease-causing virulence factors of *C. difficile* are the two homologous clostridial exotoxins, toxin A (TcdA) and toxin B (TcdB).^23^ The host cell intoxication mechanism of TcdB is schematically described in Figure 3B.

Bezlotoxumab is a TcdB-binding human mAb, which was identified via screening of hybridomas of TcdB-vaccinated HuMAb mice.^25^ Bezlotoxumab binds to the combined repetitive oligopeptides (CROPS) domain and prevents TcdB from binding to its receptor^26,27^ (Figure 3C). During the development of bezlotoxumab, also an anti-TcdA human mAb (actoxumab) with a mode of action similar to that of bezlotoxumab was identified,^25,28^ but it was later shown to lack efficacy in CDI.^24^ In pre-clinical cell culture-based studies, bezlotoxumab, and also actoxumab, neutralized toxin activities of several *C. difficile* strains.^29^ In multiple murine models of CDI, an intraperitoneally administered prophylactic actoxumab–bezlotoxumab mixture reduced tissue damage and inflammatory response in the gut wall.^30^

The pharmacokinetics and safety of bezlotoxumab in humans were evaluated in two large multicenter trials.^24^ The safety profile of bezlotoxumab was similar to that of placebo.^24^ In Phase II study, the combination of actoxumab and bezlotoxumab lowered the risk of recurrent CDI among patients who also received standard-of-care when compared to placebo.^31^ Phase III trials for actoxumab and bezlotoxumab included two international, multicenter, double-blind, randomized, and placebo-controlled studies (MODIFY I and MODIFY II), in which the effects of actoxumab and bezlotoxumab were studied on patients with primary or recurrent CDI.^24^ The primary end point in these studies was recurrent infection, i.e., new episode after initial clinical cure, within 12 weeks after infusion. In both trials the risk of recurrent CDI was significantly lower in the bezlotoxumab group than in the placebo group (MODIFY I, 17% vs 28%; MODIFY II, 16% vs 26%). Subgroup analyses revealed that, in the subpopulations at high risk for recurrent infection (age >65, history of CDI, compromised immunity, severe CDI) or for an adverse outcome, groups that received bezlotoxumab had a lower rate of recurrent infection than the placebos. Among high-risk patients, who were hospitalized at the time of infusion, bezlotoxumab decreased the rate of hospital re-admission within 30 days. However, bezlotoxumab or actoxumab did not increase the probability on initial clinical cure. It was also shown that the patients who had no risk factors for recurrent CDI did not benefit from additional treatment with bezlotoxumab. Recently, more analysis of the MODIFY I,II data has been published, e.g., refs (32 and 33), that together with the real-world efficacy analysis in clinical practice, such as in Finland,^34^ supports the clinical use of bezlotoxumab in CDI. Even though the cost of bezlotoxumab treatment is not negligible, cost-effectiveness analyses favor treatment of CDI with bezlotoxumab.^35^

Bezlotoxumab was FDA-approved in 2016 for use in clinical practice to reduce the recurrence of CDI in adult patients (18 years or older) who are treated with standard-of-care antibiotics for CDI and are at high risk for CDI. Bezlotoxumab is administered via intravenous infusion [package insert - Zinplava (bezlotoxumab), Merck & Co, Inc., Whitehouse Station, NJ, 2016]. According to ClinicalsTrial.gov, there are five Phase IV (NCT04626947, NCT03880539, NCT03937999, NCT03756454, NCT04415918), one Phase III (NCT03182907), one Phase II (NCT03829475), and two case-control studies (NCT04317963, NCT04075422) ongoing with connection to bezlotoxumab. All trials are currently in the recruiting phase.

#### Raxibacumab (Abthrax) and Obiltoxaximab (Anthim)

Anthrax is a rare but potentially lethal disease caused by the rod-shaped, Gram-positive, spore-forming bacterium *Bacillus anthracis*. Inhalational anthrax drew global attention after the 2001 bioterrorist attacks in the U.S., which resulted in 11 confirmed cases and five fatalities. The pathogenesis of inhalational anthrax is driven by the tripartite anthrax toxin complex.^36^ The host cell intoxication mechanism of anthrax toxin is schematically described in Figure 7B.

Obiltoxaximab is a chimeric protective antigen (PA)-recognizing mAb, which has been engineered for higher affinity and for lower immunogenicity,^37^ building on the early work on mouse anthrax toxin-neutralizing antibodies^38^ and mAb–PA interaction affinity-enhancing mutations.^39^ It is known, in particular based on the work on its parental murine forms, that obiltoxaximab recognizes the receptor-binding region of PA^40^ and thereby blocks PA–host cell receptor interactions. Raxibacumab is a fully human mAb binding to the PA and acts in analogy to obiltoxaximab.^41^

Obiltoxaximab was well-tolerated among healthy volunteers in Phase I trials, and the most common adverse events included upper respiratory tract infections and hypersensitivity reactions.^42^ The safety, tolerability, and pharmacokinetics of raxibacumab in humans were evaluated with healthy volunteers in four sub-studies performed by Human Genome Sciences.^41,43^ These studies concluded that raxibacumab is safe, well-tolerated, and bioavailable after single intramuscular or intravenous dose.^41,43^ Most adverse events were mild to moderate in severity and did not significantly differ from placebo.^41,43^

The FDA Animal Rule allows drug approval in the well-justified cases where human efficacy studies are unethical, such as with anthrax. The efficacies of raxibacumab and obiltoxaximab were evaluated with animal experimentation utilizing rats, rabbits, dogs, and macaques under the FDA Animal Rule. Rats that received a prophylactic dosage of raxibacumab 24 h prior to toxin infusion had a survival rate of 100%, whereas all rats in the placebo group died.^41^ In a study conducted with rabbits, animals receiving intravenous infusion of obiltoxaximab prior to exposure to anthrax spores had a survival rate of 100%, whereas all saline-treated animals in control group died.^37^ Initial therapeutic studies conducted in rats showed that raxibacumab increased survival when administered within 6 h after the toxin infusion.^44^ The survival rate was lower in rats that received raxibacumab at 9 or 12 h after the toxin infusion, and the survival rate also decreased with lower doses of raxibacumab.^44^ Rabbits that received obiltoxaximab 24 h after the toxin exposure had a survival rate of 80%, but when obiltoxaximab was given at 36 h the survival rate decreased to 50%.^37^ In the macaque mode, both raxibacumab and obiltoxaximab, given either prophylactically or therapeutically, increased survival rates, and the increase was dose-dependent.^41,45^

Combinatorial use of anthrax-toxin-neutralizing mAbs with antibiotics, supportive care, and anthrax toxin vaccination has been studied by animal experimentation and clinical trials. The data in rabbits indicates that combining raxibacumab to levofloxacin improves survival compared to levofloxacin therapy alone.^46^ Rabbit studies also support the use of an obiltoxaximab–doxycycline combination.^47^ In studies with a canine model of anthrax toxin-associated shock, it was shown that combination of hemodynamic support, i.e., titrated normal saline and norepinephrine infusions, and raxibacumab significantly improved survival compared to hemodynamic support alone.^48^ The FDA-approved anthrax vaccine, anthrax vaccine absorbed (AVA), is mainly composed of adsorbed PA. In a recent open-label, randomized, multicenter study, it was concluded that co-administering raxibacumab with AVA does not reduce immunogenicity of AVA.^49^

Raxibacumab and obiltoxaximab got their FDA approvals in December 2012 and March 2016, respectively. Both drugs are now indicated in adult and pediatric patients for the treatment of inhalational anthrax in combination with appropriate antibiotics, e.g., levofloxacin or doxycyclin, and for prophylaxis of inhalational anthrax when alternative options are not available or are not appropriate. The recommended method of administration is intravenous infusion, and patients should be pre-medicated with oral or intravenous diphenhydramine to reduce the risk of infusion reactions [package inserts - Abthrax (raxibacumab), Human Genome Sciences, Inc., Rockville, MD, 2012; Anthim (obiltoxaximab), Elusys Therapeutics, Inc., Pine Brook, NJ, 2016]. According to ClinicalsTrial.gov, there are currently two Phase IV clinical trials with an objective to evaluate clinical benefit, safety, and pharmacokinetics in patients treated with raxibacumab (NCT02177721) or obiltoxaximab (NCT03088111).

#### ASN100

*S. aureus* is a Gram-positive common bacterial commensal of humans. It is also a major opportunistic pathogen, and the global disease burden of *S. aureus* infections is remarkable. Despite the appropriate antibiotic treatment, the mortality in severe infections remain high. The appearance of methicillin- and vancomycin-resistant *S. aureus* strains is concerning, as infections are becoming more demanding to treat.^50^*S. aureus* produces tens of different exotoxins, which can be divided into three major groups: exfoliative toxins, superantigens, and membrane-disrupting toxins such as the α-toxin and leukocidins.^13^ Perhaps the most renowned *S. aureus* toxin is the pore-forming α-toxin, also known as α-hemolysin or Hla (Figure 1B). It is secreted as a monomer by a majority of clinical *S. aureus* strains.^13^ After binding to a receptor on the target cell surface, it oligomerizes and forms a transmembrane β-barrel pore, leading to profound cellular effects and eventually cell lysis.^13^ There are five leukocidins in *S. aureus* strains associated with human infections: Panton–Valentine leukocidin (PVL), LukAB, LukED, and two γ-hemolysins, HlgAB and HlgCB.^13^ Leukocidins are composed of two protein subunits, designated as S- and F-subunits.^13^ The S-subunits bind to the host cell surface receptor, leading to recruitment of and dimerization with the F-subunits.^13^ Oligomerization of the S/F-subunit dimers results in the transmembrane leukocidin pore formation.^13^

The α-toxin- and leukocidin-neutralizing ASN100 was developed based on screening of a high-diversity yeast surface displayed in human IgG1 libraries.^51,52^ ASN100 is composed of two fully human IgG1 mAbs, ASN-1^51^ and ASN-2.^52^ ASN-1 neutralizes α-toxin and the leukocidins PLV, LukED, HlgAB, and HlgCB via a common conformational epitope shared between α-toxin and leukocidin F-subunits.^51^ The apparent mode of action is masking of the phosphocholine-binding pockets of α-toxin and leukocidin F-subunits and thereby prevention of membrane interactions.^51^ ASN-2 neutralizes the fifth leukocidin, LukAB.^52^ Interestingly, ASN-2 recognizes the S- and F-subunit dimeric structure yet leads to the same mode of action as ASN-1, preventing leukocidin interactions with the target cells.

In the first pre-clinical *in vitro* studies, ASN-1 inhibited α-toxin-mediated lysis of epithelial cells and leukocidin-mediated destruction of phagocytes and human erythrocytes.^51^ ASN-2 protected polymorphonuclear phagocytes from LukAB-mediated lysis.^52^ Both ASN-1 and ASN-2 were needed to protect human leukocytes from cytotoxicity after exposure of culture supernatants of *S. aureus* strains.^53^ ASN100, but also ASN-1 alone, was able to protect the morphology of 3D human tracheal/bronchial mucociliary epithelial tissue culture infected with *S. aureus*.^53^ In murine models, administration of ASN-1 before intranasal or intravenous challenge with *S. aureus* prevented lethal pneumonia and sepsis.^51^ Also a therapeutic effect was observed when ASN-1 was administered 2 h after intranasal challenge in combination with the linezolid antibiotic.^51^ In another study, ASN100 increased survival in a dose-dependent manner when given intravenously prior to intratracheal exposure of *S. aureus* in a rabbit *S. aureus* pneumonia model.^54^ Also reduced macroscopic and microscopic lung pathology and bacterial burden were observed.^54^ Pharmacokinetic analysis of bronchoalveolar lavage (BAL) fluid showed penetration of ASN100 to lung epithelial lining fluid at 24 h after administration with peak levels at 48 h.^54^

The safety, tolerability, and pharmacokinetics of ASN100 were evaluated in a randomized, double-blind, Phase I study with healthy volunteers.^55^ No dose-limiting toxicities were observed during the study. All adverse events were mild or moderate in severity and resolved without medical interventions. ASN-1 and ASN-2 seemed to have linear pharmacokinetics, with a half-life of 20–36 days after intravenous administration. Both components were detectable in BAL fluid already at 24 or 48 h and remained detectable at least up to day 30. Also, the toxin neutralization activity of ASN-1 and ASN-2 was preserved in human sera.^55^

The effect of ASN100 for prevention of *S. aureus* pneumonia in mechanically ventilated patients was studied in a multicenter, double-blind, single-dose, placebo-controlled trial (NCT02940626, study duration 2016–2018). In this study, participants (*n* = 155) were selected by culturing an endotracheal aspirate to identify those who are heavily colonized with *S. aureus*. Subjects were randomized to receive either ASN100 or placebo. The primary end point was to determine the proportion of patients who had or had not developed *S. aureus* pneumonia after a single intravenous dose of ASN100. After pre-planned interim analysis of 118 subjects, the data review committee was informed that the study was unlikely to meet its primary end point, and the trial was terminated. However, patients were followed for adverse effects after the trial termination. The results of the Phase III trial have not been published, nor it is known how the AS100 development pipeline is being continued.

There are also other *S. aureus* exotoxin-targeted mAbs in clinical trials (Table 1). MEDI4893 (suvratoxumab) is a human mAb that binds to *S. aureus* α-toxin, sterically preventing host cell surface receptor binding and thereby subsequent α-toxin oligomerization.^56^ In a mouse model of *S. aureus* pneumonia, prophylactic MEDI4893 decreased mortality and bacterial burden in the lungs.^57^ In a Phase I trial, MEDI4893 was well-tolerated among subjects, and no serious adverse effects were reported.^58^ A Phase II trial of MEDI4893 (NCT02296320, study duration 2014–2018) has been conducted. No publications on this study have been released. AR-301, also known as Salvecin, is another mAb that binds and neutralizes α-toxin. No pre-clinical data has been published, but it is known that AR-301 was discovered by screening the B cell repertoire of *S. aureus* pneumonia patients for mAbs with α-toxin-neutralizing activity.^59^ Treatment of *S. aureus*-challenged mice with AR-301 either prophylactically or therapeutically was effective.^59^ In a Phase I/II trial, the safety and efficacy of AR-301 were evaluated with intensive care unit patients with severe microbiologically confirmed *S. aureus* pneumonia. The results showed that AR-301 was well-tolerated, and no serious adverse effects were reported. In a subgroup analysis of patients with ventilator-associated bacterial pneumonia, the ventilation duration was shorter among patients who received AR-301 as compared to placebo.^59^ The Phase III trial of AR-301 is currently in the recruiting phase (NCT03816956).

#### Shigamabs

Some strains of *Escherichia coli*, such as Shiga toxin-producing *E. coli* (STEC), can cause a severe foodborne disease. Clinical manifestations of STEC infections vary from asymptomatic carriage to severe hemorrhagic colitis. The most severe complication of STEC infection is hemolytic uremic syndrome (HUS), which is a thrombotic disorder, characterized by microvascular thrombi, microangiopathic hemolytic anemia, thrombocytopenia, and acute renal failure. A significant portion of patients suffering from HUS need renal dialysis, and particularly children and the elderly are more susceptible to complications and death.^60^ Administration of antibiotics in these STEC infections has long been controversially associated with increased risk of HUS. In a recent review article,^61^ it was concluded that the risk of HUS seems to be associated with the particular STEC strain causing the infection and the antibiotic class used in the treatment. Because of the potential negative effect of antibiotics, other alternative therapeutic agents against STEC have been under development, and the first Shiga toxin-neutralizing mAbs were introduced in the 1980s, e.g., ref (62). *E. coli* Shiga toxins 1 and 2 (Stx1,2) and the *Shigella dysenteriae* Shiga toxin (Stx) are AB5 topology exotoxins with extremely potent cytotoxicity.^63^ The host cell intoxication mechanism of Shiga toxins is schematically described in Figure 6B.

Shigamabs is a combination of two chimeric mAbs, cαStx1 and cαStx2, which recognize and neutralize Stx1 and Stx2, respectively.^64^ The development pipeline is based on mouse mAbs, namely the Stx1 B-subunit recognizing 13C4^62^ and the Stx2 A-subunit recognizing 11E10.^65^ The 13C4 mAb neutralizes Stx1 via blockage of Stx1–host cell receptor interaction,^66^ whereas 11E10 appears to alter the sub-cellular trafficking of Stx2.^67^ Thorough efficacy studies of Shigamabs in mice have been published.^64^ During the study, mice were either orally infected with a lethal dose of Stx2-producing STEC strain B2F1 or intraperitoneally injected with purified Stx1 and/or Stx2 with median lethal dose (LD_50_). Intravenously administered cαStx1 and cαStx2 protected the mice when given either before or after Stx1 and Stx2 injections, respectively. In mice infected with B2F1, intravenous cαStx2 protected the mice when given at 24 or 48 h after the infection. The cαStx2 was also proven to be effective when administered intramuscularly. In mice that were injected simultaneously with Stx1 and Stx2, both cαStx1 and cαStx2 were required to protect the mice. Mice that received a combination of cαStx1 and cαStx2 1 h prior to intoxication had a survival rate of 70%.^64^

The tolerability and pharmacokinetics of cαStx2 were evaluated in a Phase I trial.^68^ In this open-label, non-randomized study, 17 healthy volunteers were divided in four groups to receive escalating doses of cαStx2 by intravenous infusion. Among the subjects, the most common adverse effect was headache, which was reported by nine volunteers. Anti-chimeric antibodies were detected in four volunteers on day 56. The tolerability and pharmacokinetics of cαStx1 were evaluated in two single-center, open-label, non-randomized, dose-escalation Phase I studies.^69^ Also, the safety of combined infusion of cαStx1 and cαStx2 was evaluated. Subjects (*n* = 26) were healthy adult volunteers who received an intravenous infusion of cαStx1, cαStx2, or both. The most common adverse effects, reported by 18 volunteers, were headache and mild somnolence, symptoms of upper respiratory tract infections, and gastrointestinal inconveniences. The pharmacokinetic profiles of both cαStx1 and cαStx2 were similar, and simultaneous infusion of both antibodies did not have an effect on the pharmacokinetics. Anti-chimeric antibodies were only detected on day 57 in one volunteer, who had received cαStx2.

The safety, tolerability, and efficacy of Shigamab were evaluated in a randomized, placebo-controlled, multicenter Phase II trial (SHIGATEC, NCT01252199). The subjects (*n* = 45) were children aged between 6 months and 18 years, diagnosed with Shiga toxin-producing bacterial infection and bloody diarrhea. The results have not been released, but Shigamabs was mentioned in one review article to be well-tolerated and safe.^70^ Shigamabs was developed by Thallion Pharmaceuticals Inc. in collaboration with LFB Biotechnologies. In 2013, it was announced that the collaboration between Thallion and LFB ended, and all the rights of the Shigamabs program reverted to Thallion. However, in 2017, Sun Pharmaceutical Industries Ltd. acquired Thallion, and the transaction is believed to assist the development of Shigamabs. At the time of the acquisition, Sun Pharma estimated that the commercialization of Shigamabs would take around 7–8 years. There is a possibility that the financial circumstances between Thallion, LFB, and Sun Pharma have an impact on the developmental pipeline of Shigamabs as well as to the release of data on the clinical trials.

Several other mAbs against Shiga toxins have also been developed. Most notably, the Stx2-binding TMA-15, also known as urtoxazumab, proceeded to Phase I trial and was safe and well-tolerated in humans.^71^ This developmental pipeline is based on humanized mouse mAb, VTm1.1,^72^ which binds to the pentameric B-subunit of Stx2. In pre-clinical studies, treatment with TMA-15 up to 24 h after infection ameliorated the lethal Stx2-producing STEC strain B2F1 challenge in mice.^73^ However, the urtoxazumab dosage needed to protect the STEC-infected mice appears to be significantly higher as compared to that of cαStx2.^64^ The efficacy of urtoxazumab has also been evaluated in a gnotobiotic piglet model, and the results indicate that urtoxazumab reduces post-infection neurological sequelae.^74^ The developmental future of urtoxazumab remains unclear.

#### hu1B7/hu11E6

In addition to the FDA-approved and the clinical trial mAbs (Table 1), there are a number of exotoxin-targeted mAbs in pre-clinical development (Table S1). Many of these are in an early state. A notable difference is the developmental pipeline focused on pertussis toxin, which is the major virulence factor of *B. pertussis*.^14^ The Gram-negative bacterium *B. pertussis* is the etiological agent of the whooping cough, i.e., pertussis. Whooping cough is a globally distributed acute respiratory disease, affecting all age groups.^75^ However, infants and young children comprise the highest risk cohort, where the disease may lead to death despite hospital intensive care and use of antibiotics.^75^ Especially young children who still lack the vaccine-induced protection against whooping cough could benefit from pertussis toxin-neutralizing mAbs. The young whooping cough patients, in contrast to adults, are typically diagnosed very early and thereby could possess a therapeutic window to interfere with the pertussis toxin-induced pathology. Exposed family members of the whooping cough patients could be an additional patient group subjected to a prophylactic administration of pertussis toxin mAbs, possibly in combination with antibiotics.

Humanized pertussis toxin-neutralizing monoclonal antibodies hu1B7 and hu11E6 were developed^76−78^ and also combined into a single bispecific mAb,^79^ building on the early mouse anti-pertussis toxin antibody studies, e.g., ref (80). Both hu1B7 and hu11E6 antibodies, either individually or as a cocktail, form multivalent complexes with soluble pertussis toxin that bind the IgG receptor more tightly than antibodies alone.^77^ This indicates that the antibodies could accelerate pertussis toxin clearance via immune complex formation. However, hu11E6, and to some extent hu1B7, also prevents pertussis toxin binding to its cell surface receptor. In addition, hu1B7 appears to trap pertussis toxin at or near the cell surface by interfering either with endocytosis or with the early steps in retrograde trafficking of pertussis toxin.^77^ It is very encouraging that a hu1B7/hu11E6 cocktail has a prophylactic and therapeutic effect in mouse (intraperitoneal route) and adult baboon (intravenous route) pertussis models, respectively.^78^ Moreover, the most recent experimentation with hu1B7 intravenous monotherapy in an infant baboon pertussis model demonstrates a potent prophylactic effect.^76^

### Antibody Fragments—Cell Surface Binding

**Figure 4:**
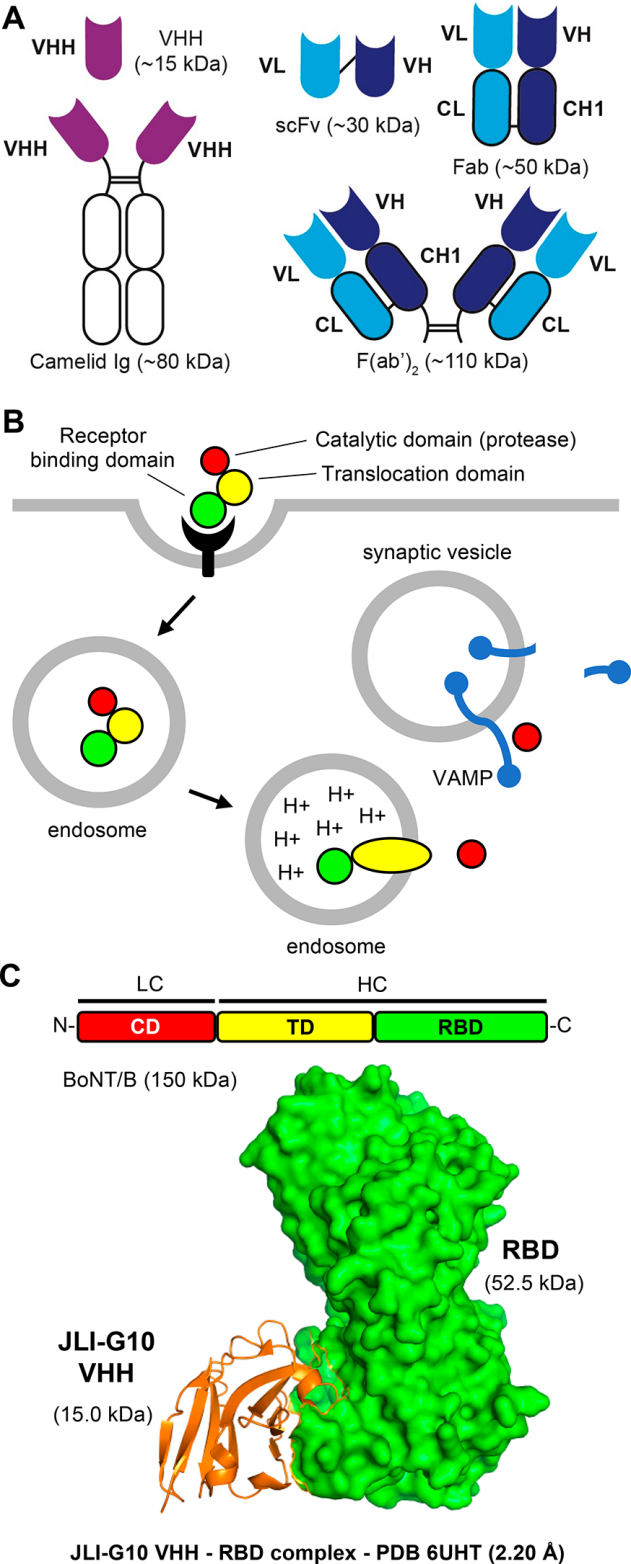
Antibody fragments as exotoxin-targeted drugs: schematic example *Clostridium botulinum* BoNT/B. (A) Schematic representation of antibody fragments. Key advantages and disadvantages of antibody fragments as exotoxin-targeted drugs are described in Table 2. Abbreviations: VHH, variable heavy homodimer of camelid immunoglobulins; Fab, fragment antigen binding; scFv, single-chain fragment variable; VL, variable light chain; VH, variable heavy chain; CL, constant light chain; CH, constant heavy chain; CH1, constant heavy chain region 1. (B) Host cell intoxication by botulinum neurotoxins. BoNT/B binds to the host cell surface and gets endocytosed. Acidification of the endosome leads to activation of the translocation domain (TD) and translocation of the catalytic domain (CD) into the cytosol. Reduction of disulfide bond releases the CD from the TD. The released CD of BoNT/B cleaves proteolytically the vesicle-associated membrane proteins (VAMPs) on the surface of synaptic vesicles. This prevents the fusion of the synaptic vesicle with the pre-synaptic membrane and thereby the release of neurotransmitters, leading eventually to neuroparalysis. (C) Targeting of *C. botulinum* BoNT/B. Domain structure of *C. botulinum* BoNT/B (Uniprot P10844). The BoNT/B molecule is composed of a light chain (LC, the protease domain) and a heavy chain (HC), which is comprised of the N-terminal translocation domain (TD) and the C-terminal receptor binding domain (RBD). The X-ray structure of the receptor binding domain of BoNT/B bound to the VHH JLI-G10.^86^ The JLI-G10 prevents BoNT/B from binding to its host cell surface receptors.

Antibody fragments include the mono- and bivalent fragment antigen-binding (Fab) and F(ab′)_2_, respectively, single-chain fragment variable (scFv), and single-domain antibodies, i.e., variable heavy homodimer (VHH) nanobodies derived from the heavy-chain-only camelid immunoglobulins^81^ (Figure 4A). The VHHs, Fabs, and scFvs are often used in phage display selections and for initial characterization but were eventually engineered to various Ig-like fusions, as exemplified by the work done on staphylococcal superantigenic exotoxin B,^82^ clostridial TcdB,^83^ and botulinum neurotoxin type A (BoNT/A).^84^ Antibody fragments can offer several advantages over the use of conventional mAbs (Table 2). They can be produced more easily, generally using microbial expression systems, which results in faster cultivation, higher yields, and lower production costs. Their small size also allows better tissue penetration. A major drawback is a short serum half-life, which, however, can be engineered. The schematic modality example of antibody fragments is the bifunctional JLI-G10 VHH that neutralizes the botulinum neurotoxin B (BoNT/B) of *Clostridium botulinum* (Figure 4C). Botulinum neurotoxins (BoNTs), produced by the anaerobic bacterium *C. botulinum* and related species, are among the most potent exotoxins classified into seven serotypes (BoNT/A–G).^85^ The host cell intoxication mechanism of BoNTs is schematically described in Figure 4B.

In a recent work,^86^ high-resolution structures and neutralizing mechanisms of unique VHHs against BoNT/A1 and BoNT/B1 of *C. botulinum* were investigated. The BoNT/B-targeting VHHs bound to the C-terminal subdomain of BoNT/B, e.g., JLI-G10 VHH (Figure 4C), in particular in such a way that the BoNT/B–host cell receptor interactions were prevented. In contrast, BoNT/A-targeting VHHs either blocked the membrane insertion of the translocation domain or interfered with the unfolding of the protease domain. By connecting two VHHs of complementary neutralizing mechanism with flexible spacers, bifunctional VHH heterodimers (VHH-based neutralizing agents, VNAs) were created. These VNAs, with a dual epitope binding mode, showed superior potency in mouse BoNT/A or BoNT/B co-intoxication assay, i.e., toxins and VHHs mixed prior to intraperitoneal injection, as compared to the same monomeric VHHs. Moreover, the VNAs also protected mice against BoNT/A1 and BoNT/B1 when administered 30 or 60 min prior to toxins.

### Antibody Mimetics—Cell Surface Binding

**Figure 5:**
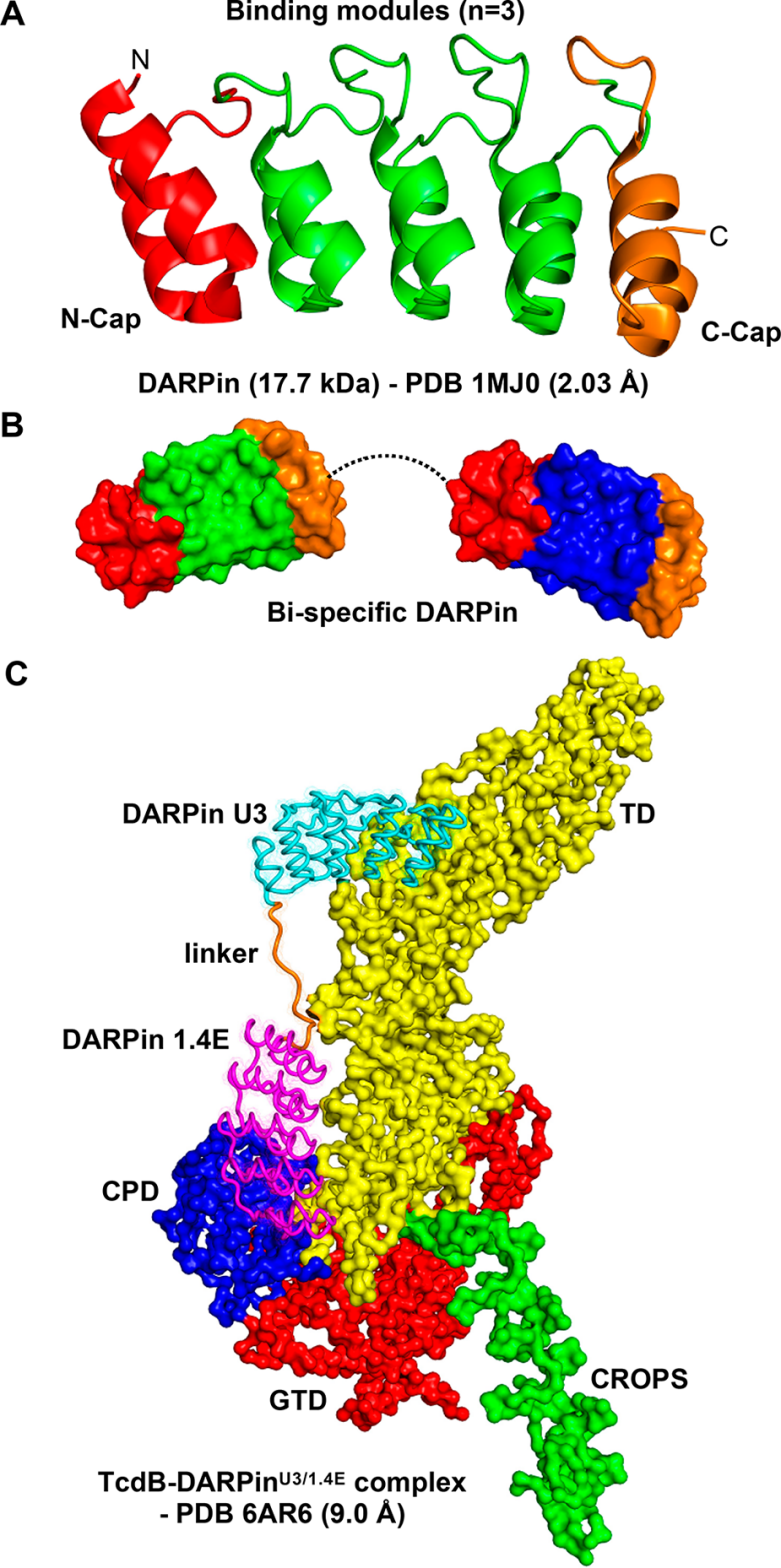
Antibody mimetics as exotoxin-targeted drugs: schematic example *C. difficile* TcdB. Key advantages and disadvantages of antibody mimetics, such as DARPins (designed ankyrin repeat proteins) and Centyrins, are described in Table 2. (A) Schematic representation of a DARPin. The X-ray structure of E3.5 DARPin^152^ illustrates the general fold and modularity of DARPins.^89^ DARPin libraries are composed of the constant caps (N- and C-cap) and a varying number of binding modules, typically three as in the E3.5 DARPin. Amino acid sequences of the binding modules vary in DARPin libraries, allowing screening of different target-recognizing DARPins. (B) Schematic representation of a bispecific DARPin. Bispecific or multispecific DARPins, connected with a flexible linker, can be engineered to simultaneously bind different epitopes of the same target or different targets.^89^ (C) Bispecific DARPin that neutralizes the TcdB of *C. difficile*. Cryo-EM structure of the full-length TcdB in complex with bispecific DLD-4 DARPin.^90^ The DLD-4 is based on U3 and 1.4E DARPins binding to different epitopes in TcdB. The U3 DARPin interacts with the translocation domain (TD) and the 1.4E DARPin with both the TD and the cysteine protease domain (CPD). Refer to the domain structure of TcdB in Figure 3C. The TcdB neutralization potency of DLD-4 derives from its ability to interfere with the interaction between TcdB and its cell surface receptors.^90^

Antibody mimetics are a heterogeneous group of scaffold molecules such as the designed ankyrin repeat proteins (DARPins) and the fibronectin type III domain-based Centyrins. Antibody mimetics are able to overcome some of the limitations of mAbs while still possessing many of their benefits, e.g., high target binding affinity and specificity^87^ (Table 2). Antibody mimetics are small (<20 kDa), single-domain scaffolds that are thermostable and highly engineerable and can be produced in microorganisms or even be synthesized chemically. As many of these scaffolds are derived from human proteins, they possess low immunogenicity. Owing to their small size, they have relatively good tissue penetration. Their serum half-life is short. However, this can be extended, e.g., with polyethylene glycosylation (PEGylation) or conjugation with serum albumin.^88^ The schematic modality example of antibody mimetics is the bispecific DLD-4 DARPin that neutralizes the TcdB of *C. difficile* (Figure 5C).

DARPins are derived from natural ankyrin repeat proteins, which are among the most abundant binding proteins found in the human genome.^89^ DARPins are small, single-domain proteins (∼15 kDa) consisting of three repeat modules: an N-terminal capping repeat (N-cap), a varying number of internal ankyrin repeats, and a C-terminal capping repeat (C-cap) (Figure 5A,B). A series of monomeric and dimeric DARPins with potent neutralization activity for *C. difficile* TcdB was developed^90,91^ (Figure 5C). The monomeric DARPins against TcdB, e.g., U3 and 1.4E DARPins, interfered with the interaction between TcdB and its receptors, chondroitin sulfate proteoglycan 4 (CSPG4) and Frizzled receptor 2 (FZD2), respectively, by binding to the delivery domain of TcdB. The dimeric DLD-4, composed of U3 and 1.4E DARPins, had superior TcdB-neutralization potencies as compared to the FDA-approved mAb bezlotoxumab (see Figure 3C). The *in vivo* efficacy of the dimeric DLD-4 was also studied against TcdB challenge in intraperitoneal injection and cecum injection mouse models. A significant increase in survival was monitored with intraperitoneal injection upon pre-incubation of TcdB with DLD-4. However, only a minor survival advantage was observed with the cecum injection model in mice receiving a combination of TcdB and DLD-4 compared to TcdB alone. This was apparently due to the poor resistance of DLD-4 against the gut protease activity. This shortcoming might be overcome by engineering protease-stable DAPRin variants. It remains unclear whether the DARPins would attenuate TcdB-induced symptoms after a systemic TcdB exposure.

Centyrins are small (∼10 kDa) globular proteins derived from a consensus sequence of the 15 fibronectin type III (FN3)-binding domains of the human tenascin-C protein.^92^ One study has recently been published on Centyrins that neutralize the bicomponent leukocidins of *S. aureus*.^93^ These Centyrins blocked binding of the bicomponent leukocidins to their host cell surface receptors and thereby also protected human phagocytes from leukocidin-mediated killing. In murine models of leukocidin intoxication, Centyrins and Centyrin–serum albumin fusion constructs pre-mixed with leukocidins before intravenous administration or Centyrins given prophylactically before leukocidin administration protected the mice. Centyrin–serum albumin fusion constructs also markedly improved survival and reduction of bacterial burdens when given 4 h after intravenous infection with highly virulent methicillin-resistant *S. aureus* (MRSA). With further engineering, these biologic agents with toxin-neutralizing activity could have potential in the treatment and prevention of serious staphylococcal infections.

### Receptor Analogs and Neutralizing Scaffolds—Cell Surface Binding

**Figure 6:**
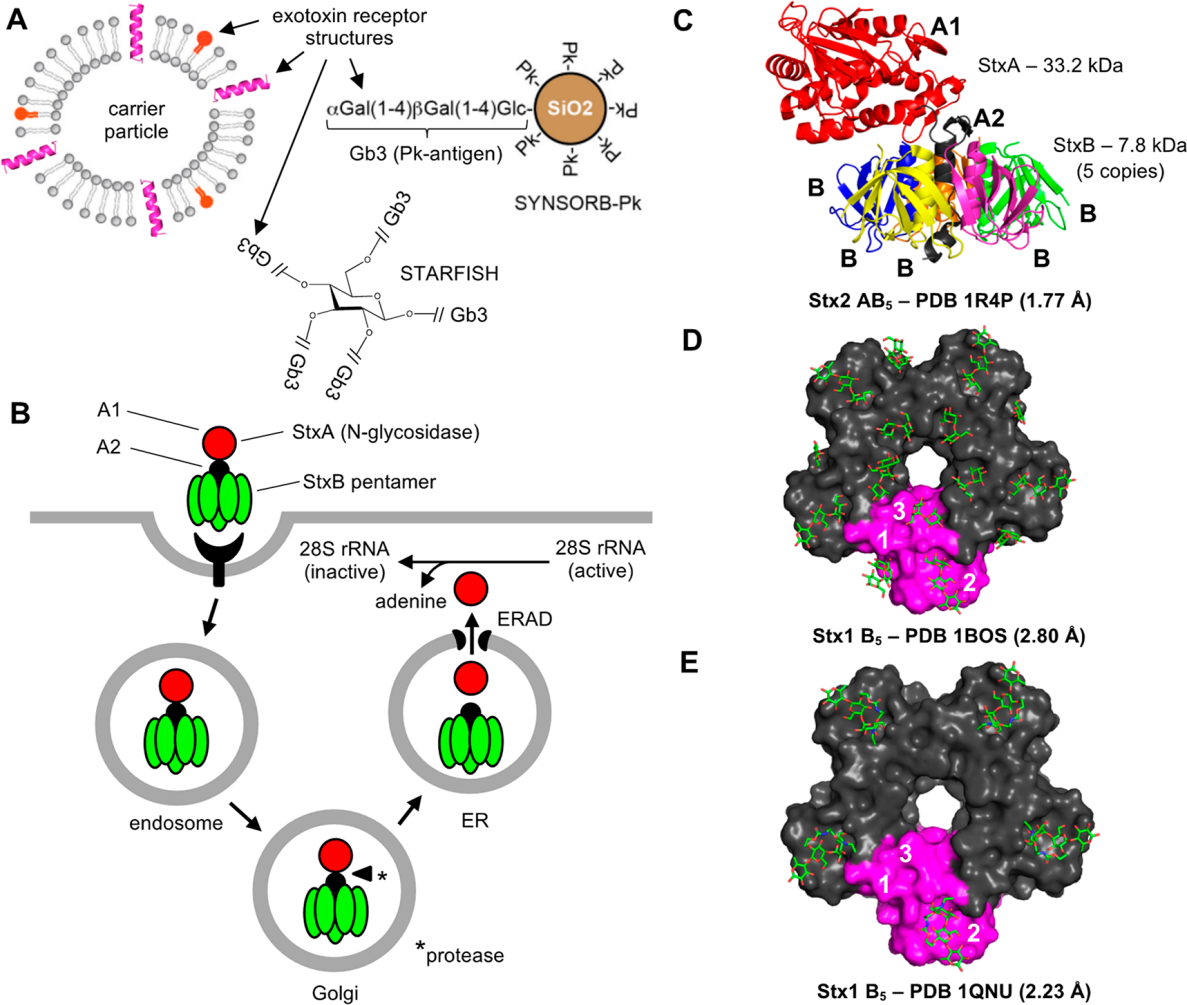
Receptor analogs and neutralizing scaffolds as exotoxin-targeted drugs: schematic example *E. coli* Shiga toxin. (A) Schematic representation of receptor analogs and neutralizing scaffolds. Key advantages and disadvantages of receptor analogs and neutralizing scaffolds as exotoxin-targeted drugs are described in Table 2. (B) Host cell intoxication by Shiga toxins. The StxB pentamer mediates binding of Shiga holotoxin to the host cell surface receptors, which leads to endocytosis. The internalized Shiga toxin undergoes retrograde trafficking to the ER, during which it is proteolytically processed. The liberated A1 domain of StxA gains access to the cytosol via hijacking the ER-associated degradation (ERAD) pathway. In the cytosol, the A1 domain of StxA engages its N-glycosidase activity; i.e., it depurinates the ribosomal 28S rRNA leading to blockage of protein synthesis.^63^ (C) *E. coli* Shiga toxin. X-ray structure of the *E. coli* Shiga toxin 2 (Stx2)^153^ as viewed from the side. The StxA is composed of two domains, A1 and A2, shown in red and black, respectively. The A2 domain inserts into the internal channel of the StxB pentamer. Proteolytic cleavage between the A1 and A2 domains releases the A1 domain for subsequent transport to the cytosol. (D) *E. coli* Shiga toxin in complex with a receptor analog. X-ray structure of the *E. coli* Shiga toxin 1 (Stx1) in complex with a receptor analog^154^ as viewed from the bottom. Each StxB subunit, one highlighted in magenta, has three binding sites for the analog of Shiga toxin receptor globotriaose (Gb3). (E) *E. coli* Shiga toxin 1 (Stx1) in complex with STARFISH. X-ray structure of the *E. coli* Shiga toxin 1 (Stx1) in complex with the polyvalent receptor analog STARFISH^94^ as viewed from the bottom. The Stx1-neutralizing STARFISH binds to the Gb3-binding site 2 of Stx1 and prevents Stx1 from binding to the host cell surface.

Receptor analogs and neutralizing scaffolds is a highly heterogeneous group of exotoxin-targeted drug leads (Table 1, Table S1). They prevent the interaction of exotoxins with their host cell receptor structures, i.e., lipids, carbohydrates, or proteins, via molecular mimicry, or they reduce the bioavailability of the soluble forms of exotoxins via sequestration. Key benefits include generally good tolerability, as many of these are based on natural host cell surface structures (Table 2). These modalities include some of the earliest attempts to develop exotoxin-neutralizing strategies. However, recent interesting developments have emerged, e.g., combinations of multiple modes of action into a single product (Table S1). Three development pipelines have entered clinical trials: Tolevamer, SYNSORB-Pk, and CAL-02 (Table 1). The schematic modality example of receptor analogs and neutralizing scaffolds is the carbohydrate receptor mimicking STARFISH that neutralizes Shiga toxins (Figure 6).

The STARFISH,^94^ Daisy,^95^ and Super Twig^96^ concepts are polyvalent Shiga toxin carbohydrate receptor analogs which have been efficient in pre-clinical *in vitro* and *in vivo* experimentation. However, clinical trial data has not been published on these early drug candidates. An interesting variant concept of receptor analogs, which also acts as an efficient neutralizing scaffold, relies on the use of a recombinant bacterium that expresses a mimic of the Shiga toxin receptor globotriaose (Gb3) on its surface.^97^ This engineered bacterium was also effective *in vivo*, protecting mice from fatal STEC infection.^97^ This concept was recently upgraded via the development of Gb3 receptor mimic bacterial ghosts.^98^ Bacterial ghosts are empty, non-living bacterial envelopes of Gram-negative bacteria that are not classified as genetically modified organisms and thereby could remove barriers in the development of bacterium-displayed Gb3 toward clinical use.^98^

SYNSORB-Pk is a polymer with the Shiga toxin host cell surface receptor globotriaose (Gb3, also known as the Pk-antigen) trisaccharide moiety covalently linked to silicon dioxide particles.^99^ Orally administrated SYNSORB-Pk was safely tolerated by healthy adult volunteers in a Phase I study without any evidence of toxicity.^99^ In the same study, SYNSORB-Pk remained active upon passage through the gastrointestinal tract; i.e., it neutralized Shiga toxin in STEC-positive stool samples from patients with HUS or hemorrhagic colitis.^99^ However, a multicenter, double-blind Phase III clinical trial demonstrated that SYNSORB-Pk was ineffective at reducing the severity of diarrhea-associated HUS in pediatric patients.^100^ There are a number of possibilities to explain the negative outcome, one being simply the lack of efficacy. However, only a third of the enrolled diarrhea-associated HUS patients had viable STEC or free Shiga toxins in their stool.^100^ The authors proposed that the SYNSORB-Pk intervention might have started too late to have a therapeutic effect; i.e., Shiga toxin had already entered the circulation. The SYNSORB-Pk development pipeline has apparently been on hold since the discouraging Phase III trial.

Tolevamer, formerly known as GT160-246 and GT267-004, is a high-molecular-weight (≥400 kDa), soluble linear polymer of styrenesulfonate that binds and neutralizes *C. difficile* toxins TcdA and TcdB *in vitro* and *in vivo*.^101,102^ The exact binding mode is not known. The GT160-246 version was found to be non-inferior to, i.e., not worse than, vancomycin in mild to moderate CDI in a Phase II clinical trial.^103^ The GT160-246 version was well-tolerated in this Phase II trial, but a common side effect was hypokalemia.^103^ Therefore, a new oral solution formulation with a mixed potassium sodium salt of Tolevamer (GT267-004) was developed.^104^ The GT267-004 version demonstrated lower hypokalemia side effects and was well-tolerated in a Phase I trial.^104^ However, the GT267-004 version was found to be inferior to, i.e., worse than, standard antibiotic therapy for CDI conducted with either vancomycin or metronidazole in two multinational Phase III trials.^105^ This discouraging result could, in part, be explained by the fact that Tolevamer interacts less tightly with TcdB as compared to TcdA *in vitro*.^102^ Animal experimentation and the prevalence of TcdA- and TcdB-encoding genes in clinical *C. difficile* isolates also indicate the dominance of TcdB in disease pathology.^23^ The Tolevamer development pipeline has apparently been on hold since the discouraging Phase III clinical trials.

Recently, nanoparticles functionalized with lipids, receptors, receptor fragments, or peptides have been developed as one type of neutralizing scaffolds (Table S1). For example, calcium phosphate nanoparticles loaded with peptides derived from the host cell receptor, which interacts with the conserved cholesterol-binding loop of cholesterol-dependent cytolysins,^106,107^ improved survival and bacterial clearance in *in vivo* models of pneumococcal infection.^106^ Alternatively, by using membrane-mimicking scaffolds, such as nanoparticles coated with lipids, liposomes containing cholesterol at higher than physiological levels,^108^ exosomes,^109^ or so-called biomimetic nanosponges composed of a red blood cell membrane (RBCM) fused to a polymer nanoparticle core, it is possible to inhibit a wide variety of exotoxins from binding to the host cell membrane.^110,111^ One application of the nanosponges is to include an antibiotic^111^ or other bacterium-targeting molecule^112^ into the nanoparticle core. When the exotoxins bind and destroy the RBCM coating, the antibacterial compound trapped inside the nanoparticle is released. Whole red blood cells can also be used as scaffolds to prolong the circulatory half-life of exotoxin-neutralizing molecules. Genetically engineered red blood cells expressing chimeric proteins of camelid VHHs conferred long-term protection against BoNT/A when transfused to mice exposed to lethal doses of BoNT/A.^113^ One of the exciting new approaches relies on the use of liposomes. CAL-02 consists of a mixture of liposomes that create artificially large and stable liquid-ordered lipid microdomains and function as docking sites for a large range of bacterial toxins.^114^ CAL-02 recently entered Phase I trial in severe pneumococcal pneumonia, and it possessed a promising safety profile and tolerability when administered by infusion.^114^

### Dominant-Negative Mutants—Cell Surface Binding

Several exotoxins, in particular membrane-disrupting toxins such as α-toxin of *S. aureus*(115) require assembly and oligomerization in order to execute their cytotoxic effector activities. While deciphering the mechanisms by which leukocidin LukED, the pore-forming exotoxin of *S. aureus*, targets and kills host cells, short glycine-rich motifs within the stem domains of LukE and LukeD were identified as necessary structural elements.^116^ Remarkably, mutant leukocidin subunits lacking these motifs behaved as dominant-negative toxins and neutralized the cytolytic activity of wild-type leukocidins *in vitro* in cell cultures.^116^ The mutant leukocidin subunits appeared to bind on the host cell surface receptors and also were able to interact with the wild-type leucocidin subunits.^116^ The data implies that mechanistically the dominant-negative mutant subunits and wild-type subunits of leukocidins oligomerize but assemble into a defective pore complex, thereby inhibiting toxicity. It is interesting that intravenous administration of dominant-negative mutants had a prophylactic and therapeutic effect in mouse models of intravenous leukocidin challenge and *S. aureus* infection, respectively.^116^

**Figure 7:**
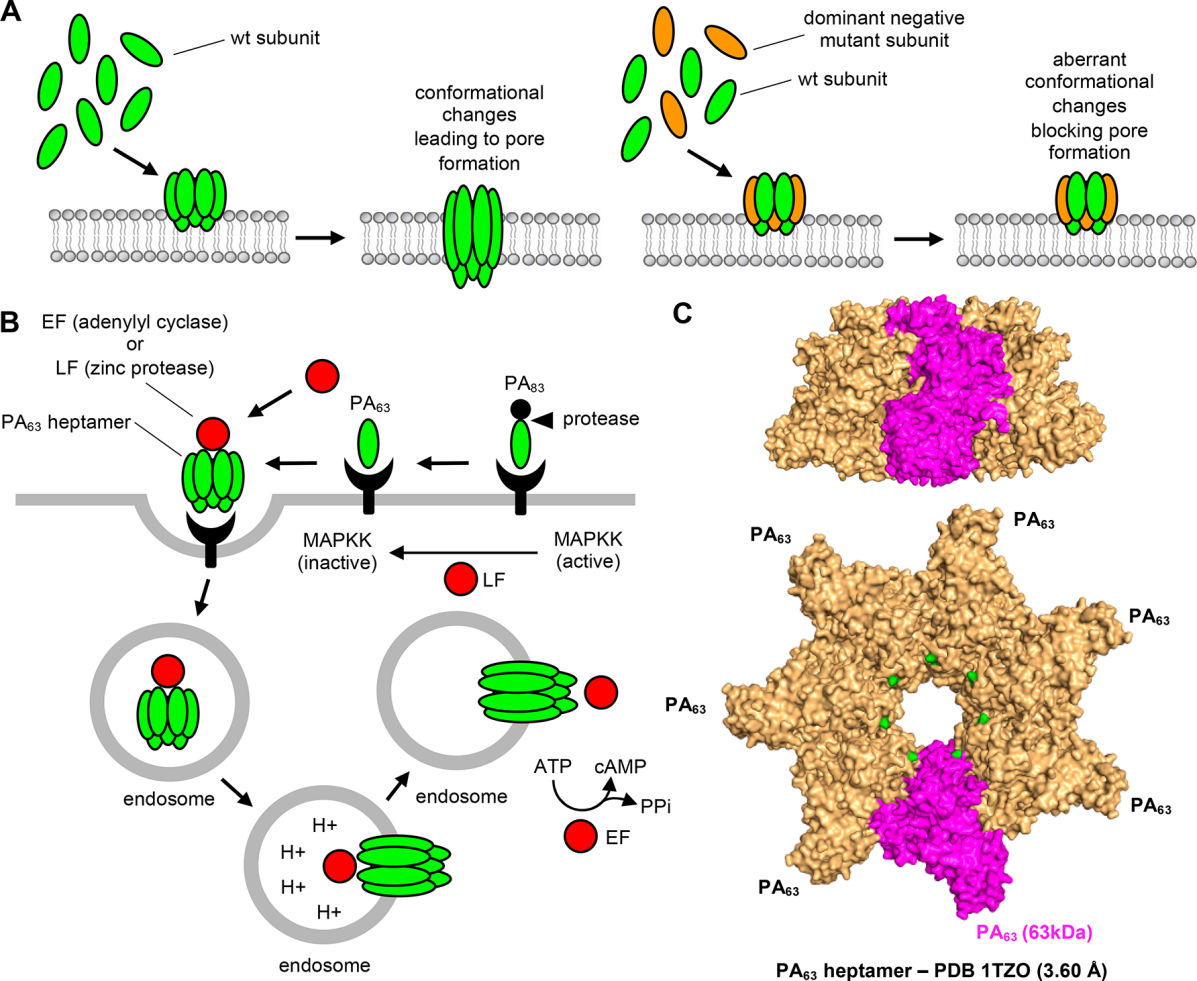
Dominant-negative mutants as exotoxin-targeted drugs: schematic example *Bacillus anthracis* anthrax toxin. (A) Schematic representation of the main principle on the use of dominant-negative mutants as exotoxin-targeted drugs. Many exotoxins, in particular the pore-forming exotoxins, require extensive conformational changes and subunit assembly to oligomeric structures to execute their cytotoxic activities. Mutated forms of some exotoxins, e.g., anthrax toxin, have been identified which get incorporated into the maturing oligomeric structure, but they block the subsequent activatory conformational changes. The end result is a defective pore and prevention of cytotoxicity. Key advantages and disadvantages of dominant-negative mutants as exotoxin-targeted drugs are described in Table 2. (B) Host cell intoxication by anthrax toxin. Anthrax toxin is a tripartite exotoxin; i.e., it is composed of protective antigen (PA) and either lethal factor (LF) or edema factor (EF).^36^ The PA_83_, i.e., full-length 83 kDa form, binds to the host cell surface, where it is proteolytically processed into the PA_63_. The PA_63_ forms oligomers and recruits either the LF or EF. The PA_63_-LF/EF complex is endocytosed. Subsequent acidification of the endosome triggers the pore formation and release of LF/EF into the cytosol. Once in the cytosol, LF inactivates proteolytically mitogen-activated protein kinase kinases (MAPKKs), and EF increases the cytosolic concentration of cyclic AMP (cAMP). (C) Anthrax toxin. The X-ray structure of the anthrax toxin PA_63_ heptamer.^155^ The anthrax toxin PA_63_ heptamer viewed from the side (top) and from the bottom (bottom). One PA_63_ subunit is highlighted in magenta. The D425, highlighted in green, is one example of an amino acid in anthrax toxin PA_63_ where mutations have been identified, e.g., D425 K,^120^ with dominant-negative effects preventing anthrax toxin functions.

The above study on *S. aureus* leukocidins was preceded by other similar studies proposing the use of dominant-negative mutants to prevent the functions of membrane-disrupting toxins (Figure 7A), e.g., on *Clostridium perfringens* ε-toxin,^117^*Helicobacter pylori* VacA,^118^ and *B. anthracis* anthrax toxin.^119,120^ The schematic modality example of dominant-negative mutants is the D425 amino acid-centered dominant-negative forms of protective antigen (PA) that neutralize the anthrax toxin (Figure 7C). These examples imply that the use of dominant-negative mutants is a feasible strategy to neutralize multimeric membrane-disrupting toxins (Table 2). However, efficient and broad development of this drug modality would require an in-depth high-resolution structural knowledge, allowing rational mutant design. Also, the number of mutations that inactivate the toxins is expected to be substantially greater than the number of mutations that lead to a dominant-negative phenotype. In the end, this means more screening work and slower progress. One additional potential problem, based on the recent *S. aureus* leukocidin work,^116^ appears to be the short half-lives. Intravenously administrated dominant-negative mutants were protective if they were given no more than 5 h before the wild-type leukocidin challenge.^116^ For now, it appears that the dominant-negative mutants of exotoxins remain as very useful basic research tools rather than efficient templates for drug development. However, one variant of the dominant-negative approach is the use of exotoxin-derived peptides, which destabilize the exotoxin structure, as exemplified with TcdB of *C. difficile*.^121^ These kinds of peptides are expected to have better pharmacokinetic properties as compared to full-length protein subunits.

### Small Molecules—Cell Surface Binding

Small molecules have been the traditional basis for drug development, and almost two-thirds of approved medicines are either naturally derived or synthetic small molecules.^122^ Small-molecule drugs typically have no more than 100 atoms, and they are no bigger than 1000 g/mol or 1 kDa in size. Small molecules have distinct advantages as exotoxin-targeted drugs (Table 2). Due to their small size, small molecules penetrate tissues efficiently and may also enter the cell, allowing effective targeting of cytosolic processes. Most can be formulated and optimized for oral administration, allowing absorption into the bloodstream and thereby access to the whole body. Due to the possibility to produce small molecules via chemical synthesis, the production costs are typically lower as compared to other modalities, e.g., mAbs. Small molecules can be designed to engage biological targets, mostly proteins, by various modes of action with high-resolution structure-based rational drug design approaches. These include binding to and inhibition of enzyme active sites, binding to allosteric sites influencing enzyme activities and structural transitions, and binding to regions of proteins mediating interactions with other proteins, i.e., protein–protein interaction inhibitors. In addition, high-throughput screening with small-molecule compounds or fragment libraries using cell-based or *in vitro* biochemical assays allows efficient identification of bioactive hit compounds.

Small molecules that prevent the cell binding of exotoxins have been identified using both unbiased high-content screening exercises and high-resolution structure-based rational drug design. One notable study utilized an imaging-based phenotypic screen to identify small molecules that protected the cells from *C. difficile* TcdB-induced morphological alterations.^123^ The screen led to identification of methyl cholate, a bile acid derivative. At the cellular level, methyl cholate lowered the amounts of cell-associated TcdB.^123^ In an *in vitro* biochemical assay, methyl cholate suppressed the IP6-induced auto-processing activity of TcdB. The data indicates that methyl cholate binds to TcdB and induces a conformational change affecting receptor binding and auto-processing activity.

The cytolytic process of the pore-forming toxins of *S. aureus*, α-toxin and bicomponent leukotoxins, begins with the binding of soluble toxin monomers to a cell surface receptor, where they associate to form a non-lytic, oligomeric pre-pore structure.^13^ Finally, the translocation of the pre-stem regions across the membrane results in the bilayer-spanning β-barrel pore structure and consequent membrane permeabilization and cell lysis.^13^ In a recent study, crystal structures revealed evolutionarily conserved phosphatidylcholine-binding mechanisms for LukED, PVL, and α-toxin.^124^ A phosphatidylcholine mimetic compound, *n*-tetradecylphosphocholine (C14PC), significantly reduced the lytic activity of these toxins *in vitro*. In addition to broad-spectrum inhibitory action toward LukED, PVL, and α-toxin, C14PC also has low production costs, and thus it might serve as a starting point in the development of agents that reduce the virulence of *S. aureus* infection prophylactically and therapeutically. The C14PC compound is also expected to be well-tolerated by humans, as the similarly structured drug miltefosine (hexadecylphosphocline, also known as Impavido) is FDA-approved as an oral antiparasitic for the treatment of leishmaniasis.^125^

## Interfering with Intracellular Maturation

Intracellular-targeting toxins such as TcdB of *C. difficile*, BoNTs, and Shiga toxins (Figure 6B) undergo complex maturation processes, often involving complete retrograde trafficking from the endosome to Golgi and ER followed by effector subunit release into the cytosol. Exotoxins may rely on their auto-processing properties, e.g., TcdB of *C. difficile*, or be dependent on oligomerization in order to deliver their enzymatic cargo into the cytosol, e.g., anthrax toxin (Figure 7B). Monoclonal antibodies, antibody fragments, and small molecules have been identified that interfere with these processes.

### Monoclonal Antibodies—Intracellular Maturation

Numerous exotoxin-neutralizing mAbs have been identified (Table 1, Table S1). Depending on the binding epitope, these mAbs may not necessarily prevent exotoxin binding to the host cell surface receptor but act more downstream in the functional pathway of exotoxins. The downstream effect is exemplified in the case of the developmental pipeline with humanized mAbs PA-50 and PA-41 targeting *C. difficile* TcdA and TcdB, respectively.^126^ The humanized mAbs PA-50 and PA-41 efficiently neutralized TcdA/TcdB in cell culture experiments and demonstrated efficacy in a hamster model for CDI.^126^ The PA50 mAb binds to multiple sites on the TcdA C-terminal CROPS domain.^127^ Binding of TcdA to the host cell surface was prevented by PA50 mAb, indicating that receptor blockade is the mode of action by which PA50 neutralizes TcdA.^127^ This is the same mode of action by which the clinically used anti-TcdB mAb bezlotoxumab works^25−28^ (Figure 3C). In contrast, an entirely different neutralization mechanism was found for PA41, the TcdB-specific mAb.^128^ The PA41 mAb recognizes a single, highly conserved epitope on the TcdB glucosyltransferase domain.^128^ The PA41 mAb does not block TcdB from binding or entering the host cell via endocytosis.^128^ The PA41 mAb rather prevents the translocation of the glucosyltransferase enzymatic cargo from the endosome into the host cell cytosol^128^ (Figure 3B).

Alternative modes of action have also been reported for anthrax toxin-neutralizing mAbs. Following endocytosis of the pre-pore-EF/LF complex, an acid-driven pre-pore-to-pore conversion occurs, thus promoting the entry of EF/LF into the cytosol, where they exert their toxic effects^129^ (Figure 7B). The cAb29, an anti-PA antibody, appeared to prevent the PA-formed pre-pore from undergoing conformational changes into the mature pore structure in the acidic endosomal compartment.^129^ This mode of action is in contrast to those of the FDA-approved obiltoxaximab and raxibacumab, which recognize the receptor-binding region of PA^40,41^ and thereby block PA–host cell surface interactions. Moreover, intracellular maturation-blocking mAbs have been identified in the Shiga toxin-focused drug development efforts, e.g., also in the Shigamabs developmental pipeline. For example, human mAb 5C12, which binds to the catalytic A-subunit, did not interfere with the cell surface binding of Stx-2.^130^ In contrast, 5C12 blocked the retrograde transport of Stx-2 into the Golgi and ER, preventing the entry of the A-subunit into the cytosol.^130^ The 5C12 study demonstrates an important point with respect of the use of exotoxin-neutralizing mAbs. The 5C12 was able to bind to the already cell-bound Stx-2.^130^ This potentially extends the therapeutic window as compared to mAbs, which merely prevent the binding of exotoxins to their respective host cell surface receptors.

### Small Molecules—Intracellular Maturation

Interesting development pipelines have been focused on small molecules that interfere with the intracellular maturation of exotoxins, in particular their auto-processing activity. Ebselen (2-phenyl-1,2-benzoselenazol-3-one) is a lipid-soluble membrane-penetrating organoselenium compound.^131^ Ebselen has generic antioxidant properties; e.g., it catalyzes the reduction of reactive oxygen species in a manner similar to glutathione peroxidase.^131^ Ebselen also covalently modifies cysteine residues.^131^ Ebselen was identified as an inhibitor of the auto-processing cysteine protease domain (CPD) of TcdB in an *in vitro* fluorescence polarization high-throughput screen.^132^ Follow-up studies demonstrated that Ebselen also inhibited auto-processing of TcdA.^132^ Mechanistically, Ebselen covalently modified the CPD domain of TcdA/TdB at cysteine residues, leading to suppression of cysteine protease activity.^132^ Ebselen was also identified independently as a TcdB inhibitor in a high-throughput cell phenotypic screen.^123^ These authors proposed that Ebselen acts on the glycosyltransferase activity of TcdB, preventing glycosylation of the small GTPase Rac1.^133^ The inhibitory action on TcdB appeared to be indirect, acting via Ebselen-mediated modification of cysteine residues on Rac1.^133^ The initial screening studies showed that Ebselen protected cells and mice against TcdA/TcdB-mediated killing and improved histopathology in a murine CDI model.^123,132^ Recently, animal experimentation was extended to show that Ebselen, as a monotherapy, reduces recurrence rates and decreases the severity of colitis in animal models of CDI.^134^ Moreover, Ebselen has already advanced to Phase III clinical trials in diseases unrelated to CDI, e.g., diabetes (NCT00762671). As for now, it remains unknown to what extent Ebselen functions via its generic anti-inflammatory properties and to what extent via its anti-TcdA/TcdB functions. Pan-reactivity with cysteine residues is a concerning fact, but the exotoxin neutralization potency itself, not the detailed mechanism of action, is perhaps of more practical interest.

**Figure 8:**
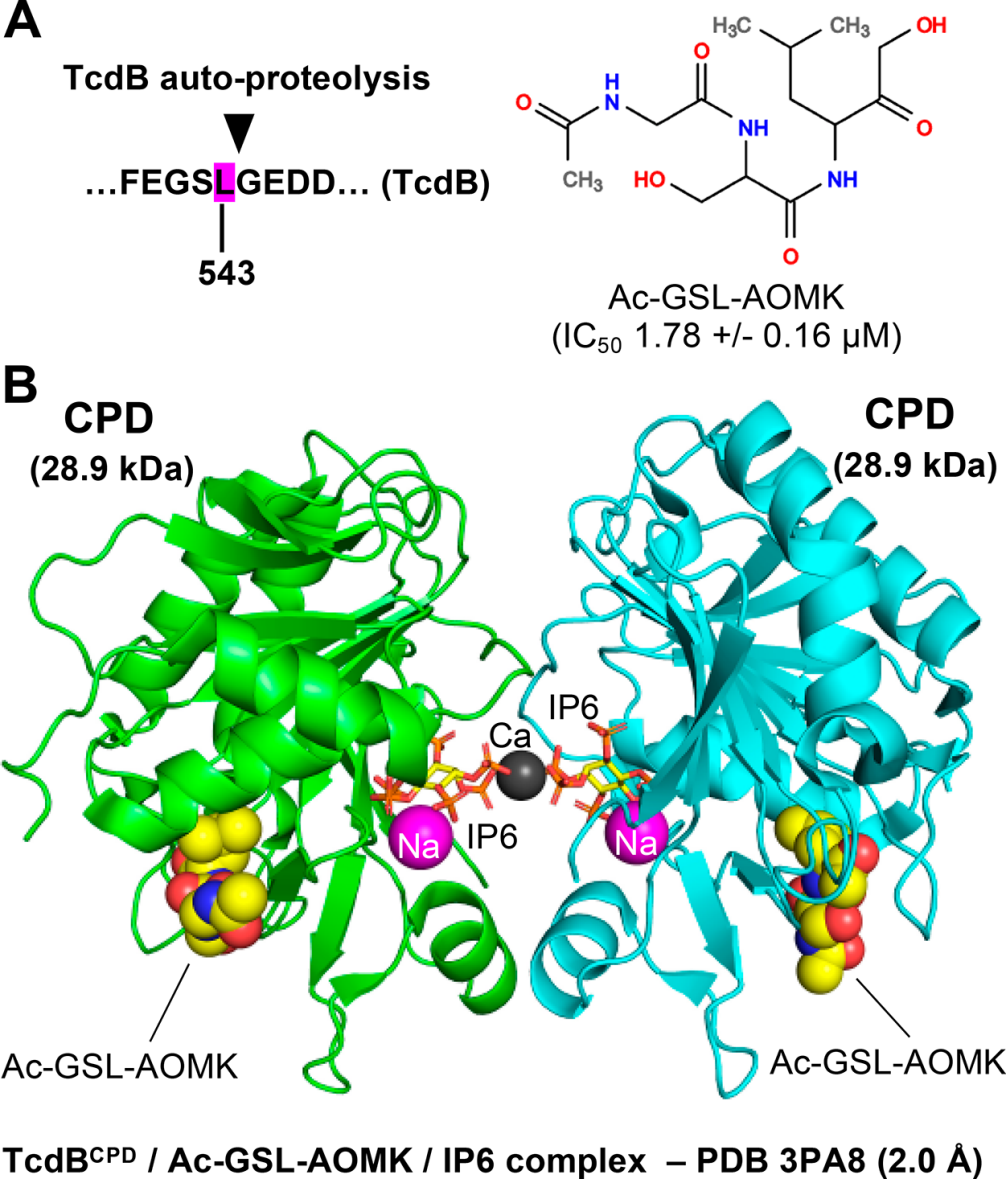
Small molecules as exotoxin-targeted drugs: schematic example *C. difficile* TcdB. Small molecules have been the traditional basis for drug development, although to a lesser extent yet with exotoxins. Key advantages and disadvantages of small molecules as exotoxin-targeted drugs are described in Table 2. (A) A small molecular weight compound inhibiting the auto-proteolytic activation of TcdB of *C. difficile*. The auto-proteolytic activation of TcdB is a centrally important mechanism of host cell intoxication by TcdB (see Figure 3B). This activity is mediated by the cysteine protease domain (CPD) of TcdB upon activation by the cytosolic hexakisphosphate (IP6). A series of CPD inhibitors have been identified, e.g., Ac-GSL-AOMK.^136^ (B) A TcdB^CPD^ in complex with Ac-GSL-AOMK. The X-ray structure of TcdB^CPD^ in complex with Ac-GSL-AOMK.^136^ Two CPD molecules are shown sandwiched via a complex formed by one calcium ion, two sodium ions, and two IP6 molecules. The structure demonstrates the binding of Ac-GSL-AOMK to the active site of CPD, which is on the other side of the IP6 binding site of CPD.

The multifunctional auto-processing repeats-in-toxins (MARTX) toxin, e.g., in *V. cholerae*, also relies on proteolytic auto-processing for cellular activity.^135^ Similar to the CPD domains of clostridial TcdA and TcdB, the MARTX toxin of *V. cholerae* is activated by IP6.^135^ Covalent cysteine protease inhibitors were identified which interfered with the MARTX toxin auto-processing.^135^ Notably, a high-resolution structure of CPD in complex with the aza-leucine epoxide inhibitor JCP598 was determined.^135^ The overall structure is nearly identical to the activated CPD, with the inhibitor docking into the active-site cleft created upon binding of IP6 to the CPD.^135^ A similar kind of a study has been published on covalent *C. difficile* CPD inhibitors,^136^ building in part on the work on *V. cholerae* MARTX toxin.^135^ High-resolution structural information was obtained of the inhibitor–CPD complex, and some of the analyzed small molecules were potent in living cells to inhibit TcdB functions.^136^ It remains to be determined if the specificity of these particular covalent protease inhibitors for MARTX and TcdA/TcdB toxins is high enough at the cellular and whole-body levels to allow their further development as drug leads. The schematic modality example of small molecules is the Ac-GSL-AOMK compound neutralizing the TcdB of *C. difficile* (Figure 8).

A novel therapeutic paradigm explored the possibility to target the auto-proteolysis activity of TcdB by triggering its IP6-induced auto-proteolysis in the gut lumen.^137^ To reach this goal, gain-of-function small molecules, IP6 analogues, were synthesized by progressively replacing the IP6 phosphate groups with sulfate groups. This was done in order to reduce the susceptibility of IP6 to complexation at physiological calcium concentrations at the colon lumen while maintaining the uniquely high charge density that mediates its interaction with TcdB. Partial replacement of phosphates by sulfates and thiophosphates resulted in analogs (IP2S4, IT2S4) capable of inducing TcdB cleavage at micromolar concentrations in the presence of calcium.^137^ In a mouse model of colitis, oral administration of IP2S4 attenuated the symptoms. Furthermore, treatment with the thiophosphate analog IT2S4, which has improved stability toward inositol phosphatase enzymes that may be present in the gut lumen, rescued mice in the acute CDI model.^137^

## Interfering with Cytosolic Effector Functions

This step in the functional pathway of exotoxins refers to the point where the exotoxin, in particular its effector domain, has been released from the endosome or the Golgi/ER compartment into the cytosol (Figures 2, 3B, 4B, 6B, and 7B). Some exotoxins also gain access into the cytosol straight from the plasma membrane. For instance, NAD^+^ glycohydrolase (SPN) of *Streptococcus pyogenes* utilizes the multimeric pore structure created by another exotoxin of *S. pyogenes*, streptolysin S (SLO), at the host cell membrane.^138^ Also, the bifunctional hemolysin/adenylyl cyclase (CyaA) of *B. pertussis* first binds to the surface and subsequently inserts its cyclic AMP (cAMP)-generating catalytic domain into the cytosolic side of the plasma membrane.^139^ For now, the developmental pipelines have focused exclusively on small molecules to interfere with the cytosolic effector functions.

### Small Molecules—Cytosolic Effector Functions

There have been a number of attempts to develop small molecules inhibiting the cytosolic effector functions of exotoxins. The major advantage with these compounds would be that they are capable of preventing exotoxin functions after the exotoxin has been internalized. This mode of action should open up wider practical possibilities, in particular in therapeutic use. One notable high-content screening exercise was undertaken to identify inhibitors of the glucosyltransferase activity of *C. difficile* TcdB^140^ (see Figure 3B). The compounds were screened utilizing a 1536-well fluorescence polarization assay for UDP-glucose hydrolysis activity by the C-terminal glucosyltransferase domain of TcdB.^140^ Multiple hits were identified from a diverse six-million-member compound collection.^140^ Hit-to-lead optimization efforts centered around a novel series of benzodiazepinedione-based inhibitors.^140,141^ Optimized compounds demonstrated good pharmacokinetic profiles in mouse and hamster and were efficacious in multiple cell culture and animal models of CDI upon oral dosing.^140,142^ We have recently identified small molecules inhibiting the ADP-ribosyltransferase activity of pertussis toxin.^143^ We developed an *in vitro* high-throughput-compatible assay to quantify NAD^+^ consumption during PtxS1-catalyzed ADP-ribosylation of Gαi *in vitro*. Two inhibitory compounds, NSC228155 and NSC29193, with low micromolar IC_50_ values were identified in the *in vitro* NAD^+^ consumption assay via screening of a focused compound library. These compounds were also potent in an independent *in vitro* assay monitoring conjugation of ADP-ribose to Gαi. Moreover, the membrane-permeable NSC228155 inhibited the pertussis AB5 holotoxin-catalyzed ADP-ribosylation of Gαi in living human cells with a low micromolar IC_50_ value (2.4 μM). We currently employ medicinal chemistry efforts, including molecular modeling and protein crystallography, in an attempt to design NCS228155 analogs with additionally increased potency and specificity.

In addition to *B. pertussis*, ADP-ribosyltransferases are key virulence factors of several pathogens such as *C. diphtheria* (diphtheria toxin), *V. cholera* (cholera toxin), and *E. coli* (heat-labile enterotoxin).^144^ Selective targeting and inhibition of the ADP-ribosyltransferase activity holds promise to interfere with disease pathology. Compounds inhibiting *P. aeruginosa* ExoA-induced cytotoxicity in yeast and mammalian cell-based assays *in vitro* have been identified.^145^ Virtual screening on the crystal structure of a closely related cholic toxin of *V. cholera* was primarily used to design the screened compound library.^146^ Hit compounds for ADP-ribosyltransferases of *B. sphaericus*, *C. difficile*, and *C. botulinum* were found via *in vitro* screening of kinase inhibitors, which are typically adenosine mimics and thereby chemically related to NAD^+^.^147^ Bisubstrate analogs mimicking the nicotinamide portion of NAD^+^ and arginine residue of the target host cell protein have also been developed to inhibit cholera toxin.^148^ In addition, structures of NAD^+^- or hit compound-bound ADP-ribosyltransferases have allowed computational analyses to understand the binding modes and to provide rational ideas for further improvements, as in the case of cholix toxin of *V. cholera*.^145,149^

Small molecules that prevent the cytosolic effector functions have also been identified by cell-based screening exercises. The naturally occurring flavonoid phloretin was identified as a compound protecting cells from both *C. difficile* TcdA- and TcdB-induced cell rounding.^123^ Subsequent validation experiments demonstrated that phloretin was a direct inhibitor of the toxin GTD domains of both TcdA and TcdB.^123^ The authors conducted a secondary focused library screening with flavonoid compounds and identified two potent analogs of phloretin.^123^ Phloretin appears to act as a non-competitive inhibitor and thereby with a probable allosteric action. The authors argued that this mode of action may offer high selectivity and specificity over other enzymes that utilize the same substrate, in this case UDP-glucose.^123^ This highlights the drawback, for example, in our own ADP-ribosyltransferase studies where we aimed to identify competitive small molecules binding to the NAD^+^-binding active site of pertussis toxin.^143^ These compounds may also interact with the plethora of other NAD^+^-binding proteins in the cell, such as members of the poly(ADP-ribose)-polymerase (PARP) protein family.^150^ It remains to be studied whether these off-target effects are a concern.

## Conclusions and Future Perspectives

What constitutes a good exotoxin target for drug development efforts? First of all, a good exotoxin target has a significant or preferably primary role as the disease-causing virulence factor. This is indeed the case in many globally significant infectious diseases, e.g., whooping cough, cholera, diphtheria, tetanus, botulism, anthrax, and toxic shock syndrome. In the case of exotoxin redundancy in virulence, cocktails of different exotoxin-targeting drugs could be developed, although this would increase the developmental costs and the lengths of the developmental pipelines. Second, a good exotoxin target should provide a broad enough therapeutic window for interference. Typically, upon clinical suspicion of a bacterial infection, patients receive empirical antibiotic therapy, in many cases broad-spectrum, before the diagnostic data becomes available. The exotoxin-targeted drugs are pathogen-specific and thereby require a diagnostic finding to be effective. When such data becomes available, can we still interfere with the disease pathology with or without antibiotics? The answer to this question appears to be “no” in some acute and severe infectious diseases, such as toxic shock syndrome, where superantigens play a dominant role. However, more slowly progressing and/or relapsing infectious diseases, such as the *C. difficile* infection, allow interference with disease pathology via exotoxin neutralization. Also, a broad enough therapeutic window is expected in cases where exotoxins stay active well after the invading bacterium has been killed by antibiotics or the immune system, e.g., in the case of anthrax toxin. In addition, some infectious diseases are linked to more severe outcomes if treated with antibiotics, such as the Shiga toxin-producing *E. coli* infection. Treatment of these diseases would benefit from replacement of antibiotics with alternative therapeutics upon confirmed diagnosis. Third, a good exotoxin target should allow the development of various exotoxin-targeted drug modalities, which each have their specific advantages and disadvantages (Table 2).

The pre-clinical, clinical trial, and real-world clinical use data demonstrate that exotoxin-targeted drugs can be effective, notably exemplified by the toxin B (TcdB)-neutralizing bezlotoxumab to prophylactically reduce recurrence of *C. difficile* infections. Exotoxin-targeted drugs also have pre-clinically proven efficacy as therapeutic pharmaceuticals. Exotoxin-targeted drugs may complement the use of antibiotics, e.g., to allow lowering of the dosage of antibiotics, or they may be used as stand-alone pharmaceuticals. Three main reasons are driving the rapid expansion of research on exotoxin-targeted drugs. First of all, widespread antibiotic resistance calls for the development of new, alternative ways to treat bacterial infections. Second, awareness of the physiological importance of microbiota forces us to consider treatment of bacterial infections with more focused pathogen-specific pharmaceuticals. Third, decades of basic research using various *in vitro* assays, cell and tissue culture models, and animal experimentation have provided an in-depth view on the functions of bacterial exotoxins in bacterial virulence, allowing rational drug design approaches. Taken together, although important progress has been made in the development of exotoxin-targeted drug modalities, and antivirulence therapy in general, significant work is still required to realize the potential of these pharmaceuticals.

## Supporting Information Available

Table S1. Exotoxin-targeted drug leads that are in pre-clinical development. Information in this table is composed of primary research articles published on exotoxin-targeted drug modalities in the past 5 years. The mode of action at the molecular level is not known for some of the leads. Classification is based on the six exotoxin-targeted drug modalities, as specified in the main text. Antibodies and antibody fragments are combined in this table, because some leads are composed of both of these modalities. Moreover, mAb engineering frequently utilizes antibody fragment step, and subsequent linking of the most efficient fragments with Fc-region to engage effector functions of entire mAbs. (PDF)

## Notes

The manuscript does not directly relate to sustainability.

## Abbreviations

28s rRNA: 28s ribosomal RNA
60S: eukaryotic large ribosomal subunit
ADP: adenosine diphosphate
ATP: adenosine triphosphate
AVA: anthrax vaccine absorbed
BAL: bronchoalveolar lavage
BoNT/A–G: botulinum neurotoxin types A–G
C14PC: tetradecylphosphocholine
cAMP: cyclic adenosine monophosphate
C-cap: C-terminal capping repeat
CDI: *Clostridioides difficile* infection
CDT: cytolethal distending toxin
CNF1: cytotoxic necrotizing factor 1
CPD: cysteine protease domain
CROPS: combined repetitive oligopeptides
C-terminal: carboxy-terminal
CyaA: bifunctional hemolysin/adenylyl cyclase
DARPin: designed ankyrin repeat protein
EF: edema factor
ER: endoplasmic reticulum
ERAD: ER-associated degradation
ET: edema toxin
ETEC: enterotoxigenic *Escherichia coli*
ExoA: exotoxin A
F(ab′)_2_: divalent fragment antigen-binding
Fab: fragment antigen-binding
Fc: fragment crystallizable
FDA: U.S. Food and Drug Administration
FN3: fibronectin type III
Gb3: globotriaose
GPCR: G protein-coupled receptors
GTD: glucosyl transferase domain
GTPase: guanosine triphosphatase
Gαi: G-protein alpha subunit, inhibitory
Gαo: G protein alpha subunit, olfactory
Gαt: G-protein alpha subunit, transducin
Hla: alpha toxin
HlgAB/CB: gamma hemolysins AB and CB
HUS: hemolytic uremic syndrome
IC_50_: half-maximal inhibitory concentration
IgG: immunoglobulin G
IP6: hexakisphosphate
kDa: kilodalton
LF: lethal factor
LT: lethal toxin
LukED/AB: leukotoxin ED/AB
mAb: monoclonal antibody
MAPKK: mitogen-activated protein kinase-kinase
MARTX: multifunctional auto-processing repeats in toxin
MHC: major histocompatibility complex
MHCII: major histocompatibility complex class II
MRSA: methicillin-resistant *Staphylococcus aureus*
NAD^+^: nicotinamide adenine dinucleotide
N-cap: N-terminal capping repeat
N-terminal: amino-terminal
PA: protective antigen
PA_63_: proteolytically processed 63 kDa form of protective antigen
PA_83_: full-length 83 kDa form of protective antigen
Pi: inorganic phosphate
PTX: pertussis toxin
PtxS1–S5: pertussis toxin subunits 1–5
PVL: Panton–Valentine leukocidin
Rac1: Ras-related C3 botulinum toxin substrate 1
RBCM: red blood cell membrane
RBD: receptor binding domain
scFv: single-chain fragment variable
SLS: streptolysin S
SPN: *Streptococcus pyogenes* NAD^+^ glycohydrolase
STEC: Shiga toxin-producing *Escherichia coli*
Stx: *Shigella dysenteriae* Shiga toxin
Stx1,2: *Escherichia coli* Shiga toxins 1 and 2
StxB: Shiga toxin B-subunit
TcdA: *Clostridioides difficile* toxin A
TcdB: *Clostridioides difficile* toxin B
TCR: T-cell receptor
TD: translocation domain
TNT: tuberculosis necrotizing factor
TSST-1: toxic shock syndrome toxin-1
TT: tetanus toxin
UDP: uridine diphosphate
VacA: vacuolating cytotoxin A
VAMP: vesicle-associated membrane protein
VHH: variable heavy homodimer
VNA: VHH-based neutralizing agent
