# Supplementary material for: Exotoxin-Targeted Drug Modalities as Antibiotic Alternatives
Source: ACS Infect Dis. 2022 Jan 31;8(3):433–56. doi: 10.1021/acsinfecdis.1c00296 (PMC8922280; doi:10.1021/acsinfecdis.1c00296)
Supplement: Supplementary file 1 — id1c00296_si_001.pdf [file id1c00296_si_001.pdf]

Supporting Information

Table S1. Exotoxin-targeted drug leads that are in pre-clinical development.

Exotoxin-targeted drug modalities as antibiotic alternatives

Moona Sakari[a], Arttu Laisi[a], & Arto T. Pulliainen[a*]

[a]Institute of Biomedicine, Research Unit for Infection and Immunity, University of Turku, Turku, Kiinamyllynkatu 10, FI-20520, Turku, Finland

[*]Corresponding author

Corresponding author information:
Dr. Arto Pulliainen, Ph.D.
Institute of Biomedicine, Research Unit for Infection and Immunity, University of Turku, Kiinamyllynkatu 10, FI-20520, Turku, Finland
Phone: +358-40-1586044, Fax: not available, E-mail: arto.pulliainen@utu.fi
https://orcid.org/0000-0002-9361-8963

Table S1. Exotoxin-targeted drug leads that are in pre-clinical development.[a]

**Monoclonal antibodies and antibody fragments**				
**Lead**	**Pathogen**	**Target**	**Efficacy**	**Ref.**
human IgG (LXY8)	*S. aureus*	staphylococcal enterotoxin B (SEB)	animals	1
human IgG (M0313)	*S. aureus*	SEB	animals	2
human Fab and IgG (Fab-GC132a, IgG-GC132a)	*S. aureus*	SEB	animals	3
human and chimeric IgGs	*S. aureus*	SEB	animals	4
mouse IgG (3E2), engineered pH-dependent recycling antibodies (L2, L6 and L6.R)	*S. aureus*	SEB	animals	5
human-mouse chimeric IgGs	*S. aureus*	SEB	animals	6
human IgG	*S. aureus*	α-toxin	animals	7
bispecific Ab (anti-ClfA 11H10 or SAR114 and an anti-α-toxin, MEDI4893)	*S. aureus*	α-toxin	animals	8
mouse IgGs (MAb-4G3, mAb-5G2, mAb-9H2)	*S. aureus*	staphylococcal enterotoxin (SEK)	animals	9, 10
G03-52-01 [combination of NTM-1631 (XOMA 3Ab) and NTM-1632]	*C. botulinum*	BoNT/A, BoNT/B	animals	11
human IgG	*C. botulinum*	BoNT/B	animals	12
mouse IgG	*C. botulinum*	BoNT/A	animals	13
NTM-1631 (XOMA 3Ab) and NTM-1632	*C. botulinum*	BoNT/A, BoNT/B	animals	14
tri-epitopic human/humanized IgG1 - based mAb (TeAb - IgGs 2G11, CR2, RAZ1)	*C. botulinum*	BoNT/A	animals	15
mouse IgGs (6F11, 6F12 and 6F13)	*C. botulinum*	BoNT/F	animals	16
humanized IgG (hu8ELC18)	*C. botulinum*	BoNT/E	animals	17
human IgGs (RAZ1, CR2, 4E17.2D)	*C. botulinum*	BonT/H	animals	18
humanized IgGs (hu8SEM120-IIIC1, hu8A1HC38, hu8BLC3 and hu8B2-7)	*C. botulinum*	BoNT/A, BoNT/B	animals	19

Camelid VHHs (B11 and G3) as dimers or fused to human IgG1 Fc fragments (VHH-Fcs, fusionbodies)	*C. botulinum*	BoNT/A	animals	[20]
bifunctional camelid VHH heterodimers	*C. botulinum*	BoNT/A, BoNT/B1	animals	[21]
camelid VHH antibody	*C. botulinum*	BoNT/A1	cells	[22]
monomer and heterodimer camelid VHH antibodies (JLE-E5, JLE-E9)	*C. botulinum*	BoNT/E	cells, animals	[23, 24]
camelid VHH	*C. botulinum*	BoNT/E	animals	[25]
mouse IgG	*S. aureus*	γ-Hemolysin C (HlgCB)	cells	[26]
mouse IgGs (2C9B6, 1F2C2, 1F3E3) and their Fab and $F(ab')_2$ fragments	*C. tetani*	tetanus toxin	animals	[27]
human IgGs (BUT-TT-243-10, BUT-TT-143-10, BUT-TT-120-10)	*C. tetani*	tetanus toxin	animals	[28]
human IgG (2-7G, 2-2D, and S-4-7H)	*C. tetani*	tetanus toxin	cells	[29]
mouse and human IgGs	*C. tetani*	tetanus toxin	in vitro	[30]
mouse IgGs (3A9, 6E5, 12F11, 12D10)	*S. pneumoniae*	pneumolysin	cells	[31]
mouse IgG	*B. cereus*	hemolysin II	animals	[32]
human IgGs (SAN177 and SAN481)	*S. aureus*	LukSF-PV (PVL), LukED, HlgAB, HlgCB	animals	[33]
human IgG (YG8-1, YG8-2, YG8-3)	*S. aureus*	LukSF, LukED, HlgAB, HlgCB	cells, animals	[34]
human IgG (mAb#5.H1H2)	*S. aureus*	LukGH	cells	[35]
human IgGs (SA-13, -15 and 17)	*S. aureus*	LukAB	animals	[36]
human scFvs	*S. aureus*	toxic shock syndrome toxin (TSST-1)	cells	[37]
human IgG and scFv-Fc	*C. diphtheriae*	diphtheria toxin	animals	[38]
mouse IgGs (mAb 2-25, mAb 2-18)	*C. diphtheriae*	diphtheria toxin	in vitro	[39]
human scFv (3C, 10S, and 12S)	*C. diphtheriae*	diphtheria toxin	cells	[40]
human IgG (7B1)	*B. anthracis*	protective antigen (PA) of anthrax toxin	animals	[41]
human IgG (PA21)	*B. anthracis*	PA of anthrax toxin	animals	[42]
human IgG (PA21), chimeric IgG (hmPA6)	*B. anthracis*	PA of anthrax toxin	animals	[43]

camelid VHH	*B. anthracis*	PA of anthrax toxin	in vitro	[44]
adenoviral vector promoting bispesific VHH-based neutralizing agent (VNA) consisting of two linked VHHs	*B. anthracis*	PA of anthrax toxin	animals	[45]
humanized IgG (5E11)	*B. anthracis*	lethal toxin (LT)- variant of anthrax toxin	animals	[46]
non-glycosylated human IgG incorporated into PEG hydrogel	*B. anthracis*	LT- variant of anthrax toxin	cells	[47]
synthetic Fabs (A4 and B7) and F(ab)2s	*B. anthracis*	edema toxin (ET)- variant of anthrax toxin	cells	[48]
camelid VHHs and bispesific VNA (JMN-D10 and JMO-G1)	*B. anthracis*	lethal factor (LF) and edema factor (EF) of anthrax toxin	animals	[49]
humanized IgGs (hu11E6 and hu1B7)	*B. pertussis*	pertussis toxin	cells, animals	[50-53]
human IgGs (B1-B25, A1-A13)	*C. difficile*	TcdA, TcdB	cells, animals	[54, 55]
humanized IgG (PA50)	*C. difficile*	TcdA	animals	[56, 57]
humanized IgGs (CANmAbA4, CANmAbB4)	*C. difficile*	TcdA, TcdB	animals	[58]
humanized IgG (PA41)	*C.difficile*	TcdB	animals	[57, 59]
camelid VHH	*C. difficile*	TcdA	cells	[60]
bivalent VHH-Fc fusion (camelid VHHs fused with human IgG1 Fcs)	*C. difficile*	TcdB	in vitro	[61]
tetraspesific camelid VHH-based neutralizing agent (VNA) (VNA2-Tcd) (VHHs AH3, AA6, 5D, E3)	*C. difficile*	TcdA, TcdB	animals	[62]
camelid VHHs (E3, 7F, 5D)	*C. difficile*	TcdB	in vitro	[63 64]
human scFv-Fcs	*C. difficile*	TcdB	cells	[65]
rabbit IgG(JL008)	*C. perfringens*	epsilon toxin (ETX)	animals	[66]
mouse IgGs (21RA, 46RA, 47RA, 50RA)	*V. vulnificus* *V. cholera*	RtxA, MARTXVc	animals	[67]
mouse IgGs (1E23, 2G7 and 2H7)	*C. perfringens*	beta 2 toxin (CPB2)	cells	[68]
mouse IgG (HS1)	*S. pyogenes*	streptolysin O	animals	[69]
human IgG	*S. tyhphi,* *S. paratyphi*	typhoid toxin	animals	[70]
mouse IgGs	*C. perfringens*	alpha toxin (CPA)	cells	[71]
human scFv	*C. perfringens*	CPA	animals	[72]

mouse IgGs (1D9, 2H9)	*B. fragilis*	zinc metalloprotease toxins (BFT1, BFT2)	cells	[73]
mouse IgGs (mAbs 1G4 and 2G11)	*B. cereus*	non-hemolytic enterotoxin (Nhe)	cells	[74]
semisynthetic scFvs	*H. pylori*	vacuolating cytotoxin A (VacA)	in vitro	[75]
mouse IgGs	*E. cloacae*	Stx1e	cells	[76]
camelid VHH (monovalent and bivalent Nb113)	*E. coli*	Stx2a	in vitro	[77]
human recombinant Fab fragment (FabC11:Stx2)	*E. coli*	Stx2	animals	[78]
camelid VHHs and VNA, a trivalent molecule with two copies of anti-Stx2B VHH and one anti-seroalbumin VHH	*E. coli*	Stx2	animals	[79]
humanized camelid VH/VHH nanobodies	*B. pertussis*	bifunctional hemolysin-adenylyl cyclase (CyaA)	in vitro	[80]
human single domain Abs (M1H5, M2B10)	*B. pertussis*	CyaA	cells	[81, 82]
human single-chain antibodies (HuscFv-C41, HuscFV-E44, HuscFv-P32)	*P. aeruginosa*	exotoxin A (ExoA)	cells	[83]
semisynthetic human scFv	*P. aeruginosa*	ExoA	in vitro	[84]
**Antibody mimetics**				
**Lead**	**Pathogen**	**Target**	**Efficacy**	**Ref.**
centyrins and albumin-binding consensus domain-centyrin fusion proteins (ABDcon-centyrins)	*S. aureus*	panton-valentine leukocidin (PVL), HlgAB, HlgCB, LukED, and LukAB	animals	[85]
monomeric and dimeric designed ankyrin repeat proteins (DARPins)	*C. difficile*	TcdB	animals	[86, 87]
mini-protein (Bot.671.2)	*C. botulinum*	BoNT/B	cells	[88]
**Receptor analogs and neutralizing scaffolds**				

Lead	Pathogen	Target	Efficacy	Ref.
polymyxin B-modified RBC-mimetic hybrid liposome	*E. coli*	hemolysin (HlyA), Stx1	animals	[89]
acylhydrazone polymer	*E. coli*	heat-labile enterotoxin	in vitro	[90]
fucose-galactose polymer	*V. cholera*	cholera toxin	cells	[91, 92]
fucosylated meta-nitrophenyl α-galactoside hybrid glycopolymer	*V. cholera*	cholera toxin	in vitro	[93]
GM1a gangliosides in polyethylene glycol (PEG)-based microgel	*V. cholera*	cholera toxin	cells	[94]
meta-nitrophenyl α-galactoside-based compounds linked to multivalent scaffolds	*V. cholera*	cholera toxin	cells	[95]
GM1-polymer hybrid nanoparticles	*V. cholera*	cholera toxin	animals	[96]
L-fucose analogs, fucosylated glycans, fucose-based polymers	*V. cholera*	cholera toxin	cells	[97]
zinc oxide nanoparticles	*V. cholera*	cholera toxin	cells	[98]
multivalent galactose- and GM1-based derivatives	*V. cholera*	cholera toxin	in vitro	[99]
GM1 ganglioside-like peptide mimic	*V. cholera*	cholera toxin	cells	[100]
decoy exosomes	*S. aureus,*<br>*C. diphtheriae*	α-toxin,<br>diphtheria toxin	animals	[101]
mannose receptor (MRC-1) CTL4 domain-derived peptides loaded into a calcium phosphate nanoparticles (CaP NPs)	*S. pneumonia,*<br>*S. pyogenes,*<br>*L. monocytogenes*	pneumolysin,<br>streptolysin O (SLO)<br>listeriolysin (LLO)	animals	[102]
liposome nanotraps	*S. pneumoniae,*<br>*S. pyogenes,*<br>*S.dysgalactiae*	pneumolysin,<br>streptolysin S (SLS)<br>SLO	cells	[103]
ovine red blood cell membrane (RBCM)-coated PLGA-core nanosponges	*S. pyogenes*	SLO	cells	[104]
redox-responsive mouse RBCM-coated nanogel	*S. aureus*	α-toxin	cells	[105]
human RBCM-coated PLGA-core nanosponges	*S. aureus*<br>*S. pyogenes*<br>*L. monocytogenes*	α-toxin,<br>SLO,<br>LLO	animals	[106]
human RBCM-coated PLGA-core nanosponges	*S. pyogenes*	SLO	animals	[107]
nanogel (human RBCM-coated PLGA-core	*S. pyogenes*	SLO	animals	[108]

nanosponge and chitosan nanoparticle-colloidal gel)				
ssDNA aptamer S3, PEGylated S3	*S. aureus*	staphylococcal enterotoxin A	animals	109
human RBCM-coated nanosponges	Group B *streptococcus*	β-hemolysin / cytolysin	animals	110
rabbit RBCM-coated PLGA-core nanosponges	*E. faecalis*	cytolysin	animals	111
nanoliposome	*S. aureus*	α-toxin	animals	112
sono-immunotherapeutic nanocapturer (MEDI4893 mAb dipslayed on the surface of cell membrane nanovesicles with ultrasound-activated sonosensitizer encapsulation)	*S. aureus*	α-toxin	animals	113
ultrasound-propelled gold nanowires coated with RBC-platelet hybrid membrane	*S. aureus*	α-toxin	cells	114
ssDNA aptamer	*S. aureus*	staphylococcal enterotoxin A	cells	115
protease-conjugated gold nanorods, activated with near infrared light	*S. aureus*	generic exotoxins	in vitro	116
RBCM-coated TR-701-loaded PLGA copolymer nanoparticles (RBCM-PLGA-TR-701NPs)	*S. aureus*	generic exotoxins	cells, animals	117
Ru−Se@GNP-RBCM nanosystem (Ru-complex-functionalized Se nanoparticles loaded into RBCM-coated gelatin nanoparticles)	*S. aureus*	generic exotoxins	animals	118
RBCM-nanosponges (mouse RBC membrane coated PLGA-core)	*S. aureus*	generic exotoxins	animals	119
rabbit and human erythrocyte-derived nanosponges with PLGA cores	*E. faecalis,*<br>*S. pneumoniae*<br>*B. cereus*<br>*S. aureus*	generic exotoxins	animals	120
multivalent N-acetyllactosamine-derived BSA neo-glycoproteins carrying Lewis (Le) blood group antigens	*C. difficile*	TcdA	cells	121
engineered lactobacilli producing VHHs	*C. difficile*	TcdB	animals	122
recombinant mucin-type fusion proteins with a Gal1,3Gal substitution	*C. difficile*	TcdA	cells	123

pectin-derived neoglycolipids, multivalent glycovesicles	*E. coli*	Stx1, Stx2	cells	[124]
pigeon ovalbumin (POA) immobilized gold nanoparticles	*E. coli*	Stx1	cells	[125]
glycopolymers with highly clustered globotriaosyl (Gb3) moieties	*E. coli*	Stx1, Stx2	cells	[126]
cyclodextrin-based Pk-trisaccharide-glycoclusters	*E. coli*	Stx1, Stx2	in vitro	[127]
tetravalent peptides	*E. coli*	Stx	cells	[128]
peptide-based neutralizer (TF-1)	*E. coli*	Stx2	animals	[129]
dipeptides	*C. botulinum*	BoNT/A	in vitro	[130]
function-blocking single domain antibody cargo fused to the BoNT/C1 atoxic derivative (C1ad) delivery vehicle	*C. botulinum*	BoNT/A1	animals	[131]
4-mAb:FP complex (4 biotinylated human/humanized toxin-neutralizing IgGs [6A, 4LCA, 3B3, CR2] adhering to red blood cells via streptavidin-scFv fusionprotein)	*C. botulinum*	BoNT/A	animals	[132]
mouse and human red blood cells expressing chimeric GPA or Kell proteins containing bispesific camelid VHHs	*C. botulinum*	BoNT/A1	animals	[133]
beta-cyclodextrin-derivatives	*C. perfringens*<br>*B. anthracis*	epsilon toxin (Etx), LT-variant of anthrax toxin	cells	[134]
DNA aptamer	*B. anthracis*	PA of anthrax toxin	in vitro	[135]
cationic blockers	*B. anthracis*	PA of anthrax toxin	in vitro	[136]
anthrax decoy protein viz rCMG2-Fc	*B. anthracis*	PA of anthrax toxin	cells	[137]
ssDNA aptamer ML12	*B. anthracis*	PA of anthrax toxin	cells	[138]
fusion protein HSA-CMG2 combining human serum albumin (HSA) and sCMG2 (von Willebrand factor A domain of CMG2)	*B. anthracis*	PA of anthrax toxin	animals	[139]
peptides deriving from human lymphocyte function-associated	*A. actinomycetecomitans*	LtxA	cells	[140]

antigen 1 (LFA-1) β-strands				
human neutrophil peptide 1 (HNP1)	*P. aeruginosa*	ExoA	cells	[141]
**Dominant negative mutants**				
**Lead**	**Pathogen**	**Target**	**Efficacy**	**Ref.**
dominant negative mutant lacking glycine-rich motif	*S. aureus*	LukAB, LukED, HlgAB, HlgCB, and PVL	animals	[142]
**Small molecules**				
**Lead**	**Pathogen**	**Target**	**Efficacy**	**Ref.**
Ebselen (a synthetic, low molecular weight organoselenium compound)	*C. difficile*	TcdA, TcdB	animals	[143, 144]
IP6 analogs (IP2S4, IT2S4)	*C.difficile*	TcdB	animals	[145]
benzodiazepinedione-based inhibitors	*C.difficile*	TcdB	animals	[146, 147]
small-molecule inhibitor (VB-82252)	*C.difficile*	TcdA, TcdB	animals	[148]
bacitracin	*C. difficile*	TcdB	cells	[149]
NSC228155, NSC29193	*B. pertussis*	pertussis toxin	cells	[150]
bifunctional, irreversible inhibitors with a two-pharmacophore design of a ZBG and Cys165-targeting electrophile	*C. botulinum*	BoNT/A	cells	[151]
class of small-molecule selenide compounds	*C. botulinum*	BonT/A	cells	[152]
8-hydroxyquinoline fragment based small molecules (NSC1011 and NSC1014)	*C. botulinum*	BoNT/F	animals	[153]
nitrophenyl psoralen-derivative	*C. botulinum*	BoNT/A	cells	[154]
small molecule mercaptoacetamide metalloprotease inhibitor	*C. botulinum*	BonT/A	animals	[155]
NSC77053, NSC75271, NSC132241, NSC132252, NSC132249	*C. botulinum*	BoNT/E	in vitro	[156, 157]
isoleucine sulfonamide derivatives	*C. botulinum*	BoNT/A	in vitro	[158]
isoflavonoids, acoric acid	*C. botulinum*	BoNT/A	in vitro	[159]

quinolinol compounds (MSU58 and MSU84)	*C. botulinum*	BoNT/A	in vitro	[160]
8-hydroxyquinoline, 8-sulfonamidoquinoline, spiro(indol-thiadiazole), and benzimidazole pyrazolopiperidinone small molecule scaffolds	*C. botulinum*	BoNT/A	in vitro	[161]
quercetin (polyphenol)	*S. suis*	suilysin (SLY)	animals	[162]
morin (flavonol)	*S. suis*	SLY	animals	[163]
quercetin (polyphenol)	*S. pneumoniae*	pneumolysin	animals	[164]
natural sterols	*S. pneumoniae*	pneumolysin	cells	[165]
small molecule epigallocatechin gallate (catechin)efficacy	*S. pneumoniae*	pneumolysin	animals	[166]
betulin (natural lupane-type triterpenoid)	*S. pneumoniae*	pneumolysin	cells	[167]
shikonin (hydroxy-1,4-naphthoquinone)	*S. pneumoniae*	pneumolysin	cells	[168]
hydroxytyrosol	*S. pyogenes*	streptolysin O (SLO)	cells	[169]
baicalein (flavone)	*L. monocytogenes*	listeriolysin (LLO)	animals	[170]
lutein (Xanthophyll, a natural small molecule)	*L. monocytogenes*	LLO	animals	[171]
phosphocholine	*L. monocytogenes,*<br>*S. pneumoniae,*<br>*S. pyogenes*	LLO,<br>pneumolysin,<br>SLO	cells	[172]
myricetin	*S. aureus*	α-toxin	animals	[173]
aloe emodin (anthraquinone)	*S. aureus*	α-toxin	animals	[174]
n-tetradecyl-phosphocholine (C14PC)	*S. aureus*	α-toxin,<br>PVL,<br>LukED	cells	[175]
N-aryl mercapto-acetamide	*P .aeruginosa*	pseudolysin (LasB)	animals	[176]
hydroxamate derivative	*P. aeruginosa*	LasB	cells	[177]
mercaptoacetamide compounds and corresponding thioacetate prodrugs	*P. aeruginosa*	LasB	animals	[178]
N-aryl mercaptoacetamide	*C. histolyticum*	collagenases (ColH, ColG, ColT, ColQ1)	in vitro	[179]
amide based small molecules	*S. dysenteriae,*<br>*E. coli*	Stx	animals	[180]
colistin, amoxicillin and fluoroquinolones	*E. coli*	Stx2	cells	[181]
verbascoside (a phenylpropanoid glycoside)	*C. perfringens*	alpha toxin (CPA), perfingolysin O (PFO)	animals	[182]
semicarbazone EGA	*C. difficile,*<br>*C. perfringens,*	cytolethal distending	cells	[183]

	C. botulinum	toxin (CDT),<br>iota toxin (ITX),<br>C2 toxin		
bacitracin	*C. botulinum,*<br>*C. perfringens,*<br>*C. difficile,*<br>*B. anthracis*	C2 toxin,<br>ITX,<br>CDT,<br>LT-variant of anthrax toxin	cells	[184]
aminoquinolinium salts (chloroquine analogs)	*C. botulinum,*<br>*C. perfringens*	C2 toxin,<br>ITX	cells	[185]
chloroquine derivatives	*C. botulinum,*<br>*B. anthracis*	C2 toxin,<br>LT-variant of anthrax toxin	cells	[186]
5'- Fluorosulfonylbenzoyl 5'-adenosine (FSBA) derivates	*B. anthracis*	EF of anthrax toxin	cells	[187]
semicarbazone EGA	*C. diphtheriae*	diphtheria toxin	cells	[188]
epigallocatechingallate, procyanidin B2	*V. cholera*	cholera toxin	cells	[189]
allicin (diallyl thiosulfinate)	*S. pyogenes*	SpeB	in vitro	[190]
2S-alkyne	*S. pyogenes*	SpeB	cells	[191]

[a]Information in this table is composed of primary research articles published on exotoxin-targeted drug modalities in the past 5 years. The mode of action at the molecular level is not known for some of the leads. Classification is based on the six exotoxin-targeted drug modalities, as specified in the main text. Antibodies and antibody fragments are combined in this table, because some leads are composed of both of these modalities. Moreover, mAb engineering frequently utilizes antibody fragment step, and subsequent linking of the most efficient fragments with Fc-region to engage effector functions of entire mAbs.

REFERENCES

1. Hu, N.; Qiao, C.; Wang, J.; Wang, Z.; Li, X.; Zhou, L.; Wu, J.; Zhang, D.; Feng, J.; Shen, B.; Zhang, J.; Luo, L., Identification of a novel protective human monoclonal antibody, LXY8, that targets the key neutralizing epitopes of staphylococcal enterotoxin B. *Biochem Biophys Res Commun* **2021,** *549*, 120-127. DOI: 10.1016/j.bbrc.2021.02.057.
2. Liu, Y.; Song, Z.; Ge, S.; Zhang, J.; Xu, L.; Yang, F.; Lu, D.; Luo, P.; Gu, J.; Zou, Q.; Zeng, H., Determining the immunological characteristics of a novel human monoclonal antibody developed against staphylococcal enterotoxin B. *Hum Vaccin Immunother* **2020,** *16* (7), 1708-1718. DOI: 10.1080/21645515.2020.1744362.
3. Chen, G.; Karauzum, H.; Long, H.; Carranza, D.; Holtsberg, F. W.; Howell, K. A.; Abaandou, L.; Zhang, B.; Jarvik, N.; Ye, W.; Liao, G. C.; Gross, M. L.; Leung, D. W.; Amarasinghe, G. K.; Aman, M. J.; Sidhu, S. S., Potent neutralization of Staphylococcal enterotoxin B in vivo by antibodies that block binding to the T-cell receptor. *J Mol Biol* **2019,** *431* (21), 4354-4367. DOI: 10.1016/j.jmb.2019.03.017.
4. Verreault, D.; Ennis, J.; Whaley, K.; Killeen, S. Z.; Karauzum, H.; Aman, M. J.; Holtsberg, R.; Doyle-Meyers, L.; Didier, P. J.; Zeitlin, L.; Roy, C. J., Effective Treatment of Staphylococcal enterotoxin B aerosol intoxication in rhesus macaques by using two parenterally administered high-affinity monoclonal antibodies. *Antimicrob Agents Chemother* **2019,** *63* (5). DOI: 10.1128/AAC.02049-18.
5. Kroetsch, A.; Qiao, C.; Heavey, M.; Guo, L.; Shah, D. K.; Park, S., Engineered pH-dependent recycling antibodies enhance elimination of Staphylococcal enterotoxin B superantigen in mice. *MAbs* **2019,** *11* (2), 411-421. DOI: 10.1080/19420862.2018.1545510.
6. Karau, M. J.; Tilahun, M. E.; Krogman, A.; Osborne, B. A.; Goldsby, R. A.; David, C. S.; Mandrekar, J. N.; Patel, R.; Rajagopalan, G., Passive therapy with humanized anti-staphylococcal enterotoxin B antibodies attenuates systemic inflammatory response and protects from lethal pneumonia caused by staphylococcal enterotoxin B-producing Staphylococcus aureus. *Virulence* **2017,** *8* (7), 1148-1159. DOI: 10.1080/21505594.2016.1267894.
7. Ortines, R. V.; Liu, H.; Cheng, L. I.; Cohen, T. S.; Lawlor, H.; Gami, A.; Wang, Y.; Dillen, C. A.; Archer, N. K.; Miller, R. J.; Ashbaugh, A. G.; Pinsker, B. L.; Marchitto, M. C.; Tkaczyk, C.; Stover, C. K.; Sellman, B. R.; Miller, L. S., Neutralizing alpha-toxin accelerates healing of Staphylococcus aureus-infected wounds in nondiabetic and diabetic mice. *Antimicrob Agents Chemother* **2018,** *62* (3). DOI: 10.1128/AAC.02288-17.
8. Tkaczyk, C.; Kasturirangan, S.; Minola, A.; Jones-Nelson, O.; Gunter, V.; Shi, Y. Y.; Rosenthal, K.; Aleti, V.; Semenova, E.; Warrener, P.; Tabor, D.; Stover, C. K.; Corti, D.; Rainey, G.; Sellman, B. R., Multimechanistic monoclonal antibodies (mAbs) targeting Staphylococcus aureus alpha-toxin and clumping factor A: Activity and afficacy comparisons of a mAb combination and an engineered bispecific antibody approach. *Antimicrob Agents Chemother* **2017,** *61* (8). DOI: 10.1128/AAC.00629-17.
9. Aguilar, J. L.; Varshney, A. K.; Pechuan, X.; Dutta, K.; Nosanchuk, J. D.; Fries, B. C., Monoclonal antibodies protect from Staphylococcal Enterotoxin K (SEK) induced toxic shock and sepsis by USA300 Staphylococcus aureus. *Virulence* **2017,** *8* (6), 741-750. DOI: 10.1080/21505594.2016.1231295.

10. Aguilar, J. L.; Varshney, A. K.; Wang, X.; Stanford, L.; Scharff, M.; Fries, B. C., Detection and measurement of Staphylococcal enterotoxin-like K (SEl-K) secretion by Staphylococcus aureus clinical isolates. *J Clin Microbiol* **2014,** *52* (7), 2536-43. DOI: 10.1128/JCM.00387-14.
11. Snow, D. M.; Cobb, R. R.; Martinez, J.; Finger-Baker, I.; Collins, L.; Terpening, S.; Syar, E. S.; Niemuth, N.; Kobs, D.; Barnewall, R.; Farr-Jones, S.; Marks, J. D.; Tomic, M. T., A Monoclonal antibody combination against both serotypes A and B botulinum toxin prevents inhalational botulism in a guinea pig model. *Toxins (Basel)* **2021,** *13* (1). DOI: 10.3390/toxins13010031.
12. Matsumura, T.; Amatsu, S.; Misaki, R.; Yutani, M.; Du, A.; Kohda, T.; Fujiyama, K.; Ikuta, K.; Fujinaga, Y., Fully human monoclonal antibodies effectively neutralizing botulinum neurotoxin serotype B. *Toxins (Basel)* **2020,** *12* (5). DOI: 10.3390/toxins12050302.
13. Xiong, X.; Lv, S.; Fu, C.; Li, L.; Sun, Z.; Han, X.; Zhang, W., Production and characterization of a neutralizing antibody against botulinum neurotoxin A. *J Immunol Methods* **2020,** *487*, 112871. DOI: 10.1016/j.jim.2020.112871.
14. Tomic, M. T.; Espinoza, Y.; Martinez, Z.; Pham, K.; Cobb, R. R.; Snow, D. M.; Earnhart, C. G.; Pals, T.; Syar, E. S.; Niemuth, N.; Kobs, D. J.; Farr-Jones, S.; Marks, J. D., Monoclonal antibody combinations prevent serotype A and serotype B inhalational botulism in a guinea pig model. *Toxins (Basel)* **2019,** *11* (4). DOI: 10.3390/toxins11040208.
15. Lou, J.; Wen, W.; Conrad, F.; Meng, Q.; Dong, J.; Sun, Z.; Garcia-Rodriguez, C.; Farr-Jones, S.; Cheng, L. W.; Henderson, T. D.; Brown, J. L.; Smith, T. J.; Smith, L. A.; Cormier, A.; Marks, J. D., A single tri-Epitopic antibody virtually recapitulates the potency of a combination of three monoclonal antibodies in neutralization of botulinum neurotoxin serotype A. *Toxins (Basel)* **2018,** *10* (2). DOI: 10.3390/toxins10020084.
16. Fan, Y.; Garcia-Rodriguez, C.; Lou, J.; Wen, W.; Conrad, F.; Zhai, W.; Smith, T. J.; Smith, L. A.; Marks, J. D., A three monoclonal antibody combination potently neutralizes multiple botulinum neurotoxin serotype F subtypes. *PLoS One* **2017,** *12* (3), e0174187. DOI: 10.1371/journal.pone.0174187.
17. Derman, Y.; Selby, K.; Miethe, S.; Frenzel, A.; Liu, Y.; Rasetti-Escargueil, C.; Avril, A.; Pelat, T.; Urbain, R.; Fontayne, A.; Thullier, P.; Sesardic, D.; Lindström, M.; Hust, M.; Korkeala, H., Neutralization of botulinum neurotoxin type E by a humanized antibody. *Toxins (Basel)* **2016,** *8* (9). DOI: 10.3390/toxins8090257.
18. Fan, Y.; Barash, J. R.; Lou, J.; Conrad, F.; Marks, J. D.; Arnon, S. S., Immunological characterization and neutralizing ability of monoclonal antibodies directed against botulinum neurotoxin type H. *J Infect Dis* **2016,** *213* (10), 1606-14. DOI: 10.1093/infdis/jiv770.
19. Miethe, S.; Mazuet, C.; Liu, Y.; Tierney, R.; Rasetti-Escargueil, C.; Avril, A.; Frenzel, A.; Thullier, P.; Pelat, T.; Urbain, R.; Fontayne, A.; Sesardic, D.; Hust, M.; Popoff, M. R., Development of germline-humanized antibodies neutralizing botulinum neurotoxin A and B. *PLoS One* **2016,** *11* (8), e0161446. DOI: 10.1371/journal.pone.0161446.
20. Godakova, S. A.; Noskov, A. N.; Vinogradova, I. D.; Ugriumova, G. A.; Solovyev, A. I.; Esmagambetov, I. B.; Tukhvatulin, A. I.; Logunov, D. Y.; Naroditsky, B. S.; Shcheblyakov, D. V.; Gintsburg, A. L., Camelid VHHs fused to human Fc fragments provide long term protection against botulinum neurotoxin A in mice. *Toxins (Basel)* **2019,** *11* (8). DOI: 10.3390/toxins11080464.

21. Lam, K. H.; Tremblay, J. M.; Vazquez-Cintron, E.; Perry, K.; Ondeck, C.; Webb, R. P.; McNutt, P. M.; Shoemaker, C. B.; Jin, R., Structural insights into rational design of single-domain antibody-based antitoxins against botulinum neurotoxins. *Cell Rep* **2020,** *30* (8), 2526-2539.e6. DOI: 10.1016/j.celrep.2020.01.107.
22. Yao, G.; Lam, K. H.; Weisemann, J.; Peng, L.; Krez, N.; Perry, K.; Shoemaker, C. B.; Dong, M.; Rummel, A.; Jin, R., A camelid single-domain antibody neutralizes botulinum neurotoxin A by blocking host receptor binding. *Sci Rep* **2017,** *7* (1), 7438. DOI: 10.1038/s41598-017-07457-5.
23. Lam, K. H.; Perry, K.; Shoemaker, C. B.; Jin, R., Two VHH Antibodies neutralize botulinum neurotoxin E1 by blocking its membrane translocation in host cells. *Toxins (Basel)* **2020,** *12* (10). DOI: 10.3390/toxins12100616.
24. Tremblay, J. M.; Vazquez-Cintron, E.; Lam, K. H.; Mukherjee, J.; Bedenice, D.; Ondeck, C. A.; Conroy, M. T.; Bodt, S. M. L.; Winner, B. M.; Webb, R. P.; Ichtchenko, K.; Jin, R.; McNutt, P. M.; Shoemaker, C. B., Camelid VHH Antibodies that Neutralize Botulinum Neurotoxin Serotype E Intoxication or Protease Function. *Toxins (Basel)* **2020,** *12* (10). DOI: 10.3390/toxins12100611.
25. Baghban, R.; Gargari, S. L.; Rajabibazl, M.; Nazarian, S.; Bakherad, H., Camelid-derived heavy-chain nanobody against Clostridium botulinum neurotoxin E in Pichia pastoris. *Biotechnol Appl Biochem* **2016,** *63* (2), 200-5. DOI: 10.1002/bab.1226.
26. Hernandez, D. N.; Tam, K.; Shopsin, B.; Radke, E. E.; Law, K.; Cardozo, T.; Torres, V. J.; Silverman, G. J., Convergent evolution of neutralizing antibodies to Staphylococcus aureus γ-hemolysin C that recognize an immunodominant primary sequence-dependent B-cell epitope. *mBio* **2020,** *11* (3). DOI: 10.1128/mBio.00460-20.
27. Ghotloo, S.; Amiri, M. M.; Khoshnoodi, J.; Abbasi, E.; Jeddi-Tehrani, M.; Golsaz-Shirazi, F.; Shokri, F., Contribution of Fc fragment of monoclonal antibodies to tetanus toxin neutralization. *Neurotox Res* **2020,** *37* (3), 578-586. DOI: 10.1007/s12640-019-00124-9.
28. Aliprandini, E.; Takata, D. Y.; Lepique, A.; Kalil, J.; Boscardin, S. B.; Moro, A. M., An oligoclonal combination of human monoclonal antibodies able to neutralize tetanus toxin. *Toxicon X* **2019,** *2*, 100006. DOI: 10.1016/j.toxcx.2019.100006.
29. Wang, H.; Yu, R.; Fang, T.; Yu, T.; Chi, X.; Zhang, X.; Liu, S.; Fu, L.; Yu, C.; Chen, W., Tetanus neurotoxin neutralizing antibodies screened from a human immune scFv antibody phage display library. *Toxins (Basel)* **2016,** *8* (9). DOI: 10.3390/toxins8090266.
30. Yousefi, M.; Younesi, V.; Bayat, A. A.; Jadidi-Niaragh, F.; Abbasi, E.; Razavi, A.; Khosravi-Eghbal, R.; Asgarian-Omran, H.; Shokri, F., Comparative human and mouse antibody responses against tetanus toxin at clonal level. *J Immunotoxicol* **2016,** *13* (2), 243-8. DOI: 10.3109/1547691X.2015.1046572.
31. Kucinskaite-Kodze, I.; Simanavicius, M.; Dapkunas, J.; Pleckaityte, M.; Zvirbliene, A., Mapping of recognition sites of monoclonal antibodies responsible for the inhibition of pneumolysin functional activity. *Biomolecules* **2020,** *10* (7). DOI: 10.3390/biom10071009.
32. Rudenko, N.; Nagel, A.; Zamyatina, A.; Karatovskaya, A.; Salyamov, V.; Andreeva-Kovalevskaya, Z.; Siunov, A.; Kolesnikov, A.; Shepelyakovskaya, A.; Boziev, K.; Melnik, B.; Brovko, F.; Solonin, A., A monoclonal antibody against the C-terminal domain of Bacillus cereus hemolysin II inhibits HlyII cytolytic activity. *Toxins (Basel)* **2020,** *12* (12). DOI: 10.3390/toxins12120806.

33. Vu, T. T. T.; Nguyen, N. T. Q.; Tran, V. G.; Gras, E.; Mao, Y.; Jung, D. H.; Tkaczyk, C.; Sellman, B. R.; Diep, B. A., Protective efficacy of monoclonal antibodies neutralizing alpha-hemolysin and bicomponent leukocidins in a rabbit model of Staphylococcus aureus necrotizing pneumonia. *Antimicrob Agents Chemother* **2020,** *64* (3). DOI: 10.1128/AAC.02220-19.
34. Jing, C.; Liu, C.; Liu, F.; Gao, Y.; Liu, Y.; Guan, Z.; Xuan, B.; Yu, Y.; Yang, G., Novel human monoclonal antibodies targeting the F subunit of leukocidins reduce disease progression and mortality caused by Staphylococcus aureus. *BMC Microbiol* **2018,** *18* (1), 181. DOI: 10.1186/s12866-018-1312-7.
35. Badarau, A.; Rouha, H.; Malafa, S.; Battles, M. B.; Walker, L.; Nielson, N.; Dolezilkova, I.; Teubenbacher, A.; Banerjee, S.; Maierhofer, B.; Weber, S.; Stulik, L.; Logan, D. T.; Welin, M.; Mirkina, I.; Pleban, C.; Zauner, G.; Gross, K.; Jägerhofer, M.; Magyarics, Z.; Nagy, E., Context matters: The importance of dimerization-induced conformation of the LukGH leukocidin of Staphylococcus aureus for the generation of neutralizing antibodies. *MAbs* **2016,** *8* (7), 1347-1360. DOI: 10.1080/19420862.2016.1215791.
36. Thomsen, I. P.; Sapparapu, G.; James, D. B. A.; Cassat, J. E.; Nagarsheth, M.; Kose, N.; Putnam, N.; Boguslawski, K. M.; Jones, L. S.; Wood, J. B.; Creech, C. B.; Torres, V. J.; Crowe, J. E., Monoclonal antibodies against the Staphylococcus aureus bicomponent leukotoxin AB isolated following invasive human infection reveal diverse binding and modes of action. *J Infect Dis* **2017,** *215* (7), 1124-1131. DOI: 10.1093/infdis/jix071.
37. Rukkawattanakul, T.; Sookrung, N.; Seesuay, W.; Onlamoon, N.; Diraphat, P.; Chaicumpa, W.; Indrawattana, N., Human scFvs that counteract bioactivities of Staphylococcus aureus TSST-1. *Toxins (Basel)* **2017,** *9* (2). DOI: 10.3390/toxins9020050.
38. Wenzel, E. V.; Bosnak, M.; Tierney, R.; Schubert, M.; Brown, J.; Dübel, S.; Efstratiou, A.; Sesardic, D.; Stickings, P.; Hust, M., Human antibodies neutralizing diphtheria toxin in vitro and in vivo. *Sci Rep* **2020,** *10* (1), 571. DOI: 10.1038/s41598-019-57103-5.
39. Zhu, S.; Liuni, P.; Ettorre, L.; Chen, T.; Szeto, J.; Carpick, B.; James, D. A.; Wilson, D. J., Hydrogen-deuterium exchange epitope mapping reveals distinct neutralizing mechanisms for two monoclonal antibodies against diphtheria toxin. *Biochemistry* **2019,** *58* (6), 646-656. DOI: 10.1021/acs.biochem.8b01123.
40. Lakzaei, M.; Rasaee, M. J.; Fazaeli, A. A.; Aminian, M., A comparison of three strategies for biopanning of phage-scFv library against diphtheria toxin. *J Cell Physiol* **2019,** *234* (6), 9486-9494. DOI: 10.1002/jcp.27636.
41. Ahn, B. E.; Bae, H. W.; Lee, H. R.; Woo, S. J.; Park, O. K.; Jeon, J. H.; Park, J.; Rhie, G. E., A therapeutic human antibody against the domain 4 of the Bacillus anthracis protective antigen shows protective efficacy in a mouse model. *Biochem Biophys Res Commun* **2019,** *509* (2), 611-616. DOI: 10.1016/j.bbrc.2018.12.146.
42. Tang, Q.; Xiong, S.; Liang, X.; Kuai, X.; Wang, Y.; Wang, C.; Feng, Z.; Zhu, J., Human monoclonal anti-protective antigen antibody for the low-dose post-exposure prophylaxis and treatment of Anthrax. *BMC Infect Dis* **2018,** *18* (1), 640. DOI: 10.1186/s12879-018-3542-6.
43. Xiong, S.; Zhou, T.; Zheng, F.; Liang, X.; Cao, Y.; Wang, C.; Feng, Z.; Tang, Q.; Zhu, J., Different mechanisms of two anti-anthrax protective antigen antibodies and function comparison between them. *BMC Infect Dis* **2019,** *19* (1), 940. DOI: 10.1186/s12879-019-4508-z.

44. Shali, A.; Hasannia, S.; Gashtasbi, F.; Abdous, M.; Shahangian, S. S.; Jalili, S., Generation and screening of efficient neutralizing single domain antibodies (VHHs) against the critical functional domain of anthrax protective antigen (PA). *Int J Biol Macromol* **2018,** *114*, 1267-1278. DOI: 10.1016/j.ijbiomac.2018.03.034.
45. Moayeri, M.; Tremblay, J. M.; Debatis, M.; Dmitriev, I. P.; Kashentseva, E. A.; Yeh, A. J.; Cheung, G. Y.; Curiel, D. T.; Leppla, S.; Shoemaker, C. B., Adenoviral expression of a bispecific VHH-based neutralizing agent that targets protective antigen provides prophylactic protection from anthrax in mice. *Clin Vaccine Immunol* **2016,** *23* (3), 213-8. DOI: 10.1128/CVI.00611-15.
46. Zhang, D.; Liu, W.; Wen, Z.; Li, B.; Liu, S.; Li, J.; Chen, W., Establishment of a New Zealand white rabbit model for lethal toxin (LT) challenge and efficacy of monoclonal antibody 5E11 in the LT-challenged rabbit model. *Toxins (Basel)* **2018,** *10* (7). DOI: 10.3390/toxins10070289.
47. Liang, Y.; Coffin, M. V.; Manceva, S. D.; Chichester, J. A.; Jones, R. M.; Kiick, K. L., Controlled release of an anthrax toxin-neutralizing antibody from hydrolytically degradable polyethylene glycol hydrogels. *J Biomed Mater Res A* **2016,** *104* (1), 113-23. DOI: 10.1002/jbm.a.35545.
48. Farcasanu, M.; Wang, A. G.; Uchański, T.; Bailey, L. J.; Yue, J.; Chen, Z.; Wu, X.; Kossiakoff, A.; Tang, W. J., Rapid discovery and characterization of synthetic neutralizing antibodies against anthrax edema toxin. *Biochemistry* **2019,** *58* (27), 2996-3004. DOI: 10.1021/acs.biochem.9b00184.
49. Vrentas, C. E.; Moayeri, M.; Keefer, A. B.; Greaney, A. J.; Tremblay, J.; O'Mard, D.; Leppla, S. H.; Shoemaker, C. B., A diverse set of single-domain antibodies (VHHs) against the anthrax toxin lethal and edema factors provides a basis for construction of a bispecific agent that protects against anthrax infection. *J Biol Chem* **2016,** *291* (41), 21596-21606. DOI: 10.1074/jbc.M116.749184.
50. Acquaye-Seedah, E.; Huang, Y.; Sutherland, J. N.; DiVenere, A. M.; Maynard, J. A., Humanised monoclonal antibodies neutralise pertussis toxin by receptor blockade and reduced retrograde trafficking. *Cell Microbiol* **2018,** *20* (12), e12948. DOI: 10.1111/cmi.12948.
51. Nguyen, A. W.; Wagner, E. K.; Laber, J. R.; Goodfield, L. L.; Smallridge, W. E.; Harvill, E. T.; Papin, J. F.; Wolf, R. F.; Padlan, E. A.; Bristol, A.; Kaleko, M.; Maynard, J. A., A cocktail of humanized anti-pertussis toxin antibodies limits disease in murine and baboon models of whooping cough. *Sci Transl Med* **2015,** *7* (316), 316ra195. DOI: 10.1126/scitranslmed.aad0966.
52. Nguyen, A. W.; DiVenere, A. M.; Papin, J. F.; Connelly, S.; Kaleko, M.; Maynard, J. A., Neutralization of pertussis toxin by a single antibody prevents clinical pertussis in neonatal baboons. *Sci Adv* **2020,** *6* (6), eaay9258. DOI: 10.1126/sciadv.aay9258.
53. Wagner, E. K.; Wang, X.; Bui, A.; Maynard, J. A., Synergistic neutralization of pertussis toxin by a bispecific antibody in vitro and in vivo. *Clin Vaccine Immunol* **2016,** *23* (11), 851-862. DOI: 10.1128/CVI.00371-16.
54. Cole, L. E.; Li, L.; Jetley, U.; Zhang, J.; Pacheco, K.; Ma, F.; Mundle, S.; Yan, Y.; Barone, L.; Rogers, C.; Beltraminelli, N.; Quemeneur, L.; Kleanthous, H.; Anderson, S. F.; Anosova, N. G., Deciphering the domain specificity of C. difficile toxin neutralizing antibodies. *Vaccine* **2019,** *37* (29), 3892-3901. DOI: 10.1016/j.vaccine.2019.05.040.

55. Anosova, N. G.; Cole, L. E.; Li, L.; Zhang, J.; Brown, A. M.; Mundle, S.; Ray, S.; Ma, F.; Garrone, P.; Bertraminelli, N.; Kleanthous, H.; Anderson, S. F., A combination of three fully human toxin A- and toxin B-specific monoclonal antibodies protects against challenge with highly virulent epidemic strains of Clostridium difficile in the hamster model. *Clin Vaccine Immunol* **2015,** *22* (7), 711-25. DOI: 10.1128/CVI.00763-14.
56. Kroh, H. K.; Chandrasekaran, R.; Rosenthal, K.; Woods, R.; Jin, X.; Ohi, M. D.; Nyborg, A. C.; Rainey, G. J.; Warrener, P.; Spiller, B. W.; Lacy, D. B., Use of a neutralizing antibody helps identify structural features critical for binding of. *J Biol Chem* **2017,** *292* (35), 14401-14412. DOI: 10.1074/jbc.M117.781112.
57. Marozsan, A. J.; Ma, D.; Nagashima, K. A.; Kennedy, B. J.; Kang, Y. K.; Arrigale, R. R.; Donovan, G. P.; Magargal, W. W.; Maddon, P. J.; Olson, W. C., Protection against Clostridium difficile infection with broadly neutralizing antitoxin monoclonal antibodies. *J Infect Dis* **2012,** *206* (5), 706-13. DOI: 10.1093/infdis/jis416.
58. Qiu, H.; Cassan, R.; Johnstone, D.; Han, X.; Joyee, A. G.; McQuoid, M.; Masi, A.; Merluza, J.; Hrehorak, B.; Reid, R.; Kennedy, K.; Tighe, B.; Rak, C.; Leonhardt, M.; Dupas, B.; Saward, L.; Berry, J. D.; Nykiforuk, C. L., Novel Clostridium difficile anti-toxin (TcdA and TcdB) humanized monoclonal antibodies demonstrate in vitro neutralization across a broad spectrum of clinical strains and in vivo potency in a hamster spore challenge model. *PLoS One* **2016,** *11* (6), e0157970. DOI: 10.1371/journal.pone.0157970.
59. Kroh, H. K.; Chandrasekaran, R.; Zhang, Z.; Rosenthal, K.; Woods, R.; Jin, X.; Nyborg, A. C.; Rainey, G. J.; Warrener, P.; Melnyk, R. A.; Spiller, B. W.; Lacy, D. B., A neutralizing antibody that blocks delivery of the enzymatic cargo of Clostridium difficile toxin TcdB into host cells. *J Biol Chem* **2018,** *293* (3), 941-952. DOI: 10.1074/jbc.M117.813428.
60. Sulea, T.; Hussack, G.; Ryan, S.; Tanha, J.; Purisima, E. O., Application of assisted design of antibody and protein therapeutics (ADAPT) improves efficacy of a Clostridium difficile toxin A single-domain antibody. *Sci Rep* **2018,** *8* (1), 2260. DOI: 10.1038/s41598-018-20599-4.
61. Hussack, G.; Ryan, S.; van Faassen, H.; Rossotti, M.; MacKenzie, C. R.; Tanha, J., Neutralization of Clostridium difficile toxin B with VHH-Fc fusions targeting the delivery and CROPs domains. *PLoS One* **2018,** *13* (12), e0208978. DOI: 10.1371/journal.pone.0208978.
62. Schmidt, D. J.; Beamer, G.; Tremblay, J. M.; Steele, J. A.; Kim, H. B.; Wang, Y.; Debatis, M.; Sun, X.; Kashentseva, E. A.; Dmitriev, I. P.; Curiel, D. T.; Shoemaker, C. B.; Tzipori, S., A tetraspecific VHH-based neutralizing antibody modifies disease outcome in three animal models of Clostridium difficile infection. *Clin Vaccine Immunol* **2016,** *23* (9), 774-84. DOI: 10.1128/CVI.00730-15.
63. Chen, P.; Lam, K. H.; Liu, Z.; Mindlin, F. A.; Chen, B.; Gutierrez, C. B.; Huang, L.; Zhang, Y.; Hamza, T.; Feng, H.; Matsui, T.; Bowen, M. E.; Perry, K.; Jin, R., Structure of the full-length Clostridium difficile toxin B. *Nat Struct Mol Biol* **2019,** *26* (8), 712-719. DOI: 10.1038/s41594-019-0268-0.
64. Li, S.; Shi, L.; Yang, Z.; Zhang, Y.; Perez-Cordon, G.; Huang, T.; Ramsey, J.; Oezguen, N.; Savidge, T. C.; Feng, H., Critical roles of Clostridium difficile toxin B enzymatic activities in pathogenesis. *Infect Immun* **2015,** *83* (2), 502-13. DOI: 10.1128/IAI.02316-14.

65. Führer, V.; Heine, P. A.; Helmsing, S.; Goy, S.; Heidepriem, J.; Loeffler, F. F.; Dübel, S.; Gerhard, R.; Hust, M., Development of neutralizing and non-neutralizing antibodies targeting known and novel epitopes of TcdB of Clostridioides difficile. *Front Microbiol* **2018,** *9*, 2908. DOI: 10.3389/fmicb.2018.02908.
66. Linden, J. R.; Telesford, K.; Shetty, S.; Winokour, P.; Haigh, S.; Cahir-McFarland, E.; Antognetti, G.; Datta, A.; Wang, T.; Meier, W.; Vartanian, T., A novel panel of rabbit monoclonal antibodies and their diverse applications including inhibition of Clostridium perfringens epsilon toxin oligomerization. *Antibodies (Basel)* **2018,** *7* (4). DOI: 10.3390/antib7040037.
67. Lee, T. H.; Cha, S. S.; Lee, C. S.; Rhee, J. H.; Woo, H. R.; Chung, K. M., Cross-protection against Vibrio cholerae infection by monoclonal antibodies against Vibrio vulnificus RtxA1/MARTX$_{Vv}$. *Microbiol Immunol* **2016,** *60* (11), 793-800. DOI: 10.1111/1348-0421.12449.
68. Zeng, J.; Song, F.; Yang, Y.; Ma, C.; Deng, G.; Li, Y.; Wang, Y.; Liu, X., The generation and characterization of recombinant protein and antibodies of Clostridium perfringens beta2 toxin. *J Immunol Res* **2016,** *2016*, 5708468. DOI: 10.1155/2016/5708468.
69. Matsumura, T.; Nishiyama, A.; Aiko, M.; Ainai, A.; Ikebe, T.; Chiba, J.; Ato, M.; Takahashi, Y., An anti-perfringolysin O monoclonal antibody cross-reactive with streptolysin O protects against streptococcal toxic shock syndrome. *BMC Res Notes* **2020,** *13* (1), 419. DOI: 10.1186/s13104-020-05264-2.
70. Jiao, X.; Smith, S.; Stack, G.; Liang, Q.; Bradley, A.; Kellam, P.; Galán, J. E., Generation and characterization of typhoid toxin-neutralizing human monoclonal antibodies. *Infect Immun* **2020,** *88* (10). DOI: 10.1128/IAI.00292-20.
71. Forti, K.; Cagiola, M.; Pellegrini, M.; Anzalone, L.; Di Paolo, A.; Corneli, S.; Severi, G.; De Giuseppe, A., Generation of recombinant baculovirus expressing atoxic C-terminal CPA toxin of Clostridium perfringens and production of specific antibodies. *BMC Biotechnol* **2020,** *20* (1), 7. DOI: 10.1186/s12896-019-0597-4.
72. Wang, D.; Yue, Y.; Wu, G.; Tian, Y.; Liu, Y.; Yu, J.; Ji, Y.; Wang, J.; Li, J.; Pan, R.; Ma, H.; Zhang, G., Preparation and characterization of a human scFv against the Clostridium perfringens type A alpha-toxin. *Toxicon* **2017,** *130*, 79-86. DOI: 10.1016/j.toxicon.2017.02.021.
73. Mootien, S.; Kaplan, P. M., Monoclonal antibodies specific for Bacteroides fragilis enterotoxins BFT1 and BFT2 and their use in immunoassays. *PLoS One* **2017,** *12* (3), e0173128. DOI: 10.1371/journal.pone.0173128.
74. Didier, A.; Dietrich, R.; Märtlbauer, E., Antibody binding studies reveal conformational flexibility of the Bacillus cereus non-hemolytic enterotoxin (Nhe) A-component. *PLoS One* **2016,** *11* (10), e0165135. DOI: 10.1371/journal.pone.0165135.
75. Fahimi, F.; Sarhaddi, S.; Fouladi, M.; Samadi, N.; Sadeghi, J.; Golchin, A.; Tohidkia, M. R.; Barar, J.; Omidi, Y., Phage display-derived antibody fragments against conserved regions of VacA toxin of Helicobacter pylori. *Appl Microbiol Biotechnol* **2018,** *102* (16), 6899-6913. DOI: 10.1007/s00253-018-9068-4.
76. Skinner, C.; Patfield, S.; Khalil, R.; Kong, Q.; He, X., New monoclonal antibodies against a novel subtype of Shiga toxin 1 produced by Enterobacter cloacae and their use in analysis of human serum. *mSphere* **2016,** *1* (1). DOI: 10.1128/mSphere.00099-15.
77. Bernedo-Navarro, R. A.; Romão, E.; Yano, T.; Pinto, J.; De Greve, H.; Sterckx, Y. G.; Muyldermans, S., Structural basis for the specific neutralization of Stx2a with a camelid single domain antibody fragment. *Toxins (Basel)* **2018,** *10* (3). DOI: 10.3390/toxins10030108.

78. Luz, D.; Amaral, M. M.; Sacerdoti, F.; Bernal, A. M.; Quintilio, W.; Moro, A. M.; Palermo, M. S.; Ibarra, C.; Piazza, R. M. F., Human recombinant Fab fragment neutralizes Shiga toxin type 2 cytotoxic effects. *Toxins (Basel)* **2018,** *10* (12). DOI: 10.3390/toxins10120508.
79. Mejías, M. P.; Hiriart, Y.; Lauché, C.; Fernández-Brando, R. J.; Pardo, R.; Bruballa, A.; Ramos, M. V.; Goldbaum, F. A.; Palermo, M. S.; Zylberman, V., Development of camelid single chain antibodies against Shiga toxin type 2 (Stx2) with therapeutic potential against hemolytic uremic syndrome (HUS). *Sci Rep* **2016,** *6*, 24913. DOI: 10.1038/srep24913.
80. Malik, A. A.; Imtong, C.; Sookrung, N.; Katzenmeier, G.; Chaicumpa, W.; Angsuthanasombat, C., Structural characterization of humanized nanobodies with neutralizing activity against the Bordetella pertussis CyaA-hemolysin: Implications for a potential epitope of toxin-protective antigen. *Toxins (Basel)* **2016,** *8* (4), 99. DOI: 10.3390/toxins8040099.
81. Wang, X.; Gray, M. C.; Hewlett, E. L.; Maynard, J. A., The Bordetella adenylate cyclase repeat-in-toxin (RTX) domain is immunodominant and elicits neutralizing antibodies. *J Biol Chem* **2015,** *290* (6), 3576-91. DOI: 10.1074/jbc.M114.585281.
82. Wang, X.; Stapleton, J. A.; Klesmith, J. R.; Hewlett, E. L.; Whitehead, T. A.; Maynard, J. A., Fine epitope mapping of two antibodies neutralizing the Bordetella adenylate cyclase toxin. *Biochemistry* **2017,** *56* (9), 1324-1336. DOI: 10.1021/acs.biochem.6b01163.
83. Santajit, S.; Seesuay, W.; Mahasongkram, K.; Sookrung, N.; Ampawong, S.; Reamtong, O.; Diraphat, P.; Chaicumpa, W.; Indrawattana, N., Human single-chain antibodies that neutralize Pseudomonas aeruginosa-exotoxin A-mediated cellular apoptosis. *Sci Rep* **2019,** *9* (1), 14928. DOI: 10.1038/s41598-019-51089-w.
84. Shadman, Z.; Farajnia, S.; Pazhang, M.; Tohidkia, M.; Rahbarnia, L.; Najavand, S.; Toraby, S., Isolation and characterizations of a novel recombinant scFv antibody against exotoxin A of Pseudomonas aeruginosa. *BMC Infect Dis* **2021,** *21* (1), 300. DOI: 10.1186/s12879-021-05969-0.
85. Chan, R.; Buckley, P. T.; O'Malley, A.; Sause, W. E.; Alonzo, F.; Lubkin, A.; Boguslawski, K. M.; Payne, A.; Fernandez, J.; Strohl, W. R.; Whitaker, B.; Lynch, A. S.; Torres, V. J., Identification of biologic agents to neutralize the bicomponent leukocidins of. *Sci Transl Med* **2019,** *11* (475). DOI: 10.1126/scitranslmed.aat0882.
86. Simeon, R.; Jiang, M.; Chamoun-Emanuelli, A. M.; Yu, H.; Zhang, Y.; Meng, R.; Peng, Z.; Jakana, J.; Zhang, J.; Feng, H.; Chen, Z., Selection and characterization of ultrahigh potency designed ankyrin repeat protein inhibitors of C. difficile toxin B. *PLoS Biol* **2019,** *17* (6), e3000311. DOI: 10.1371/journal.pbio.3000311.
87. Peng, Z.; Simeon, R.; Mitchell, S. B.; Zhang, J.; Feng, H.; Chen, Z., Designed ankyrin repeat protein (DARPin) neutralizers of TcdB from Clostridium difficile ribotype 027. *mSphere* **2019,** *4* (5). DOI: 10.1128/mSphere.00596-19.
88. Chevalier, A.; Silva, D. A.; Rocklin, G. J.; Hicks, D. R.; Vergara, R.; Murapa, P.; Bernard, S. M.; Zhang, L.; Lam, K. H.; Yao, G.; Bahl, C. D.; Miyashita, S. I.; Goreshnik, I.; Fuller, J. T.; Koday, M. T.; Jenkins, C. M.; Colvin, T.; Carter, L.; Bohn, A.; Bryan, C. M.; Fernández-Velasco, D. A.; Stewart, L.; Dong, M.; Huang, X.; Jin, R.; Wilson, I. A.; Fuller, D. H.; Baker, D., Massively parallel de novo protein design for targeted therapeutics. *Nature* **2017,** *550* (7674), 74-79. DOI: 10.1038/nature23912.
89. Jiang, L.; Zhu, Y.; Luan, P.; Xu, J.; Ru, G.; Fu, J. G.; Sang, N.; Xiong, Y.; He, Y.; Lin, G. Q.; Wang, J.; Zhang, J.; Li, R., Bacteria-anchoring hybrid liposome capable of absorbing multiple toxins for antivirulence therapy of Escherichia coli infection. *ACS Nano* **2021,** *15* (3), 4173-4185. DOI: 10.1021/acsnano.0c04800.

90. Xu, J.; Zhang, S.; Zhao, S.; Hu, L., Identification and synthesis of an efficient multivalent E. coli heat labile toxin inhibitor. *Bioorg Med Chem* **2020,** *28* (9), 115436. DOI: 10.1016/j.bmc.2020.115436.
91. Cervin, J.; Boucher, A.; Youn, G.; Björklund, P.; Wallenius, V.; Mottram, L.; Sampson, N. S.; Yrlid, U., Fucose-galactose polymers inhibit cholera toxin binding to fucosylated structures and galactose-dependent intoxication of human enteroids. *ACS Infect Dis* **2020,** *6* (5), 1192-1203. DOI: 10.1021/acsinfecdis.0c00009.
92. Youn, G.; Cervin, J.; Yu, X.; Bhatia, S. R.; Yrlid, U.; Sampson, N. S., Targeting multiple binding sites on cholera toxin B with glycomimetic polymers promotes the formation of protein-polymer aggregates. *Biomacromolecules* **2020,** *21* (12), 4878-4887. DOI: 10.1021/acs.biomac.0c01122.
93. Haksar, D.; Quarles van Ufford, L.; Pieters, R. J., A hybrid polymer to target blood group dependence of cholera toxin. *Org Biomol Chem* **2019,** *18* (1), 52-55. DOI: 10.1039/c9ob02369k.
94. Boesveld, S.; Jans, A.; Rommel, D.; Bartneck, M.; Möller, M.; Elling, L.; Trautwein, C.; Strnad, P.; Kuehne, A. J. C., Microgels sopping up toxins-GM1a-functionalized microgels as scavengers for cholera toxin. *ACS Appl Mater Interfaces* **2019,** *11* (28), 25017-25023. DOI: 10.1021/acsami.9b06413.
95. Haksar, D.; de Poel, E.; van Ufford, L. Q.; Bhatia, S.; Haag, R.; Beekman, J.; Pieters, R. J., Strong inhibition of cholera toxin B subunit by affordable, polymer-based multivalent inhibitors. *Bioconjug Chem* **2019,** *30* (3), 785-792. DOI: 10.1021/acs.bioconjchem.8b00902.
96. Das, S.; Angsantikul, P.; Le, C.; Bao, D.; Miyamoto, Y.; Gao, W.; Zhang, L.; Eckmann, L., Neutralization of cholera toxin with nanoparticle decoys for treatment of cholera. *PLoS Negl Trop Dis* **2018,** *12* (2), e0006266. DOI: 10.1371/journal.pntd.0006266.
97. Wands, A. M.; Cervin, J.; Huang, H.; Zhang, Y.; Youn, G.; Brautigam, C. A.; Matson Dzebo, M.; Björklund, P.; Wallenius, V.; Bright, D. K.; Bennett, C. S.; Wittung-Stafshede, P.; Sampson, N. S.; Yrlid, U.; Kohler, J. J., Fucosylated molecules competitively interfere with cholera toxin binding to host cells. *ACS Infect Dis* **2018,** *4* (5), 758-770. DOI: 10.1021/acsinfecdis.7b00085.
98. Sarwar, S.; Ali, A.; Pal, M.; Chakrabarti, P., Zinc oxide nanoparticles provide anti-cholera activity by disrupting the interaction of cholera toxin with the human GM1 receptor. *J Biol Chem* **2017,** *292* (44), 18303-18311. DOI: 10.1074/jbc.M117.793240.
99. Zuilhof, H., Fighting Cholera one-on-one: The development and efficacy of multivalent cholera-toxin-binding molecules. *Acc Chem Res* **2016,** *49* (2), 274-85. DOI: 10.1021/acs.accounts.5b00480.
100. Yu, R. K.; Usuki, S.; Itokazu, Y.; Wu, H. C., Novel GM1 ganglioside-like peptide mimics prevent the association of cholera toxin to human intestinal epithelial cells in vitro. *Glycobiology* **2016,** *26* (1), 63-73. DOI: 10.1093/glycob/cwv080.
101. Keller, M. D.; Ching, K. L.; Liang, F. X.; Dhabaria, A.; Tam, K.; Ueberheide, B. M.; Unutmaz, D.; Torres, V. J.; Cadwell, K., Decoy exosomes provide protection against bacterial toxins. *Nature* **2020,** *579* (7798), 260-264. DOI: 10.1038/s41586-020-2066-6.
102. Subramanian, K.; Iovino, F.; Tsikourkitoudi, V.; Merkl, P.; Ahmed, S.; Berry, S. B.; Aschtgen, M. S.; Svensson, M.; Bergman, P.; Sotiriou, G. A.; Henriques-Normark, B., Mannose receptor-derived peptides neutralize pore-forming toxins and reduce inflammation and development of pneumococcal disease. *EMBO Mol Med* **2020,** *12* (11), e12695. DOI: 10.15252/emmm.202012695.

103. Besançon, H.; Babiychuk, V.; Larpin, Y.; Köffel, R.; Schittny, D.; Brockhus, L.; Hathaway, L. J.; Sendi, P.; Draeger, A.; Babiychuk, E., Tailored liposomal nanotraps for the treatment of Streptococcal infections. *J Nanobiotechnology* **2021,** *19* (1), 46. DOI: 10.1186/s12951-021-00775-x.
104. Chhabria, V.; Beeton, S., Development of nanosponges from erythrocyte ghosts for removal of streptolysin-O from mammalian blood. *Nanomedicine (Lond)* **2016,** *11* (21), 2797-2807. DOI: 10.2217/nnm-2016-0180.
105. Zhang, Y.; Zhang, J.; Chen, W.; Angsantikul, P.; Spiekermann, K. A.; Fang, R. H.; Gao, W.; Zhang, L., Erythrocyte membrane-coated nanogel for combinatorial antivirulence and responsive antimicrobial delivery against Staphylococcus aureus infection. *J Control Release* **2017,** *263*, 185-191. DOI: 10.1016/j.jconrel.2017.01.016.
106. Chen, Y.; Chen, M.; Zhang, Y.; Lee, J. H.; Escajadillo, T.; Gong, H.; Fang, R. H.; Gao, W.; Nizet, V.; Zhang, L., Broad-spectrum neutralization of pore-forming toxins with human erythrocyte membrane-coated nanosponges. *Adv Healthc Mater* **2018,** *7* (13), e1701366. DOI: 10.1002/adhm.201701366.
107. Escajadillo, T.; Olson, J.; Luk, B. T.; Zhang, L.; Nizet, V., A red blood cell membrane-camouflaged nanoparticle counteracts streptolysin. *Front Pharmacol* **2017,** *8*, 477. DOI: 10.3389/fphar.2017.00477.
108. Zhang, Y.; Gao, W.; Chen, Y.; Escajadillo, T.; Ungerleider, J.; Fang, R. H.; Christman, K.; Nizet, V.; Zhang, L., Self-assembled colloidal gel using cell membrane-coated nanosponges as building blocks. *ACS Nano* **2017,** *11* (12), 11923-11930. DOI: 10.1021/acsnano.7b06968.
109. Wang, K.; Wu, D.; Chen, Z.; Zhang, X.; Yang, X.; Yang, C. J.; Lan, X., Inhibition of the superantigenic activities of Staphylococcal enterotoxin A by an aptamer antagonist. *Toxicon* **2016,** *119*, 21-7. DOI: 10.1016/j.toxicon.2016.05.006.
110. Koo, J.; Escajadillo, T.; Zhang, L.; Nizet, V.; Lawrence, S. M., Erythrocyte-coated nanoparticles block cytotoxic effects of group B Streptococcus β-Hemolysin/Cytolysin. *Front Pediatr* **2019,** *7*, 410. DOI: 10.3389/fped.2019.00410.
111. LaGrow, A. L.; Coburn, P. S.; Miller, F. C.; Land, C.; Parkunan, S. M.; Luk, B. T.; Gao, W.; Zhang, L.; Callegan, M. C., A novel biomimetic nanosponge protects the retina from the Enterococcus faecalis cytolysin. *mSphere* **2017,** *2* (6). DOI: 10.1128/mSphere.00335-17.
112. Zhang, S.; Lu, X.; Wang, B.; Zhang, G.; Liu, M.; Geng, S.; Sun, L.; An, J.; Zhang, Z.; Zhang, H., A soft anti-virulence liposome realizing the explosive release of antibiotics at an infectious site to improve antimicrobial therapy. *J Mater Chem B* **2021,** *9* (1), 147-158. DOI: 10.1039/d0tb02255a.
113. Pang, X.; Liu, X.; Cheng, Y.; Zhang, C.; Ren, E.; Liu, C.; Zhang, Y.; Zhu, J.; Chen, X.; Liu, G., Sono-immunotherapeutic nanocapturer to combat multidrug-resistant bacterial infections. *Adv Mater* **2019,** *31* (35), e1902530. DOI: 10.1002/adma.201902530.
114. Esteban-Fernández de Ávila, B.; Angsantikul, P.; Ramírez-Herrera, D. E.; Soto, F.; Teymourian, H.; Dehaini, D.; Chen, Y.; Zhang, L.; Wang, J., Hybrid biomembrane-functionalized nanorobots for concurrent removal of pathogenic bacteria and toxins. *Sci Robot* **2018,** *3* (18). DOI: 10.1126/scirobotics.aat0485.
115. Sedighian, H.; Halabian, R.; Amani, J.; Heiat, M.; Taheri, R. A.; Imani Fooladi, A. A., Manufacturing of a novel double-function ssDNA aptamer for sensitive diagnosis and efficient neutralization of SEA. *Anal Biochem* **2018,** *548*, 69-77. DOI: 10.1016/j.ab.2018.02.017.

116. Li, W.; Geng, X.; Liu, D.; Li, Z., Near-infrared light-enhanced protease-conjugated gold nanorods as a photothermal antimicrobial agent for elimination of exotoxin and biofilms. *Int J Nanomedicine* **2019,** *14*, 8047-8058. DOI: 10.2147/IJN.S212750.
117. Wu, X.; Li, Y.; Raza, F.; Wang, X.; Zhang, S.; Rong, R.; Qiu, M.; Su, J., Red blood cell membrane-camouflaged tedizolid phosphate-loaded PLGA nanoparticles for bacterial-infection therapy. *Pharmaceutics* **2021,** *13* (1). DOI: 10.3390/pharmaceutics13010099.
118. Lin, A.; Liu, Y.; Zhu, X.; Chen, X.; Liu, J.; Zhou, Y.; Qin, X., Bacteria-responsive biomimetic selenium nanosystem for multidrug-resistant bacterial infection detection and inhibition. *ACS Nano* **2019,** *13* (12), 13965-13984. DOI: 10.1021/acsnano.9b05766.
119. Chen, Y.; Zhang, Y.; Chen, M.; Zhuang, J.; Fang, R. H.; Gao, W.; Zhang, L., Biomimetic nanosponges suppress in vivo lethality induced by the whole secreted proteins of pathogenic bacteria. *Small* **2019,** *15* (6), e1804994. DOI: 10.1002/smll.201804994.
120. Coburn, P. S.; Miller, F. C.; LaGrow, A. L.; Land, C.; Mursalin, H.; Livingston, E.; Amayem, O.; Chen, Y.; Gao, W.; Zhang, L.; Callegan, M. C., Disarming pore-forming toxins with biomimetic nanosponges in intraocular infections. *mSphere* **2019,** *4* (3). DOI: 10.1128/mSphere.00262-19.
121. Heine, V.; Boesveld, S.; Pelantová, H.; Křen, V.; Trautwein, C.; Strnad, P.; Elling, L., Identifying Efficient Clostridium difficile toxin A binders with a multivalent neo-glycoprotein glycan library. *Bioconjug Chem* **2019,** *30* (9), 2373-2383. DOI: 10.1021/acs.bioconjchem.9b00486.
122. Andersen, K. K.; Strokappe, N. M.; Hultberg, A.; Truusalu, K.; Smidt, I.; Mikelsaar, R. H.; Mikelsaar, M.; Verrips, T.; Hammarström, L.; Marcotte, H., Neutralization of Clostridium difficile toxin B mediated by engineered Lactobacilli that produce single-domain antibodies. *Infect Immun* **2016,** *84* (2), 395-406. DOI: 10.1128/IAI.00870-15.
123. Cherian, R. M.; Jin, C.; Liu, J.; Karlsson, N. G.; Holgersson, J., Recombinant mucin type fusion proteins with a Galα1,3Gal substitution as Clostridium difficile toxin A inhibitors. *Infect Immun* **2016,** *84* (10), 2842-52. DOI: 10.1128/IAI.00341-16.
124. Pohlentz, G.; Steil, D.; Rubin, D.; Mellmann, A.; Karch, H.; Müthing, J., Pectin-derived neoglycolipids: Tools for differentiation of Shiga toxin subtypes and inhibitors of Shiga toxin-mediated cellular injury. *Carbohydr Polym* **2019,** *212*, 323-333. DOI: 10.1016/j.carbpol.2019.02.039.
125. Li, C. H.; Bai, Y. L.; Chen, Y. C., Inhibition of the lethality of Shiga-like toxin-1 by functional gold nanoparticles. *Artif Cells Nanomed Biotechnol* **2018,** *46* (sup1), 841-851. DOI: 10.1080/21691401.2018.1438449.
126. Matsuoka, K.; Nishikawa, K.; Goshu, Y.; Koyama, T.; Hatano, K.; Matsushita, T.; Watanabe-Takahashi, M.; Natori, Y.; Terunuma, D., Synthetic construction of sugar-amino acid hybrid polymers involving globotriaose or lactose and evaluation of their biological activities against Shiga toxins produced by Escherichia coli O157:H7. *Bioorg Med Chem* **2018,** *26* (22), 5792-5803. DOI: 10.1016/j.bmc.2018.10.023.
127. Zhang, P.; Paszkiewicz, E.; Wang, Q.; Sadowska, J. M.; Kitov, P. I.; Bundle, D. R.; Ling, C. C., Clustering of P^k-trisaccharides on amphiphilic cyclodextrin reveals unprecedented affinity for the Shiga-like toxin Stx2. *Chem Commun (Camb)* **2017,** *53* (76), 10528-10531. DOI: 10.1039/c7cc06299k.

128. Mitsui, T.; Watanabe-Takahashi, M.; Shimizu, E.; Zhang, B.; Funamoto, S.; Yamasaki, S.; Nishikawa, K., Affinity-based screening of tetravalent peptides identifies subtype-selective neutralizers of Shiga Toxin 2d, a highly virulent subtype, by targeting a unique amino acid involved in its receptor recognition. *Infect Immun* **2016,** *84* (9), 2653-61. DOI: 10.1128/IAI.00149-16.
129. Li, T.; Tu, W.; Liu, Y.; Zhou, P.; Cai, K.; Li, Z.; Liu, X.; Ning, N.; Huang, J.; Wang, S.; Wang, H., A potential therapeutic peptide-based neutralizer that potently inhibits Shiga toxin 2 in vitro and in vivo. *Sci Rep* **2016,** *6*, 21837. DOI: 10.1038/srep21837.
130. Amezcua, M.; Cruz, R. S.; Ku, A.; Moran, W.; Ortega, M. E.; Salzameda, N. T., Discovery of dipeptides as potent botulinum neurotoxin A light-chain inhibitors. *ACS Med Chem Lett* **2021,** *12* (2), 295-301. DOI: 10.1021/acsmedchemlett.0c00674.
131. McNutt, P. M.; Vazquez-Cintron, E. J.; Tenezaca, L.; Ondeck, C. A.; Kelly, K. E.; Mangkhalakhili, M.; Machamer, J. B.; Angeles, C. A.; Glotfelty, E. J.; Cika, J.; Benjumea, C. H.; Whitfield, J. T.; Band, P. A.; Shoemaker, C. B.; Ichtchenko, K., Neuronal delivery of antibodies has therapeutic effects in animal models of botulism. *Sci Transl Med* **2021,** *13* (575). DOI: 10.1126/scitranslmed.abd7789.
132. Al-Saleem, F. H.; Sharma, R.; Puligedda, R. D.; Elias, M.; Kattala, C. D.; Simon, P. M.; Simpson, L. L.; Dessain, S. K., RBC adherence of immune complexes containing botulinum toxin improves neutralization and macrophage uptake. *Toxins (Basel)* **2017,** *9* (5). DOI: 10.3390/toxins9050173.
133. Huang, N. J.; Pishesha, N.; Mukherjee, J.; Zhang, S.; Deshycka, R.; Sudaryo, V.; Dong, M.; Shoemaker, C. B.; Lodish, H. F., Genetically engineered red cells expressing single domain camelid antibodies confer long-term protection against botulinum neurotoxin. *Nat Commun* **2017,** *8* (1), 423. DOI: 10.1038/s41467-017-00448-0.
134. Robinson, T. M.; Jicsinszky, L.; Karginov, A. V.; Karginov, V. A., Inhibition of Clostridium perfringens epsilon toxin by β-cyclodextrin derivatives. *Int J Pharm* **2017,** *531* (2), 714-717. DOI: 10.1016/j.ijpharm.2017.07.070.
135. Biondi, E.; Lane, J. D.; Das, D.; Dasgupta, S.; Piccirilli, J. A.; Hoshika, S.; Bradley, K. M.; Krantz, B. A.; Benner, S. A., Laboratory evolution of artificially expanded DNA gives redesignable aptamers that target the toxic form of anthrax protective antigen. *Nucleic Acids Res* **2016,** *44* (20), 9565-9577. DOI: 10.1093/nar/gkw890.
136. Momben Abolfath, S.; Kolberg, M.; Karginov, V. A.; Leppla, S. H.; Nestorovich, E. M., Exploring the nature of cationic blocker recognition by the anthrax toxin channel. *Biophys J* **2019,** *117* (9), 1751-1763. DOI: 10.1016/j.bpj.2019.08.041.
137. Xiong, Y.; Karuppanan, K.; Bernardi, A.; Li, Q.; Kommineni, V.; Dandekar, A. M.; Lebrilla, C. B.; Faller, R.; McDonald, K. A.; Nandi, S., Effects of N-glycosylation on the structure, function, and stability of a plant-made Fc-fusion anthrax decoy protein. *Front Plant Sci* **2019,** *10*, 768. DOI: 10.3389/fpls.2019.00768.
138. Lahousse, M.; Park, H. C.; Lee, S. C.; Ha, N. R.; Jung, I. P.; Schlesinger, S. R.; Shackelford, K.; Yoon, M. Y.; Kim, S. K., Inhibition of anthrax lethal factor by ssDNA aptamers. *Arch Biochem Biophys* **2018,** *646*, 16-23. DOI: 10.1016/j.abb.2018.03.028.
139. Li, L.; Guo, Q.; Liu, J.; Zhang, J.; Yin, Y.; Dong, D.; Fu, L.; Xu, J.; Chen, W., Recombinant HSA-CMG2 is a promising anthrax toxin inhibitor. *Toxins (Basel)* **2016,** *8* (1). DOI: 10.3390/toxins8010028.
140. Krueger, E.; Hayes, S.; Chang, E. H.; Yutuc, S.; Brown, A. C., Receptor-based peptides for inhibition of leukotoxin activity. *ACS Infect Dis* **2018,** *4* (7), 1073-1081. DOI: 10.1021/acsinfecdis.7b00230.

141. Zou, G.; de Leeuw, E., Neutralization of Pseudomonas auruginosa Exotoxin A by human neutrophil peptide 1. *Biochem Biophys Res Commun* **2018,** *501* (2), 454-457. DOI: 10.1016/j.bbrc.2018.05.011.
142. Reyes-Robles, T.; Lubkin, A.; Alonzo, F.; Lacy, D. B.; Torres, V. J., Exploiting dominant-negative toxins to combat Staphylococcus aureus pathogenesis. *EMBO Rep* **2016,** *17* (3), 428-40. DOI: 10.15252/embr.201540994.
143. Bender, K. O.; Garland, M.; Ferreyra, J. A.; Hryckowian, A. J.; Child, M. A.; Puri, A. W.; Solow-Cordero, D. E.; Higginbottom, S. K.; Segal, E.; Banaei, N.; Shen, A.; Sonnenburg, J. L.; Bogyo, M., A small-molecule antivirulence agent for treating Clostridium difficile infection. *Sci Transl Med* **2015,** *7* (306), 306ra148. DOI: 10.1126/scitranslmed.aac9103.
144. Garland, M.; Hryckowian, A. J.; Tholen, M.; Bender, K. O.; Van Treuren, W. W.; Loscher, S.; Sonnenburg, J. L.; Bogyo, M., The clinical drug Ebselen attenuates inflammation and promotes microbiome recovery in mice after antibiotic treatment for CDI. *Cell Rep Med* **2020,** *1* (1). DOI: 10.1016/j.xcrm.2020.100005.
145. Ivarsson, M. E.; Durantie, E.; Huberli, C.; Huwiler, S.; Hegde, C.; Friedman, J.; Altamura, F.; Lu, J.; Verdu, E. F.; Bercik, P.; Logan, S. M.; Chen, W.; Leroux, J. C.; Castagner, B., Small-molecule allosteric triggers of Clostridium difficile toxin B auto-proteolysis as a therapeutic strategy. *Cell Chem Biol* **2019,** *26* (1), 17-26.e13. DOI: 10.1016/j.chembiol.2018.10.002.
146. Letourneau, J. J.; Stroke, I. L.; Hilbert, D. W.; Sturzenbecker, L. J.; Marinelli, B. A.; Quintero, J. G.; Sabalski, J.; Ma, L.; Diller, D. J.; Stein, P. D.; Webb, M. L., Identification and initial optimization of inhibitors of Clostridium difficile (C. difficile) toxin B (TcdB). *Bioorg Med Chem Lett* **2018,** *28* (4), 756-761. DOI: 10.1016/j.bmcl.2018.01.005.
147. Letourneau, J. J.; Stroke, I. L.; Hilbert, D. W.; Cole, A. G.; Sturzenbecker, L. J.; Marinelli, B. A.; Quintero, J. G.; Sabalski, J.; Li, Y.; Ma, L.; Pechik, I.; Stein, P. D.; Webb, M. L., Synthesis and SAR studies of novel benzodiazepinedione-based inhibitors of Clostridium difficile (C. difficile) toxin B (TcdB). *Bioorg Med Chem Lett* **2018,** *28* (23-24), 3601-3605. DOI: 10.1016/j.bmcl.2018.10.047.
148. Stroke, I. L.; Letourneau, J. J.; Miller, T. E.; Xu, Y.; Pechik, I.; Savoly, D. R.; Ma, L.; Sturzenbecker, L. J.; Sabalski, J.; Stein, P. D.; Webb, M. L.; Hilbert, D. W., Treatment of Clostridium difficile infection with a small-molecule inhibitor of toxin UDP-glucose hydrolysis activity. *Antimicrob Agents Chemother* **2018,** *62* (5). DOI: 10.1128/AAC.00107-18.
149. Zhu, Z.; Schnell, L.; Müller, B.; Müller, M.; Papatheodorou, P.; Barth, H., The antibiotic Bacitracin protects human intestinal epithelial cells and stem cell-derived intestinal organoids from Clostridium difficile toxin TcdB. *Stem Cells Int* **2019,** *2019*, 4149762. DOI: 10.1155/2019/4149762.
150. Ashok, Y.; Miettinen, M.; Oliveira, D. K. H.; Tamirat, M. Z.; Näreoja, K.; Tiwari, A.; Hottiger, M. O.; Johnson, M. S.; Lehtiö, L.; Pulliainen, A. T., Discovery of compounds inhibiting the ADP-ribosyltransferase activity of pertussis toxin. *ACS Infect Dis* **2020,** *6* (4), 588-602. DOI: 10.1021/acsinfecdis.9b00412.
151. Lin, L.; Olson, M. E.; Sugane, T.; Turner, L. D.; Tararina, M. A.; Nielsen, A. L.; Kurbanov, E. K.; Pellett, S.; Johnson, E. A.; Cohen, S. M.; Allen, K. N.; Janda, K. D., Catch and anchor approach to combat both toxicity and longevity of botulinum toxin A. *J Med Chem* **2020,** *63* (19), 11100-11120. DOI: 10.1021/acs.jmedchem.0c01006.
152. Garland, M.; Babin, B. M.; Miyashita, S. I.; Loscher, S.; Shen, Y.; Dong, M.; Bogyo, M., Covalent modifiers of botulinum neurotoxin counteract toxin persistence. *ACS Chem Biol* **2019,** *14* (1), 76-87. DOI: 10.1021/acschembio.8b00937.

153. Chauhan, R.; Chauhan, V.; Sonkar, P.; Vimal, M.; Dhaked, R. K., Targeted 8-hydroxyquinoline fragment based small molecule drug discovery against neglected botulinum neurotoxin type F. *Bioorg Chem* **2019,** *92*, 103297. DOI: 10.1016/j.bioorg.2019.103297.
154. Patel, K. B.; Cai, S.; Adler, M.; Singh, B. K.; Parmar, V. S.; Singh, B. R., Natural compounds and their analogues as potent antidotes against the most poisonous bacterial toxin. *Appl Environ Microbiol* **2018,** *84* (24). DOI: 10.1128/AEM.01280-18.
155. Jacobson, A. R.; Adler, M.; Silvaggi, N. R.; Allen, K. N.; Smith, G. M.; Fredenburg, R. A.; Stein, R. L.; Park, J. B.; Feng, X.; Shoemaker, C. B.; Deshpande, S. S.; Goodnough, M. C.; Malizio, C. J.; Johnson, E. A.; Pellett, S.; Tepp, W. H.; Tzipori, S., Small molecule metalloprotease inhibitor with in vitro, ex vivo and in vivo efficacy against botulinum neurotoxin serotype A. *Toxicon* **2017,** *137*, 36-47. DOI: 10.1016/j.toxicon.2017.06.016.
156. Kumar, G.; Agarwal, R.; Swaminathan, S., Discovery of a fluorene class of compounds as inhibitors of botulinum neurotoxin serotype E by virtual screening. *Chem Commun (Camb)* **2012,** *48* (18), 2412-4. DOI: 10.1039/c2cc17158a.
157. Kumar, G.; Agarwal, R.; Swaminathan, S., Small molecule non-peptide inhibitors of botulinum neurotoxin serotype E: Structure-activity relationship and a pharmacophore model. *Bioorg Med Chem* **2016,** *24* (18), 3978-3985. DOI: 10.1016/j.bmc.2016.06.036.
158. Thompson, J. C.; Dao, W. T.; Ku, A.; Rodriguez-Beltran, S. L.; Amezcua, M.; Palomino, A. Y.; Lien, T.; Salzameda, N. T., Synthesis and activity of isoleucine sulfonamide derivatives as novel botulinum neurotoxin serotype A light chain inhibitors. *Bioorg Med Chem* **2020,** *28* (18), 115659. DOI: 10.1016/j.bmc.2020.115659.
159. Yalamanchili, C.; Manda, V. K.; Chittiboyina, A. G.; Guernieri, R. L.; Harrell, W. A.; Webb, R. P.; Smith, L. A.; Khan, I. A., Utilizing Ayurvedic literature for the identification of novel phytochemical inhibitors of botulinum neurotoxin A. *J Ethnopharmacol* **2017,** *197*, 211-217. DOI: 10.1016/j.jep.2016.07.069.
160. Minnow, Y. V.; Goldberg, R.; Tummalapalli, S. R.; Rotella, D. P.; Goodey, N. M., Mechanism of inhibition of botulinum neurotoxin type A light chain by two quinolinol compounds. *Arch Biochem Biophys* **2017,** *618*, 15-22. DOI: 10.1016/j.abb.2017.01.006.
161. Bompiani, K. M.; Caglič, D.; Krutein, M. C.; Benoni, G.; Hrones, M.; Lairson, L. L.; Bian, H.; Smith, G. R.; Dickerson, T. J., High-throughput screening uncovers novel botulinum neurotoxin inhibitor chemotypes. *ACS Comb Sci* **2016,** *18* (8), 461-74. DOI: 10.1021/acscombsci.6b00033.
162. Li, G.; Shen, X.; Wei, Y.; Si, X.; Deng, X.; Wang, J., Quercetin reduces Streptococcus suis virulence by inhibiting suilysin activity and inflammation. *Int Immunopharmacol* **2019,** *69*, 71-78. DOI: 10.1016/j.intimp.2019.01.017.
163. Li, G.; Lu, G.; Qi, Z.; Li, H.; Wang, L.; Wang, Y.; Liu, B.; Niu, X.; Deng, X.; Wang, J., Morin attenuates Streptococcus suis pathogenicity in mice by neutralizing suilysin activity. *Front Microbiol* **2017,** *8*, 460. DOI: 10.3389/fmicb.2017.00460.
164. Lv, Q.; Zhang, P.; Quan, P.; Cui, M.; Liu, T.; Yin, Y.; Chi, G., Quercetin, a pneumolysin inhibitor, protects mice against Streptococcus pneumoniae infection. *Microb Pathog* **2020,** *140*, 103934. DOI: 10.1016/j.micpath.2019.103934.
165. Li, H.; Zhao, X.; Deng, X.; Wang, J.; Song, M.; Niu, X.; Peng, L., Insights into structure and activity of natural compound inhibitors of pneumolysin. *Sci Rep* **2017,** *7*, 42015. DOI: 10.1038/srep42015.

166. Song, M.; Teng, Z.; Li, M.; Niu, X.; Wang, J.; Deng, X., Epigallocatechin gallate inhibits Streptococcus pneumoniae virulence by simultaneously targeting pneumolysin and sortase A. *J Cell Mol Med* **2017,** *21* (10), 2586-2598. DOI: 10.1111/jcmm.13179.

167. Qi, Z.; Guo, Y.; Zhang, H.; Yu, Q.; Zhang, P., Betulin attenuates pneumolysin-induced cell injury and DNA damage. *J Appl Microbiol* **2021,** *130* (3), 843-851. DOI: 10.1111/jam.14769.

168. Zhao, X.; Zhou, Y.; Wang, L.; Li, M.; Shi, D.; Li, D.; Wang, J., Shikonin alleviates the biotoxicity produced by pneumococcal pneumolysin. *Life Sci* **2017,** *177*, 1-7. DOI: 10.1016/j.lfs.2017.04.002.

169. Sogawa, K.; Kobayashi, M.; Suzuki, J.; Sanda, A.; Kodera, Y.; Fukuyama, M., Inhibitory activity of hydroxytyrosol against Streptolysin O-induced hemolysis. *Biocontrol Sci* **2018,** *23* (2), 77-80. DOI: 10.4265/bio.23.77.

170. Lu, G.; Xu, L.; Zhang, T.; Deng, X.; Wang, J., A potential bio-control agent from baical skullcap root against listeriosis via the inhibition of sortase A and listeriolysin O. *J Cell Mol Med* **2019,** *23* (3), 2042-2051. DOI: 10.1111/jcmm.14110.

171. Liu, B.; Teng, Z.; Wang, J.; Lu, G.; Deng, X.; Li, L., Inhibition of listeriolysin O oligomerization by lutein prevents Listeria monocytogenes infection. *Fitoterapia* **2017,** *116*, 45-50. DOI: 10.1016/j.fitote.2016.11.012.

172. La Pietra, L.; Hudel, M.; Pillich, H.; Abu Mraheil, M.; Berisha, B.; Aden, S.; Hodnik, V.; Lochnit, G.; Rafiq, A.; Perniss, A.; Anderluh, G.; Chakraborty, T., Phosphocholine antagonizes listeriolysin O-induced host cell responses of Listeria monocytogenes. *J Infect Dis* **2020,** *222* (9), 1505-1516. DOI: 10.1093/infdis/jiaa022.

173. Wang, T.; Zhang, P.; Lv, H.; Deng, X.; Wang, J., A Natural dietary flavone myricetin as an α-hemolysin inhibitor for controlling. *Front Cell Infect Microbiol* **2020,** *10*, 330. DOI: 10.3389/fcimb.2020.00330.

174. Jiang, L.; Yi, T.; Shen, Z.; Teng, Z.; Wang, J., Aloe-emodin attenuates Staphylococcus aureus pathogenicity by interfering with the oligomerization of α-toxin. *Front Cell Infect Microbiol* **2019,** *9*, 157. DOI: 10.3389/fcimb.2019.00157.

175. Liu, J.; Kozhaya, L.; Torres, V. J.; Unutmaz, D.; Lu, M., Structure-based discovery of a small-molecule inhibitor of methicillin-resistant Staphylococcus aureus virulence. *J Biol Chem* **2020,** *295* (18), 5944-5959. DOI: 10.1074/jbc.RA120.012697.

176. Kany, A. M.; Sikandar, A.; Haupenthal, J.; Yahiaoui, S.; Maurer, C. K.; Proschak, E.; Köhnke, J.; Hartmann, R. W., Binding mode characterization and early in vivo evaluation of fragment-like thiols as inhibitors of the virulence factor LasB from Pseudomonas aeruginosa. *ACS Infect Dis* **2018,** *4* (6), 988-997. DOI: 10.1021/acsinfecdis.8b00010.

177. Kany, A. M.; Sikandar, A.; Yahiaoui, S.; Haupenthal, J.; Walter, I.; Empting, M.; Köhnke, J.; Hartmann, R. W., Tackling Pseudomonas aeruginosa virulence by a hydroxamic acid-based LasB inhibitor. *ACS Chem Biol* **2018,** *13* (9), 2449-2455. DOI: 10.1021/acschembio.8b00257.

178. Zhu, J.; Cai, X.; Harris, T. L.; Gooyit, M.; Wood, M.; Lardy, M.; Janda, K. D., Disarming Pseudomonas aeruginosa virulence factor LasB by leveraging a Caenorhabditis elegans infection model. *Chem Biol* **2015,** *22* (4), 483-491. DOI: 10.1016/j.chembiol.2015.03.012.

179. Schönauer, E.; Kany, A. M.; Haupenthal, J.; Hüsecken, K.; Hoppe, I. J.; Voos, K.; Yahiaoui, S.; Elsässer, B.; Ducho, C.; Brandstetter, H.; Hartmann, R. W., Discovery of a potent inhibitor class with high selectivity toward clostridial collagenases. *J Am Chem Soc* **2017,** *139* (36), 12696-12703. DOI: 10.1021/jacs.7b06935.

180. Chauhan, V.; Chaudhary, D.; Pathak, U.; Saxena, N.; Dhaked, R. K., In silico discovery and validation of amide based small molecule targeting the enzymatic site of Shiga toxin. *J Med Chem* **2016,** *59* (23), 10763-10773. DOI: 10.1021/acs.jmedchem.6b01517.

181. Szuster-Ciesielska, A.; Urban-Chmiel, R.; Wernicki, A.; Mascaron, L.; Wasak, M.; Bousquet, E., Evaluation of the ability of colistin, amoxicillin (components of Potencil ®), and fluoroquinolones to attenuate bacterial endotoxin- and Shiga exotoxin-mediated cytotoxicity-In vitro studies. *J Vet Pharmacol Ther* **2019,** *42* (1), 85-103. DOI: 10.1111/jvp.12710.

182. Zhang, J.; Liu, S.; Xia, L.; Wen, Z.; Hu, N.; Wang, T.; Deng, X.; He, J.; Wang, J., Verbascoside protects mice from clostridial gas gangrene by inhibiting the activity of alpha toxin and perfringolysin O. *Front Microbiol* **2020,** *11*, 1504. DOI: 10.3389/fmicb.2020.01504.

183. Schnell, L.; Mittler, A. K.; Sadi, M.; Popoff, M. R.; Schwan, C.; Aktories, K.; Mattarei, A.; Azarnia Tehran, D.; Montecucco, C.; Barth, H., EGA protects mammalian cells from Clostridium difficile CDT, Clostridium perfringens Iota toxin and Clostridium botulinum C2 toxin. *Toxins (Basel)* **2016,** *8* (4), 101. DOI: 10.3390/toxins8040101.

184. Schnell, L.; Felix, I.; Müller, B.; Sadi, M.; von Bank, F.; Papatheodorou, P.; Popoff, M. R.; Aktories, K.; Waltenberger, E.; Benz, R.; Weichbrodt, C.; Fauler, M.; Frick, M.; Barth, H., Revisiting an old antibiotic: bacitracin neutralizes binary bacterial toxins and protects cells from intoxication. *FASEB J* **2019,** *33* (4), 5755-5771. DOI: 10.1096/fj.201802453R.

185. Kronhardt, A.; Beitzinger, C.; Barth, H.; Benz, R., Chloroquine analog interaction with C2- and Iota-toxin in vitro and in living cells. *Toxins (Basel)* **2016,** *8* (8). DOI: 10.3390/toxins8080237.

186. Kreidler, A. M.; Benz, R.; Barth, H., Chloroquine derivatives block the translocation pores and inhibit cellular entry of Clostridium botulinum C2 toxin and Bacillus anthracis lethal toxin. *Arch Toxicol* **2017,** *91* (3), 1431-1445. DOI: 10.1007/s00204-016-1716-9.

187. Jiao, G. S.; Kim, S.; Moayeri, M.; Thai, A.; Cregar-Hernandez, L.; McKasson, L.; O'Malley, S.; Leppla, S. H.; Johnson, A. T., Small molecule inhibitors of anthrax edema factor. *Bioorg Med Chem Lett* **2018,** *28* (2), 134-139. DOI: 10.1016/j.bmcl.2017.11.040.

188. Schnell, L.; Mittler, A. K.; Mattarei, A.; Azarnia Tehran, D.; Montecucco, C.; Barth, H., Semicarbazone EGA inhibits uptake of diphtheria toxin into human cells and protects cells from intoxication. *Toxins (Basel)* **2016,** *8* (7). DOI: 10.3390/toxins8070221.

189. Cherubin, P.; Garcia, M. C.; Curtis, D.; Britt, C. B.; Craft, J. W.; Burress, H.; Berndt, C.; Reddy, S.; Guyette, J.; Zheng, T.; Huo, Q.; Quiñones, B.; Briggs, J. M.; Teter, K., Inhibition of cholera toxin and other AB toxins by polyphenolic compounds. *PLoS One* **2016,** *11* (11), e0166477. DOI: 10.1371/journal.pone.0166477.

190. Arzanlou, M., Inhibition of streptococcal pyrogenic exotoxin B using allicin from garlic. *Microb Pathog* **2016,** *93*, 166-71. DOI: 10.1016/j.micpath.2016.02.010.

191. Woehl, J. L.; Kitamura, S.; Dillon, N.; Han, Z.; Edgar, L. J.; Nizet, V.; Wolan, D. W., An irreversible inhibitor to probe the role of Streptococcus pyogenes cysteine protease SpeB in evasion of host complement defenses. *ACS Chem Biol* **2020,** *15* (8), 2060-2069. DOI: 10.1021/acschembio.0c00191.
